# Kiloampere, Variable-Temperature, Critical-Current Measurements of High-Field Superconductors

**DOI:** 10.6028/jres.118.015

**Published:** 2013-08-19

**Authors:** LF Goodrich, N Cheggour, TC Stauffer, BJ Filla, XF Lu

**Affiliations:** 1 Department of Physics, University of Colorado, Boulder, CO 80309; 2 National Institute of Standards and Technology, Boulder, CO 80305

**Keywords:** critical current, helium gas flow, magnesium-boride, niobium-tin, niobium-titanium, superconducting wire, transport current, variable temperature

## Abstract

We review variable-temperature, transport critical-current (*I*_c_) measurements made on commercial superconductors over a range of critical currents from less than 0.1 A to about 1 kA. We have developed and used a number of systems to make these measurements over the last 15 years. Two exemplary variable-temperature systems with coil sample geometries will be described: a probe that is only variable-temperature and a probe that is variable-temperature and variable-strain. The most significant challenge for these measurements is temperature stability, since large amounts of heat can be generated by the flow of high current through the resistive sample fixture. Therefore, a significant portion of this review is focused on the reduction of temperature errors to less than ±0.05 K in such measurements. A key feature of our system is a pre-regulator that converts a flow of liquid helium to gas and heats the gas to a temperature close to the target sample temperature. The pre-regulator is not in close proximity to the sample and it is controlled independently of the sample temperature. This allows us to independently control the total cooling power, and thereby fine tune the sample cooling power at any sample temperature. The same general temperature-control philosophy is used in all of our variable-temperature systems, but the addition of another variable, such as strain, forces compromises in design and results in some differences in operation and protocol. These aspects are analyzed to assess the extent to which the protocols for our systems might be generalized to other systems at other laboratories. Our approach to variable-temperature measurements is also placed in the general context of measurement-system design, and the perceived advantages and disadvantages of design choices are presented. To verify the accuracy of the variable-temperature measurements, we compared critical-current values obtained on a specimen immersed in liquid helium (“*liquid*” or *I*_c liq_) at 5 K to those measured on the same specimen in flowing helium gas (“*gas*” or *I*_c gas_) at the same temperature. These comparisons indicate the temperature control is effective over the superconducting wire length between the voltage taps, and this condition is valid for all types of sample investigated, including Nb-Ti, Nb_3_Sn, and MgB_2_ wires. The *liquid*/*gas* comparisons are used to study the variable-temperature measurement protocol that was necessary to obtain the “*correct*” critical current, which was assumed to be the *I*_c liq_. We also calibrated the magnetoresistance effect of resistive thermometers for temperatures from 4 K to 35 K and magnetic fields from 0 T to 16 T. This calibration reduces systematic errors in the variable-temperature data, but it does not affect the *liquid*/*gas* comparison since the same thermometers are used in both cases.

## 1. Introduction

Variable-temperature measurements (VTM) are needed to determine the temperature margin of superconducting strands. The temperature margin is defined as the difference between the operating temperature and the temperature at which the operating current equals the critical current (*I*_c_). When a magnet is operating, transient excursions in magnetic field (*H*) or current (*I*) are not expected; however, many events or effects, such as wire motion, ac losses, and radiation, can cause transient excursions to higher temperatures (*T*). Hence, temperature margin is a key strand characteristic whether the application is immersed in a cryogen or is cryogen-free.

Many applications require each superconducting strand to carry 100 A or more under operating conditions, so this sets the typical desired current level for these measurements. VTM could be done on samples with cross-sectional areas smaller than needed for an application to reduce the maximum current needed for the characterization. However, there is no guarantee that the results will scale linearly with cross-sectional area, and there are cases where it is known that the properties do not scale. Characterizing the full-size strand over a wide range of magnetic field, temperature, and, in some cases axial strain, provides a data base that can be used to create accurate mathematical models or empirical parameterizations that are needed for magnet design and operation [1].

General descriptions of *I*_c_ measurements made with the sample immersed in a liquid cryogen are found in [2,3]. This paper will focus on the additional concerns for high-current VTM, and some basic information will be provided to put the VTM into context. For both *I*_c_ measurement systems presented, the sample voltage-tap pairs were separated by about 8 cm. Three pairs of voltage taps are placed along the active portion of the sample, and they cover three adjacent segments of the sample. The active length of the sample between the current contacts is at least 30 cm. The relatively long sample reduces current-transfer voltages [4], which makes the acquired voltage-current (*V-I*) curves more reliable. The long separation of voltage taps, and the placement of the taps a distance of at least 30 wire diameters from the current contacts, allows the determination of the *I*_c_ from the *V-I* curve at an electric-field-strength criteria (*E*_c_) as low as 0.01 μV/cm. All *I*_c_ data presented herein utilize a 0.1 μV/cm (10 μV/m) criterion. Typically, the sample steady-state voltage is zero until the current is near *I*_c_; then the voltage increases rapidly with current. The region of the *V-I* curve near *I*_c_ can be approximated by *V* ∝ *I^n^*, where *n* is a constant called the *n*-value. This power-law approximation indicates that the *V-I* curve is nearly linear when plotted on a full-logarithmic scale, and the slope of the data is the *n*-value. *n*-values range from 1, when the sample is in the normal state, to 30 or even 100 when the *I*_c_ is high and the wire is of good quality. The *n*-value is an indication of the steepness of the *V-I* curve and a figure of merit for a superconducting wire.

The total time interval necessary to accumulate a full set of VTM is a factor in how data are acquired. A full data set over a 2-dimensional matrix of magnetic field and temperature can take two days to acquire for one sample. A full data set over a 3-dimensional matrix (*H*, *T*, strain) can take several weeks for one sample. The parameter easiest to change is the magnetic field. The ramp speed of superconducting magnets is limited and some settling time is needed. So, for example changing the field by 1 T can take two minutes. Changing the temperature by 1 K can take 15 minutes or more. So typically, the temperature is set and the magnetic field is ramped through the range where the *I*_c_ is measurable. It can take 1 h to sweep through the fields at one temperature. Changing strain can have a fatiguing effect on the sample, so it is usually changed the least often of the parameters.

Our computer program has evolved to the point where it can autonomously acquire *V-I* curves at various magnetic fields as long as the *I*_c_ is less than 300 A or 400 A and the sample has good thermal stability. The program uses pre-determined voltage and heater targets, settling times for *H* and *T*, and guesses for *I*_c_ and *n*-value. VTM at higher currents are typically done in a more manual mode, as are studies of various heater values at a given field. Because of all of these time factors, we try to limit the time to acquire a *V-I* curve to about 1 minute, including finding the current range of interest for the curve.

Having a high-current capability only increases the field range and measurement time interval. In addition, the higher the current, the more likely it is that the sample will quench (sample abruptly reverting to the normal state), necessitating a waiting period for the temperature controllers to settle before the next measurement attempt. Even without a quench, the higher the current, the longer is the wait after a *V-I* curve for the temperature controllers to settle. Because of these time concerns, we often limit our VTM to around 600 A, even though we can measure up to 1000 A.

The superconductor community has learned that careful measurement methods are needed to obtain consistent results on superconductors because subtle differences in methods can lead to unacceptable measurement biases. Two key international inter-laboratory comparisons of *I*_c_ measurements with the sample in liquid helium have been conducted by Versailles Project on Advanced Materials and Standards (VAMAS) [5–7]. In the first comparison, the coefficients of variation were 8.0 and 20.6 % for *I*_c_ and *n*-value, respectively, on a Nb_3_Sn sample at 12 T. The most significant influence quantity in the first comparison was the strain state of the sample. A standard procedure was introduced into the second comparison and the coefficients of variation were reduced to 2.2 and 7.1 % for *I*_c_ and *n*-value, respectively, on a Nb_3_Sn sample at 12 T. The most significant influence quantity in the second comparison was the magnetic field calibration. There has not been a similar comparison of variable-temperature *I*_c_ measurements, but the increased temperature uncertainty of VTM is expected to be significant.

*I*_c_ measurements can be very deceptive; one can obtain a very repeatable result that is still incorrect. It is a myth that the highest *I*_c_ is always correct. High values that are incorrect can often be the case for VTM as will be shown in this paper. Inconsistent results can lead to problems including: a mistrust of the results of others, unfair advantages in commerce, erroneous feedback in the optimization of conductor performance, and an application not meeting its designed target. In addition, as state-of-the-art low-temperature superconductor (LTS) and high-temperature superconductor (HTS) wires continue to improve and exceed their previous limits, the engineering limits of *I*_c_ measurement procedures are likewise being pushed. So we approach these VTM with a degree of respect and humility.

High-current, variable-temperature measurements have many experimental challenges. *I*_c_ measurements less than a few amperes are discussed beginning in Sec. 4.1. High currents cause variable heating and temperature gradients. Heat will flow down the high-current leads, even if they are vapor cooled. Heat is generated along the current path in the cryostat depending on the local resistance and joints. For resistive portions of the current path, the power goes up as current squared. Voltage drop along the sample causes self-heating when the current is near or above the critical current. It is necessary to raise the current to these levels to measure the critical current, unlike applications that typically try to operate with the current always below the critical current.

Samples are typically 0.5 mm to 0.85 mm in diameter, and the active length between the current contacts is at least 30 cm. For this sample length, it is hard to measure the average or effective temperature of the sample. If a thermometer is mounted next to a portion of the sample, it is hard to thermally anchor the thermometer to the sample. Moreover, during the measurement itself, the local cooling of the sample may change, so the temperature difference between the sample and the thermometer will not be constant. Both of these discrepancies can make the measured temperature not representative of the average temperature over the length of the sample.

Numerous choices need to be made when designing a variable-temperature system, and the choices described here may not be the best in all circumstances. We focused on the problem of measuring LTS such as Nb-Ti [8] and Nb_3_Sn [9], where the temperature dependence of the critical current requires a temperature uncertainty of about 50 mK or less. Measurements on HTS can typically tolerate higher temperature uncertainties. We view our system as a complex platform that can provide valuable reference data for modeling, important performance verifications, and comparisons with other VTM systems. We discuss other custom and commercial VTM systems in a later section of this paper (Sec. 4).

A key part of our approach to VTM is a comparison of *I*_c_ values obtained on a specimen immersed in liquid helium (“*liquid*” or *I*_c liq_) at 5 K to those measured on the same specimen and on the same probe in flowing helium gas (“*gas*” or *I*_c gas_) at the same temperature. For the rest of this paper, these two cases will be referred to as *liquid* (“*Liq*” for short in figures) and *gas*. These comparisons indicate the temperature control of the superconducting wire over the length between the voltage taps is effective. Based on our search of the literature for VTM performed on other laboratories’ systems, no other laboratory reports a *liquid*/*gas* comparison. Our *liquid*/*gas* comparisons were used to study the VTM protocol that was necessary to obtain the “*correct*” critical current, which we assume to be the *I*_c liq_ since the measurement results with the sample in liquid is more reliable. The protocol for the *gas* measurements includes the overall cooling power (related to the gas flow rate) and heater powers near the sample that can be controlled to be independent of each other and independent of critical current and temperature. With the sample immersed in liquid helium, we expect the difference between the temperature measured by the thermometers and the sample temperature to be minimal. The high-gas-flow design of the apparatus also allows us to make measurements at 5 K with the sample cooled by helium gas flow. The normal boiling point of liquid helium is 4.22 K. So it is difficult to use helium gas to cool a sample near this temperature, but many cryostats can use helium to cool a sample to 5 K in *gas*. At our test site, the reduced atmospheric pressure lowers the boiling point to about 4.03 K, which makes it slightly easier to make measurements at 5 K in helium *gas*. The critical temperature of liquid helium is 5.195 K, so 5 K is about the highest temperature at which *liquid*/*gas* comparisons can be made.

A key feature of our VTM system is what we call a pre-regulator that converts a flow of liquid helium to gas and heats the gas to a temperature close to the target sample temperature. The pre-regulator is not in close proximity to the sample, and it is controlled independently of the sample temperature. This allows us to independently control the total cooling power and the local sample cooling power at any sample temperature. The total cooling power is controlled by the liquid helium flow rate that is converted into a helium gas flow. The pre-regulator is strategically placed to use the probe’s steady-state heat load to boil some of the helium and to utilize the heat generated by the upper part of the current leads during high-current testing to heat the helium, replacing some or all of the heater power of the pre-regulator. We will show that the value of critical current measured in *gas* (*I*_c gas_) will depend on the amount of heater power applied in the local region near the sample. Higher heater power near the sample is correlated with more sub-cooling of the sample relative to the thermometers and a higher measured *I*_c gas_. So we conducted a systematic study of measured *I*_c gas_ with different heater powers. This type of study may have direct implications only for our specific apparatus, indicating the protocol (heater values, gas flow rates, etc.) that gives us the agreement between the average sample temperature and the temperatures of the two thermometers. However, this study also suggests an implication for all variable temperature measurements, because in principle they must all be cooled by an overall gas flow or some other large thermal sink external to the sample locale. Simply stated, the implication is: the higher the heater power near the sample, the higher the temperature gradients will be. It is expected that for many VTM systems the sample heater power will increase with sample temperature; and then temperature gradients, and potentially temperature biases, will systematically increase with temperature.

We show results from and compare two different sample probes in this paper. Both of these systems place the sample in the shape of a coil inside a 52-mm bore solenoid magnet with a field rating of 16.5 T at 4.2 K. One probe does only variable-temperature measurements (VTO probe) and the other probe has variable-temperature and variable-strain capability (VTS probe). The same general temperature-control philosophy is used in all of our variable-temperature systems, but adding another variable, such as magnetic-field angle or strain, forces compromises in design and results in some differences in operation and protocol. These aspects are analyzed to assess the extent to which the protocols for our systems might be generalized to other systems at other laboratories.

**Fig. 1:**
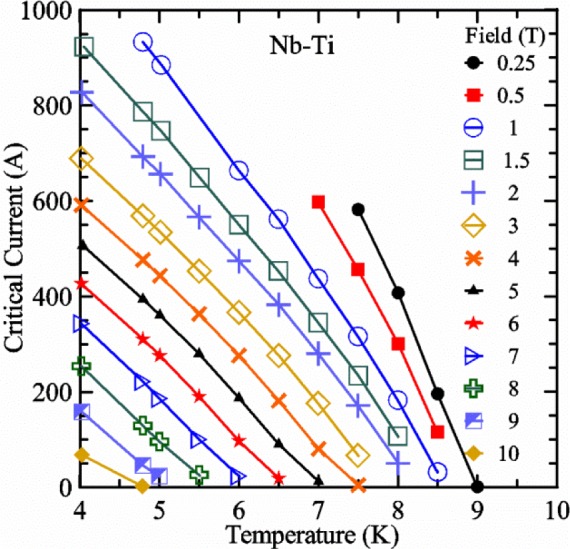
Critical current versus temperature at various magnetic fields for a 0.76 mm diameter Nb-Ti sample.

Most of the data presented in this paper were measured on two samples: one Nb-Ti wire and one Nb_3_Sn wire (Nb_3_Sn #1). Table 1 shows some of the parameters of each wire. All of the wires were commercially produced, multifilamentary wire. The Nb-Ti wire had a diameter of 0.76 mm and a non-Cu fraction of 0.435 (Cu/Nb-Ti ratio of 1.3). It had an *I*_c_ of 507 A and an *n*-value of 54 at 5 T and 4.0 K. Nb_3_Sn #1 had a diameter of 0.82 mm and a non-Cu fraction of 0.476 (Cu/non-Cu ratio of 1.1). It had an *I*_c_ of 279 A and an *n*-value of 61 at 12 T and 4.0 K. Nb_3_Sn #1 was made for the International Thermonuclear Experimental Reactor (ITER) fusion energy project. *I*_c_(*T*) at various magnetic fields for these samples and two additional samples are shown in Figs. 1 (Nb-Ti), 2 (Nb_3_Sn #1), 3 (Nb_3_Sn #2), and 4 (MgB_2_). *I*_c_(*T*) is fairly linear at a given magnetic field, except where the *I*_c_ approaches zero. Some *I*_c_ data points below a few amperes were removed to reduce overlap of the lines at each field. These plots cover most of the useful range of temperature and magnetic field for the respective samples, which are limited by the transition temperature and upper critical magnetic field. The limited thermal stability of two samples, identified as Nb_3_Sn #2 and MgB_2_ in Table 1, limited the higher current measurements for these samples even though they were soldered to the Cu-Be spring of the VTS probe.

It is useful to compare the slope of *I*_c_(*T*) for all of the samples near 5 K since this is where we will be making *liquid*/*gas* comparisons. Figure 5 shows the slope of *I*_c_(*T*) in units of A/K versus *I*_c_ at 5 K for all four samples. For Nb_3_Sn wires *I*_c_(*H*, *T*) also changes significantly with applied strain [1], so data are shown for two different applied strains. A semi-logarithmic plot of the absolute value of the slope of *I*_c_(*T*) in units of %*I*_c_/K versus *I*_c_ at 5 K is shown in Fig. 6. The transition temperature is one parameter that determines the *I*_c_ sensitivity to *T*, which is why the Nb-Ti sample is more sensitive than the other two types of superconductors. The two Nb_3_Sn wires have nearly the same transition temperature, but Nb_3_Sn #2 has a higher critical-current density than Nb_3_Sn #1, which causes a bigger change in *I*_c_ with *T*. Because the transition temperature of MgB_2_ is about 39 K, the slope of *I*_c_(*T*) is much lower at all measured critical currents. HTS materials such as YBa_2_Cu_3_O_7-δ_ (YBCO) would have even lower slopes of *I*_c_(*T*) than MgB_2_ at 5 K. Because of these lower slopes and, in some cases, critical currents that vary more with time and magnetic field history, HTS samples are not as appropriate for making *liquid*/*gas* comparisons at 5 K.

For a given wire, the temperature sensitivity in units of %*I*_c_/K increases with decreasing *I*_c_, which means that temperature errors can be a bigger problem in the regions of low *I*_c_. The region where *I*_c_ is less than 5 A is typically not useful for most applications for wires with diameters larger than 0.5 mm. To illustrate these points, consider the change in *I*_c_ for the Nb-Ti sample when the 5 K temperature is changed by 0.05 K at different magnetic fields. At 5 T, where the *I*_c_ is 369 A, a 0.05 K change would change the *I*_c_ by about 7.9 A or 2.1 %. At 8 T, where the *I*_c_ is 97 A, a 0.05 K change would change the *I*_c_ by about 8.3 A or 8.6 %. At 9 T, where the *I*_c_ is 23 A, a 0.05 K change would change the *I*_c_ by about 5.5 A or 24 %.

The following estimates of uncertainty are based on critical current measurements on stable Nb-Ti and Nb_3_Sn wires with diameters from 0.5 mm to 0.85 mm. Due to our limited experience with MgB_2_ wires, we do not have an uncertainty estimate for this sample. A conservative estimate of the standard uncertainty of the Nb-Ti critical-current measurements with the sample in liquid helium due to systematic effects is 1 % +1 A, and that due to random effects is 0.2 % + 0.2 A. A conservative estimate of the standard uncertainty of the Nb_3_Sn critical-current measurements within the reversible strain region [10] and with the sample in liquid helium due to systematic effects is the larger of 2.5 % +2 A or the effect of changing the strain by 0.03 %, and that due to random effects is the larger of 0.6 % + 0.5 A or the effect of changing the strain by 0.01 %. For measurements done in flowing helium gas in the VTO probe, the additional critical-current uncertainty due to systematic effects is the equivalent of a temperature change of 0.03 K, and that due to random effects is the equivalent of a temperature change of 0.01 K. For measurements done in flowing helium gas in the VTS probe, the additional critical-current uncertainty due to systematic effects is the equivalent of a temperature change of 0.06 K, and that due to random effects is the equivalent of a temperature change of 0.02 K.

## 2. Experimental Details

### 2.1 Re-entrant Dewar and Control Loops

#### 2.1.1 Philosophy of Temperature Control in *Gas*

A schematic diagram of the lower part of our variable temperature measurement system is shown in Fig. 7. The sample probe is inserted into a re-entrant dewar (also known as a variable-temperature insert), which is inserted into a dewar with a superconducting magnet. Some of the system is built into the re-entrant dewar and it is used by multiple sample probes. This schematic is shown with the VTS probe. The differences between the VTO and VTS probes will be described later. The horizontal gaps between many components are exaggerated for clarity. The re-entrant dewar has a vacuum and super-insulated space with a liquid helium transfer tube that goes from the outside to the inside. The transfer tube is a thin-walled stainless-steel tube with an outer diameter of about 3.2 mm. It is coiled for one turn around the vacuum space of the re-entrant dewar, which accommodates thermal contraction and is easier to weld in place than a bellows feed-through that was used in an earlier design. The transfer tube allows liquid helium to flow from the magnet dewar to the middle of the re-entrant dewar.

The variable temperature system has four separate control loops that work together to provide the necessary cooling power for the sample and the sample current leads. There are many long time-constant interactions between the four control loops, and the main computer program needs to keep the system stable. The reason for the four control loops will not become apparent until we examine the protocol necessary to achieve the “correct” critical-current measurements under flowing helium gas conditions at high current levels. The four control loops and the protocol attempt to create a relatively uniform temperature over the length of the sample and a predictable relationship between the average thermometer temperature and the average sample temperature. We use many control loops to control pressure, gas flow, heater powers, and temperatures. Most of these control loops use the very common proportional-integral-derivative (PID) control algorithm.

To control temperature during our VTM we need (a) a cooling power that is provided by a flow of cold helium gas and (b) an electric heater with a controller that adjusts the heating power to obtain a constant temperature. If the heater power goes to zero, the temperature control is lost. The liquid-helium flow from the magnet dewar is determined by control loop #4. This helium flow is converted to gas and warmed somewhat by the pre-regulator, which is control loop #3. In order to exit the re-entrant dewar, this gas has to flow down the outside of the sample probe (outlined with two dark lines) and then up the inner part of the sample probe, over the bottom current contact and control loop #2 (heater power *H2*), over the sample coil, and finally to the top current contact and control loop #1 (heater power *H1*). From there, the gas continues up the inside of the sample probe and exits out the vapor-cooled current leads. This gas continues to intercept heat that is flowing down the sample probe and current leads as it exits the re-entrant dewar. Flow meters at room temperature are used to monitor this gas flow. One can consider that control loops #3 and #4 provide the coarse temperature control and control loops #1 and #2, which are downstream from #3 and #4, provide the fine temperature control.

In the schematic shown in Fig. 7, we show that some of the gas flow up the VTS probe bypasses control loop #2 (bottom of the sample) and re-enters the main gas flow near control loop #1 (top of the sample). This bypass flow is intended to provide more cooling power for control loop #1, which is otherwise only downstream from control loop #2. With both of these control loops having the same temperature set point, one would expect that the heating power necessary for loop #1 would be zero or very low without some independent cooling flow for the VTS probe. In contrast, the VTO probe was designed to have more even cooling of these two control loops, so the bypass flow was not necessary for this probe. The design trade-offs between these two probes will be described later.

#### 2.1.2 Coarse Temperature Control in *Gas*

Control loop #4 adjusts the pressure set point of the magnet dewar in order to ultimately control the total heater power of the other three control loops. The rate of helium flow is controlled by the impedance of a needle valve and the rest of the flow path, and the pressure difference between the two dewars. A “butterfly” valve with a PID pressure controller is located on the helium gas exhaust line of the magnet dewar to control the pressure. The pressure controller uses a PID algorithm to adjust the opening of the valve so that the dewar pressure is close to the set point. The difference between the dewar pressure and the pressure set point is the error signal for the valve PID. The main computer program has a target value for the total heater power (pre-regulator power plus sample heater powers *H1* and *H2*). This total heater power target depends on the expected maximum current, *Imax*, of the *V-I* curve. The program determines this power target by using an interpolation table of total heater power target values at various *Imax* values, which the user can easily modify in real time. Within the computer program we have a second control algorithm (not a PID) that operates to reduce the error signal that is the difference between the actual total heater power and the target. Depending on the magnitude and sign of this error signal, the pressure set point is changed unless the error is within some tolerance. So, control loop #4 needs two separate control algorithms. The computer program control algorithm needs to have special features to reduce the chance of oscillation with the other three control loops and not to over-correct when there is a high sample current that reduces the total heater power. This algorithm is customized to increment the set point by an amount proportional to the error every 20 s or so, but the absolute value of the increment is clipped so that there is never a big increment. If the sample current is on, the increment is clipped to an even smaller value even if the error signal becomes very large in order to limit windup of the controller that would cause overshooting when the current is brought back to zero.

An alternative set point selection for control loop #4 is the helium gas-flow rate out of the re-entrant dewar instead of the total heater power of the other three control loops. Manually switching to control the gas-flow rate is a better feedback choice when the sample temperature is high (>25 K) and the system thermal time constants are longer. At higher temperatures it takes longer for the heater powers to reach equilibrium than it does for the gas flow.

The typical magnet dewar differential pressure ranges from 2 kPa (15 torr) to 8 kPa (60 torr) in order to have enough liquid helium transferred from the magnet dewar to the re-entrant dewar. Because of the low atmospheric pressure at our test site, the magnet temperature is still well below 4.2 K so we can operate the superconducting magnet at its full-rated field. The other approach to forcing liquid helium to transfer between the dewars is to pump on the re-entrant dewar. This could be done by increasing the impedance of the flow control valve and transfer line; however, this would reduce the helium gas pressure in the re-entrant dewar to nearly a vacuum that would not have the desired effect of improving the cooling and temperature uniformity of the sample. It would be better to put a pressure controller or manostat between the pump and the re-entrant dewar so that the pressure of the helium gas flow is only slightly less than one atmosphere.

Control loop #3 adjusts the temperature set point of the pre-regulator in order to ultimately control the heater power of one of the sample heaters, either *H1* or *H2*. Typically, the power level of *H1* is controlled because it is the farthest along the path of the helium gas, and thus is likely to be closer to zero power. We want to operate with *H1* at 3 mW to 50 mW, depending on *Imax*, but we do not want to lose temperature control. The pre-regulator will typically operate between 0 W and 2 W. The pre-regulator and liquid flow needle valve are built into the re-entrant dewar. After the needle valve, the helium flows through four tubes that are soldered to a cylinder that fits around the sample probe. The cylinder provides a thermal coupling between the helium flow and the heat flowing down the probe, which will convert some of the liquid to gas. Half of the pre-regulator heater is also on this cylinder and the other half is on a liquid trap that has the control thermometer for loop #3 inside the tubing with the gas flow. The pre-regulator is strategically placed to use the probe’s steady-state heat load to boil some of the helium and to use as much of the heat generated during high-current testing as possible to heat the helium, replacing the heater power of the pre-regulator. After the liquid trap, the gas flows down to the outlet ring where there are a number of outlets that direct the gas down the tail of the re-entrant dewar on the outside of the probe and then, up the inside of the probe over control loop #2, the sample, and control loop #1.

Control loop #3 has two parts similar to that of control loop #4. The first part is a typical PID temperature controller that adjusts the heater power to control the pre-regulator temperature. The second part is a customized PID algorithm within the main computer program that typically monitors the heater power of control loop #1, *H1*. The target heater power for *H1* depends on the expected *Imax*. The program determines this target by using an interpolation table of *H1* target values at various *Imax* values, which the user can easily modify in real time. The difference between *H1* and the *H1* target value is the error signal for the customized PID algorithm, which slowly adjusts the pre-regulator temperature set point to attempt to reduce this error signal. It is important to notice that the pre-regulator temperature set point varies with time and is typically less than, but is close to the sample temperature. As with part of control loop #4, the custom algorithm in loop #3 has to take small steps to reduce the chance of oscillation and to limit windup when the sample current is on. Another unique problem is that when the *H1* target is low, for example 0.005 W, the largest-magnitude error signal below the target is −0.005 W, because the heater power cannot go below zero. This creates an asymmetric error signal around the target that can produce a loss of control and error signals too small to re-gain control. Several customized modifications were needed to attempt to address this unique problem; however, we make no claims about the effectiveness or general applicability of our solutions. One modification is to automatically multiply the negative error signal by 1, 2, or 4, depending on the original magnitude of the error signal. A multiplier of 1 is used if *H1* is within some tolerance of the target, which has the desired effect of not over-correcting. As *H1* approaches zero, factors of 2 and then 4 are used to make more aggressive changes to the pre-regulator temperature set point. Another custom modification to the algorithm is that the error in the temperature of loop #1 is scaled appropriately and added to the heater error. This has the effect of providing an appropriately increasing error signal as control loop #1 loses temperature control because its heater power, *H1*, has gone to zero. Both of these modifications help provide a more symmetric error signal around the target and make it more likely that the system will automatically settle near the target. These are some of the many custom features of the VTM system that were deemed necessary for better control of the complex system.

The computer-program parts of control loops #3 and #4 have manually adjustable limits and total manual operational modes. The manual modes are sometimes needed when changing sample temperature or when the sample temperature is high (>25 K) and the system thermal time constants are longer. A manual software limit is set for the magnet dewar pressure so that loop #4 does not exceed the expected range needed for control. A manual limit is set for the pre-regulator heater power to reduce potential oscillations in control loop #3, and if it is over the power limit, the pre-regulator temperature set point is reduced regardless of the error signal. Another case is when the pre-regulator heater power is below 0.01 W, the pre-regulator set point is increased regardless of the error signal. This keeps the pre-regulator near the temperature where it can provide heating when necessary.

The computer program is very complex and many routines take different actions depending upon many variables. In order to help the operator follow the program logic in real time, we created a “case list” that identifies and creates a list of the ten (rows) most recent cases (column 1) with an indicator of how many consecutive times each case occurred (column 2). A hypothetical pre-regulator case list example is shown in Fig. 8. The case list is a two column by 10 row table with the most recent case on top. Notice that if a new case occurs, it is listed at the top of the table and the count starts over again at one, even if the case was listed in an earlier row. There are separate case lists for each of the complex routines in the program and they are displayed in various tabs on the program front panel.

#### 2.1.3 Fine Temperature Control in *Gas*

Control loops #1 and #2 use conventional, PID temperature controllers with temperature set points that are fixed for a given magnetic field. The resistive thermometers in these control loops do have magnetoresistance and we calibrate each thermometer over a matrix of temperatures and magnetic fields. This calibration is detailed in the next section. The computer program uses these calibrations and also takes into account where the thermometers are located within the magnetic field profile. When the computer program sets a new magnetic field, it estimates the magnetoresistance correction for each thermometer and sends an appropriate temperature set point to each controller so that the desired target temperature is reached. Typically the two temperature targets, *T1* for loop #1 and *T2* for loop #2, are the same. However, in some cases with the VTS probe, *T1* is set 0.03 K (or more) higher than *T2* to accommodate the relative thermometer calibration errors (including magnetoresistance effects) between the two thermometers that may vary with temperature and magnetic field. The relative thermometer calibration errors are unavoidable and, if the actual *T1* is lower than the actual *T2*, it can be impossible to control *T1* in the VTS probe because loop #1 has mostly downstream cooling.

#### 2.1.4 Measurements in *Liquid* and Comparing *Liquid* to *Gas*

For measurements in *liquid*, our re-entrant dewar can be sealed, filled with liquid helium, pressurized, and the liquid heated to 5 K. Because the outside shell of the probe is fairly well sealed, another exit path for the gas is needed so that the gas pressure does not force the liquid helium up the inside of the probe. There is a port on the sample probe near the inlet to the vapor-cooled leads (above the view shown in Fig. 7) that can be manually opened to allow gas to exit the re-entrant dewar above the liquid level. It is important to deliver at least some heat to the liquid at the bottom of the dewar because cold helium is more dense and the temperature will stratify in the dewar. It is actually fairly difficult to heat helium to equilibrium temperature at 5 K without boiling all of the liquid away and without taking a long time to asymptotically approach equilibrium. We start by heating to a target temperature of about 5.04 K while holding the pressure (207 kPa, 1553 torr) at the equivalent of liquid helium at about 5.08 K. Having an initial target of 5.04 K helps reduce the asymptotic approach to equilibrium, and the higher-pressure target reduces the boil-off during the heating process. It takes an energy equivalent to 10 W for about 15 minutes to heat the liquid helium (7 to 10 liters) in the re-entrant dewar from 4.0 to 5.0 K. The total power starts at 10 W for about 10 minutes from the pre-regulator, *H1*, and *H2*, and then decreases towards zero over the next 30 to 40 minutes. Then, when the temperature is close to equilibrium at 5.04 K, we turn off the heaters and monitor the temperature drop over time. If the temperature drops below 5.02 K, the heaters are turned back on and we check again after a few minutes. If the temperature stays above 5.02 K, we drop the pressure to 194.5 kPa (1459 torr) to achieve equilibrium boiling near 5 K. Stratification is not a concern when the pressure is decreased. It typically takes about 1 hour to get the liquid helium into equilibrium at 5 K.

For both *liquid* and *gas* data, the same resistive thermometers are used to determine the sample temperature. Thus, the thermometer calibration errors, including errors in magnetoresistance, will be essentially cancelled in the *liquid*/*gas* comparisons at 5 K. When the thermometers are in liquid helium and in thermal dynamic equilibrium with the system pressure, we can compare the temperature readings of the thermometers with each other and with that determined from the system pressure. Typically, these comparisons are within 0.02 K.

For measurements in *gas*, the apparatus is capable of a wide range of helium gas flow rates, 0.008 g/s to 0.12 g/s (0.05 L/s to 0.7 L/s at STP), to provide the necessary cooling for testing currents between 1 A and 1000 A. We will systematically study how *I*_c gas_ will depend on the amount of heater power near the sample at 5 K and higher temperatures. Because of the placement of *H2* and the direction of the gas flow, *H2* is an indicator of the cooling power of the gas flow just before the gas flows over that sample. Higher *H2* will be correlated with more sub-cooling of the sample relative to the thermometers and a higher measured *I*_c gas_.

### 2.2 Thermometry

The accurate measurement of temperature in high magnetic fields has always been challenging. Capacitance thermometers are the least affected by magnetic field; however, they cannot be calibrated because they often shift between thermal cycles and they drift after each temperature change. Thus, they are used only to control the temperature while the magnetic field is changed. This allows them to be used to calibrate the magnetoresistance of resistive thermometers. Some resistive thermometers (metal oxy-nitride resistors) with a low magnetoresistance effect are available [11]; however, using the general literature values would lead to a temperature uncertainty of more than 0.1 K in our region of interest due just to magnetoresistance. Thus, we decided to calibrate our specific resistive thermometers to reduce the overall uncertainty and bias of the temperature measurement.

The magnetoresistance calibration was performed using a simple probe inside the same re-entrant dewar and pre-regulator as the VTM system. The probe had only one heater on an oxygen-free, high-conductivity (OFHC) copper block that had four resistive thermometers and two capacitance thermometers. The two capacitance thermometers were wired in parallel to give a larger signal. The pre-regulator was used to keep the thermometer block heater power low, typically less than 0.02 W, and thus reduce the temperature gradients within the block and between the thermometers. This low thermometer block-heater power is close to the lowest powers for *H1* and *H2* during *I*_c_ measurements.

We calibrated the magnetoresistance effect of our resistive thermometers for temperatures from 1.63 to 30 K and magnetic fields from 0 to 16 T. The experimental protocol for these measurements was to set the temperature in zero magnetic field by using a calibrated resistive thermometer, then switch to control using a capacitance thermometer, then ramp the magnetic field to 16 T, hold for 60 s at 16 T, and then return to zero field. The maximum ramp rate for our 16 T superconducting magnet is 1.28 T/s and somewhat slower at the higher fields, so it takes about 30 minutes to complete the ramp up and down. Curves at 5 K and below were taken in liquid helium where the temperature was controlled by controlling the system pressure. Where possible, there was good agreement between the magnetoresistance determined using the capacitance thermometer and the temperature determined by using the equilibrium pressure of the liquid helium. Data were recorded on all thermometers throughout this process. This protocol removed the shifting problem that capacitance thermometers have with thermal cycling and allowed for an approximate correction for the slight drifting problem inherent to capacitance thermometers. The initial temperature was held constant for 30 minutes or longer to reduce drift during the field cycle. Holding the field at 16 T and comparing the calibration of the ramp up and down segments verified that there were no significant field-ramping effects. Calibrated resistive thermometers allowed future variable-temperature measurements to be performed without the special protocol needed to accommodate the shifting and drifting problems of capacitance thermometers.

The magnetoresistance effect for one resistance thermometer is shown in Fig. 9. The *x*-axis is the magnetic field and the *y*-axis is the apparent change in temperature, Δ*T*, due to magnetoresistance. Each of the ten curves on Figs. 9(a) and 9(b) were taken at various temperatures. There are more than 2000 readings of temperature along each curve and symbols are plotted only every 100 readings. In some cases, a slight temperature (a few millikelvins) hysteresis can be observed between the increasing and decreasing field segments of the curves. If the Δ*T* is positive, the apparent temperature is higher than the real temperature. The Δ*T* at a given field varies slowly with temperature. It is most positive at 1.63 K and 16 T and most negative at 8 K and 16 T. Above 8 K, the Δ*T* increases with temperature and is again positive at 30 K and 16 T. These data were divided into two plots to reduce the overlap of the curves. Curves at temperatures of 1.7, 4.5, and 11 K were omitted from Fig. 9 for clarity. Linear interpolation between curves at adjacent temperatures can be used for intermediate temperatures. A forth-order polynomial was used to fit the apparent change in temperature versus magnetic field. These curves were used to correct the measured temperature for each thermometer during the *I*_c_ measurements.

When we purchase thermometers for a given probe, we have them selected from the same batch with nearly the same resistance (within 10 %) at 4 K. With this selection, we have found that the magnetoresistance effects are nearly identical, which reduces systematic effects. The family of curves shown in Fig. 9 is very typical for the thermometers we have characterized. Figure 10 shows the Δ*T* at one magnetic field, 12 T, versus temperature for 11 different thermometers. The two thermometers illustrated with the first two symbols in the legend (solid circle and solid square) were from our first batch of thermometers. The three batches of three thermometers that we have procured since then have very similar magnetoresistance effects with temperature and with magnetic field.

Once we have the approximate Δ*T* (*H*, *T*) for a given resistive thermometer, we can use it to control the temperature during the field sweep of the magnetoresistance calibration rather than using the capacitive thermometer for control. A resistive thermometer for temperature control has the advantage of higher sensitivity to temperature, which leads to better control than with a capacitive sensor. The capacitive sensor is still recorded and used to estimate the temperature during the field sweep. The computer program monitors the magnetic field and changes the temperature set point of the resistive thermometer appropriately as the field sweeps. The program uses the current value of the magnetic field and the measured field ramp rate to forecast the field and the appropriate temperature set point 6 s into the future. A new set point is sent to the controller if the appropriate temperature has changed by more than 2 mK or it has been more than 3 s since the last change in set point. These two conditions for changing the set point keep the program from constantly updating and over-running the controller, but allow the temperature to track the field sweep.

### 2.3 Variable-Temperature Only (VTO) Probe

The sample is mounted on a thin-walled, coil mandrel with OFHC copper current-contact lugs on each end, as shown in Fig. 11. The coil mandrel can be made of either a non-magnetic stainless steel or an oxidized Ti-6Al-4V (percent by mass, Ti-6-4, also known as Grade 5 Titanium) alloy. If the mandrel is stainless steel, we have the option of soldering the sample to the mandrel with non-superconducting solder to keep the specimen from moving and to provide some extra thermal stability. For both mandrel types, the mandrel diameter is about 32 mm with a 90° spiral groove with a pitch of 3.2 mm and 3 turns between the current contacts. Two bolts clamp the current lugs to the coil mandrel. The bolts are insulated from one lug so that they do not provide a parallel electrical path for the sample current. The *I*_c_ measured when the sample is normal is less than 0.03 A, indicating that the shunted current in the mandrel and solder is quite low. Three twisted pairs of voltage-tap wires are soldered along the sample to provide the main sample *V-I* characteristics. Each of the main sample voltage-tap pairs measure adjacent, 8-cm segments of the sample. Additional pairs of voltage taps are placed along the entire current path to allowing monitoring of the voltage drop along all portions of the sample and current leads within the probe. The current bus bars are connected to each copper lug so that the sample can be soldered to this sample test fixture separate from the VTO probe. The bus bar that delivers current to the right (bottom when in use) lug goes through and is insulated from the left lug. This bottom lead has an extra joint (extra resistance) as it transitions to the surface of the right lug. The test fixture can be bolted into the probe. The current contacts are made between the probe’s current leads and the sample current bus bars at a distance selected so that the test fixture does not need to be heated again.

The lower part of the VTO probe is shown in Fig. 12 with the test fixture bolted between the top and bottom terminals. In operation, the left direction is up (see arrow on left end of figure). There are slots (not shown) in the terminals into which the current bus bar slips. The top and bottom terminals each have a thermometer and heater. The two heaters are aluminum-backed, low-inductance, Kapton-foil heaters with a resistance of about 29.5 Ω and dimensions of 0.27 mm × 32 mm × 57 mm. The heaters are fastened to the terminals with epoxy. We refer to the temperatures and heater powers as *T1* and *H1*, and *T2* and *H2* for the top and bottom, respectively. The top and bottom copper shells are two halves of a cylinder. The top copper shell is greased (with special high-thermal conductive, cryogenic grease) and screwed (four screws) to the top terminal so that they are in electrical and thermal contact. The lower part of the top shell is screwed (two screws through two slotted holes) to two fiber-glass epoxy insulators that are connected to the bottom terminal. The bottom copper shell is connected to the terminals in the opposite manner. Follow the assembly arrows from the shells to the terminals to see how the two shells fit around the test fixture and terminals. There is a gap between the two shells so that they are not electrically connected. Polyester electrical tape is used to seal the gaps so that the gas must flow down the outside (to the right) of the two shells and then up the inside over the bottom terminal, the sample, and top terminal.

The two copper shells provide more thermal mass and cooling surface to each terminal. But, more importantly, they provide more symmetric cooling for the two current contacts since the gas flows over these shells in a more symmetric manner than it does over the two terminals and current lugs. The gas that flows over the outside of the top shell provides some cooling for the top terminal before that gas flows over the bottom heater. This provides some “up-stream” cooling for the top of the sample. The shells also provide an isothermal shield for the sample. There is an insulating spacer below the bottom terminal made of fiberglass-epoxy. The spacer prevents the tail of the probe from shorting to the inside wall of the re-entrant dewar. The spacer has some flat segments around its circumference to allow gas to flow past it. There are holes through the spacer to allow liquid to drain out or gas to flow through. Gas can also flow through a 1-mm gap between the bottom of the shells and the top of the spacer. There are flat regions, smaller diameter sections, and slots in both terminals to allow gas to flow up the inside of the shells and over the sample. Only certain portions of the terminals have the same outer diameter as the inner diameter of the shells, and these portions are where the shells are greased and screwed to the terminals. The outer diameter of the Cu lugs and the sample mandrel is less than the inner diameter of the shells, with enough gap for the sample wire and solder.

The VTO probe was the first coil-sample geometry probe we designed to fit into a re-entrant dewar that fits into a 52-mm bore magnet. We have made measurements to temperatures as high as 90 K with the VTO probe and up to 65 K with the VTS probe (introduced in the next section). There is no reason that the VTS probe could not be operated at a temperature as high as the VTO probe. The VTO probe was also a prototype for the VTS probe. Our previous coil-geometry VTM system was designed to fit into an 86-mm bore magnet, which allowed more space around the sample. The advantage of the smaller bore magnet is the higher magnetic field capability that is important for more complete characterization of Nb_3_Sn samples. The challenge is fitting and electrically and mechanically connecting all of the components in the sample region, and having a less thermal mass near the sample. Most of the design of the VTO probe, other than the region around the sample, was duplicated in the VTS probe. The prototype probe allowed us to verify some of the new and smaller design components and visualize the structure needed to be compatible with the variable strain components.

### 2.4 Variable-Temperature and Variable-Strain (VTS) Probe

The variable-temperature and variable-strain (VTS) probe presented here was designed based on the combination of a low-current variable-temperature and variable-strain “Durham” probe [12] and the above introduced high-current VTO system. A Walters spring device [13] is used to apply a variable longitudinal strain (*ε*) to the sample. The spring is made from Cu-Be and has a T-section design with a wide elastic strain range between −1 % and +1 %. A worm gear is used to torque the top of the spring while the bottom of the spring is held fixed. The angular deflection of the spring (which has an operating range of about ±55°, corresponding to a sample longitudinal strain of about ±1 %) is read by use of a protractor and a pointer that are directly attached to the top and bottom of the spring, respectively. The protractor and pointer are inside a sealed space on the top of the cryostat that stays at room temperature. The angular deflection of the spring is read through a window in this sealed space. Strain applied to the sample is calibrated against the angular deflection of the spring. To read the angular deflection of the spring, to torque the top of the spring, and to hold the bottom of the spring, requires four concentric, mechanical connections to the sample test fixture. These four mechanical connections occupy space around the already complex and crowded region near the test sample.

Adding the variable strain feature to the VTO probe forced compromises: a smaller diameter working space, reduced thermal mass and reduced cooling surfaces of the current contacts, a longer sample length between the current contacts, larger distances between thermometers and the sample, and an off-balance of cooling power to each contact. In addition, we needed to be able to rotate one of the current leads by a total of about 110° as the Walters spring is rotated to deliver ±1 % strain to the sample. These compromises increased the challenges for making high-current VTM and required additional hardware and software changes. However, we maintained the ability to operate both the VTO and VTS probes in the same re-entrant dewar and pre-regulator, and with the same software.

The Cu-Be spring, OFHC Cu current contacts (Cu lugs), and current bus bar form a modular sample test fixture shown in Fig. 13. The Cu lugs are mechanically held to the Cu-Be spring with sets of pins, screws, and solder. The spring is 7.2 cm tall and 2.5 cm in diameter with 4 active turns in the center. Each end of the coil sample laps onto each lug and is soldered along its entire length. We typically add another coil sample segment in parallel with each end of the sample, outside the active portion of the spring. These extra coil segments act as splices around any potentially damaged portions of the sample ends and help carry the current to the active portion of the sample. Only the T-section of the spring is electrically and mechanically in parallel with the sample in the active region of the spring. The *I*_c_ measured when the sample is normal is less than 0.03 A, indicating that the shunted current in the spring and solder is quite low. The sample test fixture is soldered and instrumented before it is installed on the apparatus. The current bus bars are made of Cu strip and a number of layers of YBCO-coated conductors. The bus bars are imbedded in the Cu lugs and the current transfers from the YBCO to the helical sample over the length of the Cu lugs. The portion of the bus bar that extends past the Cu lug allows for the final current connection to be made between the current bus bars and bus bars in the sample probe. Two set screws for the tube that is connected to the protractor can be seen on the left end of the spring in Fig. 13. Two set screws for the rod that is connected to the pointer can be seen on the right end of the spring. The protractor tube and the pointer rod are not shown in Fig. 7, but they are inside the inner torque shaft that is shown. The protractor tube and the pointer rod each have an electrical isolation break along their lengths.

Two views (the probe is rotated about 180° between views) of the lower part of the VTS probe are shown in Fig. 14. The test fixture is bolted and pinned between the top and bottom Cu terminals. In operation, the left direction is up. The top terminal is pinned and screwed to the central torque shaft that is connected to the worm wheel at the top of the sample probe. Each terminal has a thermometer and heater. The two heaters are aluminum-backed, low-inductance, Kapton-foil heaters with a resistance of about 28.4 Ω and dimensions of 0.27 mm × 32 mm × 32 mm. The heaters are fastened to the terminals with epoxy. A current lead can be seen in the lower view as it attaches to the top current bus bar in a slot in the top terminal. The soldered current connection is made in the slot without significant heat transfer to the terminal or lug. A Cu cylinder with a current lead (not shown in Fig. 14, but shown in Fig. 7) slips over the lower part of the probe and is soldered to the bottom current bus bar in a slot in the bottom terminal (see upper view). The pins that stick out the bottom of the bottom terminal slip into a stainless-steel torque can (not shown in Fig. 14, but shown in Fig. 7) that slips over the lower part of the probe and couples the torque between the central shaft and the outer structure of the probe. A fiberglass-epoxy disk attaches to the bottom of the stainless-steel can that prevents the tail of the probe from shorting to the inside wall of the re-entrant dewar. Another fiberglass-epoxy disk electrically isolates the top of the stainless-steel can from the upper part of the sample probe as shown in Fig. 7. The heads of nylon screws in the top terminal prevent the Cu cylinder and bottom current lead from shorting to the top of the sample.

Because the top terminal and current lug rotate a total of about 110° relative to the bottom terminal and current lug, we could not split Cu shells in the VTS probe like we did in the VTO probe. In addition, the outer stainless-steel can would cover any Cu shells, which would defeat the purpose of trying to provide more symmetric cooling and cooling surface for each current contact.

The current lead that carries current to the top of the sample is mounted on, and travels with, the lower part of the inner torque shaft and rotates a total of about 110°. This current lead transitions to a fixed, vapor-cooled lead about half way up the inner torque shaft. The inner torque shaft has an electrical break above this point. The transition from the rotating lead to the fixed, vapor-cooled lead is accomplished by a spiral of copper foils and superconductors.

The stainless-steel can has holes through the bottom to allow gas to enter the can from the bottom between the outer diameter of the bottom terminal and the inside diameter of the can, as shown by the gas flow arrows in Fig. 7. Once the gas is inside the can it has two paths: one outside and one inside the Cu tube/current lead. An insulating tube of fiberglass-epoxy covers the outside of the Cu tube so that the gas flow outside is not heated as much by the bottom heater. The outside gas flow path has to go through the holes in the Cu tube near the top terminal and lug because of the gas block, shown in Fig. 7, between the Cu tube and the inside of the stainless-steel can that is located above the top terminal. This outside gas flow path provides some cooling for the top of the sample that is somewhat decoupled from the bottom heater. Another insulating tube of fiberglass-epoxy fits inside the Cu tube to add some impedance for the inside gas flow to help balance the flow between these two paths. We expect that most of the gas flow is still up the inside of the Cu tube based on the relative impedances of the two paths.

Three twisted pairs of voltage-tap wires are soldered along the sample to provide the main sample *V-I* characteristics. Each of the main sample voltage-tap pairs measure adjacent, 8-cm segments of the sample, which is about one turn on the spring. Additional pairs of voltage taps are placed along the entire current path to allow monitoring of the voltage drop along all portions of the sample and current leads within the probe.

### 2.5 Data Acquisition

The *I*_c_ is determined from the measured *V-I* curve. The current signal and three sample voltages are measured simultaneously with four voltmeters. Figure 15 shows the current and one sample voltage (tap *V1*) versus time, *t*, for a Nb-Ti wire at 3 T and 5 K with the sample in liquid helium. There are about 4,100 readings of each the two signals in Fig. 15. Each of the four signals is recorded at a rate of about 50 readings per second with a one power-line cycle (60 Hz) integration period. These four voltmeters are on a communication bus separate from all of the other instruments and data are acquired by an independent computer program that shares its data with the main program using a common buffer system. This allows the optimal reading rate for the *V-I* curve acquisition regardless of what the main program is doing or which instruments it is communicating with. The main program has a matrix of estimated maximum current values, *Imax*, for various combinations of magnetic field and temperature. This program also has a maximum target sample voltage, *Vmax*, which is estimated based on *Imax*, the stability of the sample, and whether the sample is in liquid or gaseous helium. We interpolate values of *Vmax* at various *Imax* values by using a lookup table, which can easily be modified during the data run. The purpose of *Vmax* is to estimate how high the sample voltage can reasonably be and still avoid a thermal runaway situation that leads to a sample quench. We tend to conservatively estimate *Vmax* to reduce the number of sample quenches. Another programming parameter is *Np*, which is an estimated *n*-value that is estimated assuming it only depends on *Imax*. Again, we interpolate values of *Np* at various *Imax* values using a lookup table.

The main program controls and monitors the current and adjusts the ramp rate depending on the current and *Imax*. In Fig. 15, the three ramp rates are about 225, 126, and 75 A/s. The set of three ramp rates that are selected depends on the stability of the sample. If *Imax* is less than 200 A, then only the slowest ramp (finishing) rate is used. If *Imax* is between 200 A and 400 A, then the middle rate is used below 70 % of *Imax* and the slowest rate is used above that. If *Imax* is above 400 A, then the fastest rate is used below 50 % of *Imax*, the middle rate is used between 50 % and 70 % of *Imax*, and the slowest (finishing) rate is used above that. It is difficult to see the different slopes of *I*(*t*), but the inductive voltages of *V*(*t*) are more evident. Even though the current ramp rates are constant for each segment, the variable inductance of the voltage taps [14] causes the voltage to vary over each ramp segment. The inductive voltages are more constant over the downward ramp segments.

The parameter *Np* is used by the main program to determine how much to change the current during different parts of the *V-I* curve acquisition. The “*p*” in *Np* refers to the word “pattern.” *Np* determines the pattern of current steps in the *V-I* curve. We typically want the sample voltage points along the curve to be equally spaced on a logarithmic scale with about 6 points for every decade of voltage (example *V*: 0.1, 0.15, 0.22, 0.32, 0.46, 0.68, and 1.0). Then, given *Np*, the spacing of the corresponding currents can be estimated. If the *Np* = 20 and *Imax* =1000 A, some current set points would be 891, 909, 926, 944, 962, 981, and 1000 A. The desired step size is 1.9 % (about 19 A). If the *Np* = 57 and *Imax* = 1000 A, some current set points would be 960, 967, 973, 980, 987, 993, and 1000 A. The desired step size or quantum is about 0.67 % (~6.7 A). A higher *Np* means that the voltage will be increasing very quickly with current as *I*_c_ is approached, so the current needs to be incremented in smaller steps and the current quantum sets this scale.

*Imax*, *Vmax*, and *Np* are used by the main program during the first part of the acquisition to control the current and limit the sample voltage. We call this part of the acquisition the *FIND*, where the program hunts for the current that will result in a sample voltage near *Vmax*. In Fig. 15, the *FIND* occurs between 3 and 15 s of the waveform and the approximate parameters are *Imax* = 555 A, *Vmax* = 4 μV, *Np* = 57, and one current quantum is 3.7 A. The current waveform starts and ends at zero to allow for sample voltmeter offset and drift corrections. After the initial zero current, the current is ramped to the first set point that is lower than the *Imax* by a programmable undershoot value (typically 20 A) and six quanta of current (equivalent to one decade of voltage). During the *FIND* portion, the current is held for 0.5 s and the voltages recorded. Depending on how close voltage is to the target, the current is stepped by some multiple of the quantum, but never more than 5 quanta or less 0.25 quantum. The voltage is evaluated 0.5 s after each step and the number of quanta for the next step is conservatively estimated to approach the target from below without overshooting. If the voltage oscillates around the target, then the minimum step is reduced to 0.25 quantum. In Fig. 15, the *FIND* used the following sequence of quanta: 3, 2, 1, 1, 1, 0.5, 0.5, 0.5, 0.5, and 0.5. This process of stepping the current and evaluating the voltage continues with appropriately smaller steps until the voltage is within certain limits of *Vmax* and the *FIND* portion of the curve acquisition is completed. None of the measurements during the *FIND* portion are used to determine the *V-I* curve because the settling time periods are too short and in the case of measurements in *gas*, the heaters and temperature are still in a transient state.

The temperature of the two thermometers increases 5 or 6 mK and stabilizes by the time the *FIND* is complete in Fig. 15. Each thermometer is measured at a rate of about 2.5 readings per second. This slight temperature rise is due to the increased pressure in the re-entrant dewar that is caused by the increase in liquid helium boil-off and the resulting gas flow as the current is increased to 555 A. A manostat is used to control the pressure of the dewar when liquid helium is used and it has no remote pressure sense, making it somewhat sensitive to changes in gas flow rate. However, these changes in temperature are minor and easily measured by the thermometers.

A settling period of 6 s is used at the highest current and 3 s is used for each current step after that. Thirty-one readings (covering about 0.6 s) of current and each sample voltage are averaged after each settling time period and only these average values are used as the measured points along the *V-I* curve. From the highest current, the current is stepped down by 1 quantum increments. The current continues to be stepped down until the voltage is below some target value then the current is set to zero. This type of acquisition is backwards in some sense because the *V-I* curve is taken from high current to low current. We can acquire data with increasing current, but this requires that we separate the *FIND* and *V-I* curve with a ramp back to zero current, a settling time period, and then start the ramp back up. This takes longer and, especially at high current with the sample in *gas*, there is a transient in the heater and temperature when the current comes on. Another disadvantage of acquiring the *V-I* curve from low to high current is that if the sample quenches near the end of the curve, one loses more time. Moreover, the inductive voltages decay faster with time after the downward ramp segments compared with the upward ramp segments. We have compared results of different acquisition patterns with the sample in *liquid* (the most repeatable condition) and have observed only slight systematic differences (less than a few 0.1 % in *I*_c_) between forwards and backwards acquisitions. This one of the many trade-offs that needs to be made for VTM.

A full-logarithmic scale plot of *V-I* curves is shown in Fig. 16 for three voltage taps at two temperatures. These curves are fairly linear over small regions indicating an approximately constant *n*-value. The *V-I* curves below 0.1 μV can be influenced by voltage noise and other subtle effects that create interfering voltage [3] including negative voltages [15]. The *V-I* curves are fairly stable to voltages of 3 to 4 μV at these currents with the sample in liquid helium. We use a linear fit of three *V-I* points to determine *I*_c_ at a given criterion. For example, the voltage equivalent to *E*_c_ = 0.1 μV/cm is 0.8 μV (8 cm tap spacing), which is indicated by the horizontal reference line in Fig. 16. *I*_c_ values at 5 K for each of the three taps, *V1*, *V2*, and *V3* are 534.6 A, 536.2 A, and 538.6 A, respectively. The *n*-values near 0.1 μV/cm for the three taps are 53.8, 54.0, and 57.0, respectively. The slight *I*_c_ differences among the taps (a range of about 0.75 %) are very systematic as shown by the relative differences at 4.8 K being the same as at 5 K to within about 0.01 %. In some cases over an extended range of temperatures or magnetic fields, the relative order of the three sample segments can change in a smooth, continuous manner. Tap *V1* covers the centermost portion of the sample and had the lowest *I*_c_ in this case, but did not seem to be damaged, as suggested by the *n*-value and low range of *I*_c_ values. So, we will just focus on *I*_c_ for tap *V1*.

A superconductor near its *I*_c_ is an ultra-sensitive thermometer as illustrated in Fig. 16, where the voltage increases by a factor of over 30 at 550 A when the *T* is increased by 0.2 K (4.8 K to 5 K). The extreme sensitivity of the sample voltage to temperature is also evident in Fig. 15 at the highest voltage (around 20 s on the *x*-axis) where the sample voltage drifts down with time even though the current is constant and the sample is in *liquid*. Sample *T* changes (to lower *T* in this case) as small as a few millikelvins would account for this slight downward drift in voltage. In this part of the acquisition, the sample temperature is settling back down after the current was ramped up. This voltage drift is less evident at the lower voltage set points that are farther from the time of the current ramp. This type of sample voltage drift can be more significant for high-current measurements made in *gas*. The self-heating of the sample will also change significantly when the current is near *I*_c_ because the voltage drop changes quickly with current in this region. This change in self-heating can create systematic temperature changes with current that will tend to increase the measured *n*-value. The extreme sensitivity to *T* makes it difficult or impossible to accurately determine the *n*-value at high currents, especially for measurements made in *gas*. Thus, we will focus on getting the correct *I*_c_, not on getting the correct *n*-value in VTM.

## 3. Verification of Variable Temperature Measurements

### 3.1 Verification Approach

We do not present thermodynamic models of the sample temperature relative to the temperatures of the thermometers. This is considered too difficult to completely model. In addition, many parameters depend on *I*_c_ and *T*. For example, the total heater power targets prior to the *V-I* curve change with *I*_c_, which also means that the gas-flow rate changes. As a function of *T*, the gas-flow rate at a given total heater power, when the current is zero, decreases with increasing *T* because a larger fraction of the power is needed to heat the gas to a higher *T* (heat capacity) compared to most of the heater power providing the latent heat of vaporization when *T* = 5 K. The heat capacity of helium gas decreases slightly with increasing *T* from 4.2 to 30 K and then becomes almost constant for higher temperatures. In contrast, the heat capacity of most metals increases quickly with *T* and the approximate relationships are proportional to *T*^3^. The thermal conductivity of helium gas increases slowly with *T*, and the relationship is approximately proportional to *T*^0.6^.

Our approach is empirical. We will gradually make the case for *H2* and *T2* being key parts of the correct protocol for VTM and by implication, that *H1* and *T1* are less influential. We do not have space to present all of the observations that support this claim. The general premise of our VTM system with a high-flow rate of helium gas and a pre-regulator is that for low *I*_c_, ideally *H1* and *H2* should be zero to get the correct *I*_c_ at any *T*. Here the limit of low *I*_c_ is determined by the contact resistance and sample self-heating, which in our case is about 150 A. As *I*_c_ increases, the correct *H2* will increase to provide the needed additional cooling. The contact heating and any other heat flowing down the leads will decrease *H1* and *H2* as the sample *I* comes on, but *H2* during the *V-I* curve needs to be finite, indicating that the gas has enough cooling power to balance the self-heating of the sample. One could argue that all of these empirical determinations of protocol are relevant only to our particular VTM system, but we will make the case that some of what we determined with careful *liquid*/*gas* comparisons has implications for most, if not all, VTM systems. The main implication is that high heater power near the sample can create *T* gradients that can lead to significant *T* differences between the sample and the thermometer.

### 3.2 Verification Measurements with the Variable-Temperature Only (VTO) Probe

#### Liquid/gas comparisons on a Nb-Ti sample mounted on Ti-6-4 mandrel, but not soldered to mandrel

Figures 15 and 16 (*liquid*) and the next 11 figures contain data that were taken using the VTO probe on the same tap *V1* (except for Fig. 16 that shows all three taps), the same Nb-Ti wire mounted on a Ti-6-4 coil mandrel, and the sample was not soldered to the mandrel. We focus on this one set of data to provide the reader with a more complete comparison of *liquid* and *gas* measurements. We will start with temporal plots like Fig. 15, then look at *V-I* curves like Fig. 16, and finally compare *I*_c_ values. The next 11 plots, Figs. 17–27, will show that the thermometer readings, sample temperature, sample voltage, and *V-I* curves in *gas* are well defined and the resulting *I*_c_ values are not merely a product of chance, data selection, or clever averaging or fitting methods.

Figure 17 shows the current and one sample voltage (tap *V1*) versus time for a Nb-Ti wire at 3 T and 5 K with the sample in flowing helium gas. There are about 3,000 readings of each the two signals. This figure is comparable to Fig. 15. The only difference is that these data were taken with the sample immersed in liquid helium (*liquid*) in Fig. 15 and in flowing helium gas (*gas*) in Fig. 17. We try to compare *liquid* and *gas* measurements made on the same day to reduce the chance for small changes to the sample or the probe during the comparison. When we measure in *gas* with high-current, we cannot achieve the higher voltages on the *V-I* curve as when we measure in *liquid*; thus the target *Vmax* is lower and the curve takes less time to complete. In Fig. 17, the three current steps from about 22 s to 32 s are where the current is near *I*_c_ (*V* near 0.8 μV) and the averaged *V* and *I* readings near the end of each of these three steps are used to determine *I*_c_ at 0.1 μV/cm. The average readings of temperatures and heater powers over the same time period as these three *V-I* curve points are the most relevant for the measurement and we define these average readings as “near *I*_c_.” The average readings prior to when each *V-I* curve was acquired (0 s to about 5 s in the figure, where the current is still zero) are also important and we define these averages as “before curve.”

Figure 18 shows sample temperature *T1* and *T2* data taken during the same curve as shown in Fig. 17 with a maximum current of about 550 A. *T1* and *T2* are each read at a rate of about 2.5 readings per second. All of the readings are plotted; however, the symbols are plotted only for every 10^th^ point to identify the curves. The sample temperatures increase with current during the *FIND* portion of the curve because the heating from the current is increasing faster than the temperature controllers can decrease their heater powers. Then the temperatures returns closer to the target temperature 5 K. The three heater powers, *Pre-Reg*, *H1*, and *H2* versus time are shown on Fig. 19. The heater power readings are 1 s averages and symbols are plotted only for every 5^th^ point. The *Pre-Reg* (left axis) is about 1.6 W before the curve and drops to less than 0.1 W when the sample current is near its maximum. The *Pre-Reg* power returns to about 0.5 W during the latter half of the curve and jumps up over 1.7 W when the current is ramped to zero at the end of the curve. As can be seen by the large drop (about 1.6 W) in *Pre-Reg* power, the *Pre-Reg* intercepts much of the additional heat generated by the large sample current through the leads and soldered contacts. The starting *Pre-Reg* power can be thought of in two ways: it represents a dynamic reserve of cooling power that can easily be accessed to offset the heating from the sample current, or the starting *Pre-Reg* power can be replaced in whole or in part by the heating from the sample current. Most of the drop in *Pre-Reg* power is due to the heating from the sample current rather than the lowering of the *Pre-Reg* temperature set-point because *H1* is below its target. Sample heater powers *H1* and *H2* (right axis) start at about 0.08 W, dip down slightly (less than 0.05 W), but do not go to zero when the current comes on, and then increases when the current is ramped to zero. Figures 18 and 19 illustrate the typical dynamic response of heater powers and temperatures during a high-current (up to about 550 A in this case) *V-I* curve.

Figure 20 shows the average *Pre-Reg* power just before each *V-I* curve was acquired (solid circles) and the average power during the time when the current is near *I*_c_ (open circles) as a function of *I*_c_ for a Nb-Ti sample at 5 K in *gas*. We varied the applied magnetic field to increase *I*_c_ from about 5 A at 9.5 T to 750 A at 1.5 T. The minimum target *Pre-Reg* is about 0.5 W, which allows the *Pre-Reg* to have enough reserve power to control *H1* and, because *H2* is typically greater than *H1*, this keeps *H2* above zero and also in temperature control. For *I*_c_ below 100 A, the two *Pre-Reg* powers are nearly the same. As *I*_c_ increases above 100 A, the change in *Pre-Reg* power increases to more than 1.6 W. The target *Pre-Reg* power is increased with the expected *I*_c_ in order to intercept the heat generated by the higher currents. The heat that is not intercepted by the *Pre-Reg* will raise the temperature of portions of the probe, potentially including the sample, if this heat cannot simply replace the sample temperature controller’s heater power. In some cases the *Pre-Reg* power goes to zero during the *V-I* curve, which, although it is not desirable, does not necessarily mean that sample temperature control will be lost. If the *Pre-Reg* power had been slightly higher before the *V-I* curve was acquired, then this power might not have gone to zero during the curve.

Figure 21 shows the average *H1* and *H2* powers just before each *V-I* curve was acquired (solid symbols) and the average powers during the time when the current is near *I*_c_ (open symbols) as a function of *I*_c_ for a Nb-Ti sample at 5 K in *gas*. These measurements were made for the same curves as shown in Fig. 20. The target *H1* power is about 0.005 W for low *I*_c_ and about 0.16 W for *I*_c_ of 750 A. These targets are set based on the experience of how much the power drops during the *V-I* curve and the desired power levels when the current is near *I*_c_. *H2* is typically a little greater than *H1* prior to and during the *V-I* curve. The somewhat higher *H2* at the lowest *I*_c_ is likely due to the system not being completely settled when we start acquiring this set of data. Even when the *Pre-Reg* power went to zero, *H1* and *H2* stayed above zero and in temperature control.

To compare *liquid* and *gas* measurements at various *I*_c_ values, we first measure *I*_c_ as a function of magnetic field at 5 K and 4.8 K in *liquid* to give us the correct temperature dependence of *I*_c_. Then we switch to *gas* measurements at the same set of magnetic fields at 5 K. Figure 22 shows some *liquid* and *gas V-I* curves at 3 T. The *liquid V-I* curves at 4.8 and 5 K show the effect of temperature. Part of the *liquid* curve at 4.8 K is covered by the legend, but one can see the entire curve in Fig. 16, tap *V1*. The *H2* labeled for each curve is now just the average value when *I* is near *I*_c_, not *H2* prior to the *V-I* curve. The figure has a horizontal line at 0.8 μV to indicate the location of *I*_c_ at an *E*_c_ = 0.1 μV/cm. For the *gas* measurements, we expect that *I*_c gas_ will depend on the amount of heater power. Higher heater power near the sample was correlated with more sub-cooling of the sample relative to the thermometers and a higher measured *I*_c gas_. So we conducted a systematic study of measured *I*_c gas_ with different heater powers. Figure 22 illustrates that the *gas V-I* curves, and thus *I*_c gas_, shift with heater power. *H2* powers are listed in the legend for *gas* curves. The *n*-values for the *gas* curves are higher than for the *liquid* curves, which is likely due to slight changes (a few millikelvins) in sample temperature with current due to self-heating. However, the main feature is that *I*_c gas_ increases with *H2*, which is equivalent to a *T* decrease of 0.076 K (76 mK) for the curves shown here.

**Fig. 2:**
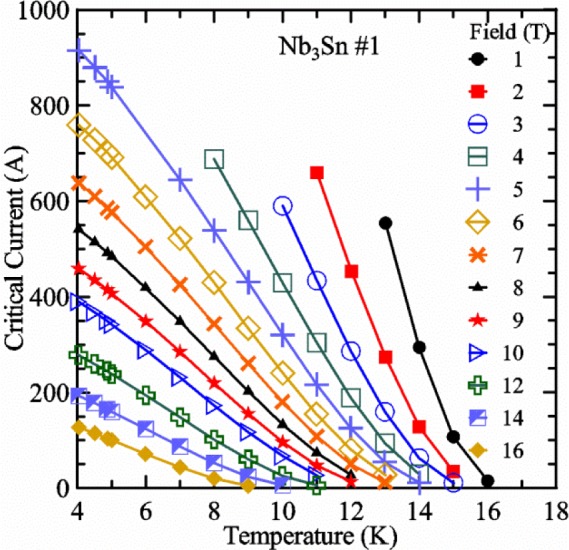
Critical current versus temperature at various magnetic fields for 0.818 mm diameter Nb_3_Sn sample #1.

**Fig. 5:**
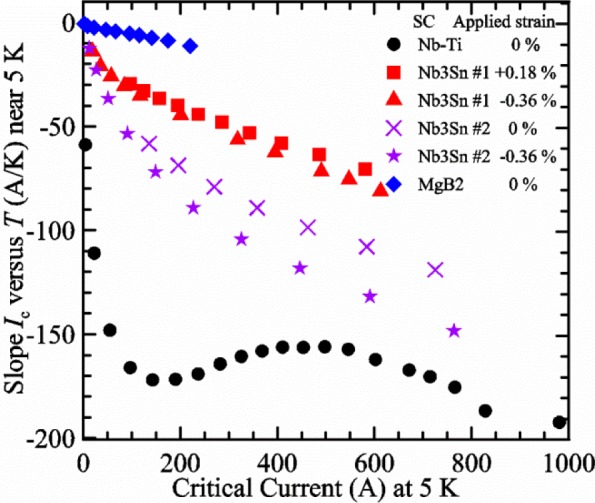
Slope of critical current versus temperature near 5 K plotted versus critical current at 5 K for various samples and applied strains.

**Fig. 6:**
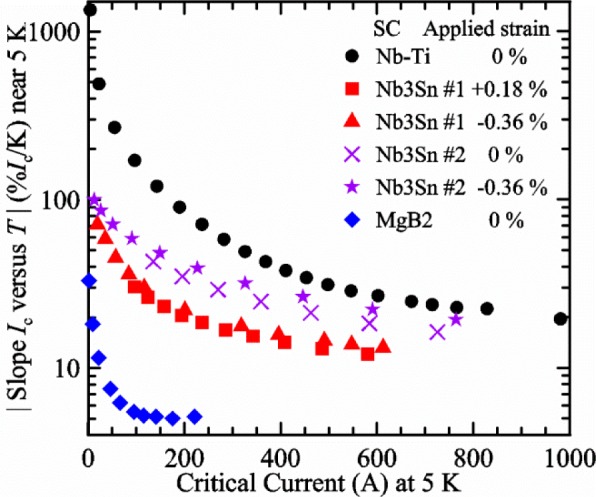
Absolute value of the slope of critical current versus temperature near 5 K plotted versus critical current at 5 K on a semi-logarithmic scale for various samples and applied strains.

**Fig. 7:**
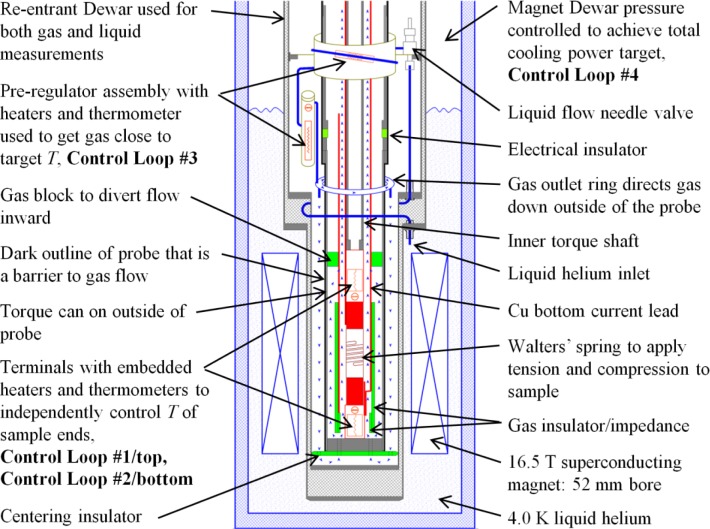
A schematic diagram of the lower part of our variable temperature measurement system shown with variable-temperature, variable-strain probe. The horizontal gaps between many components are exaggerated for clarity.

**Fig. 8:**
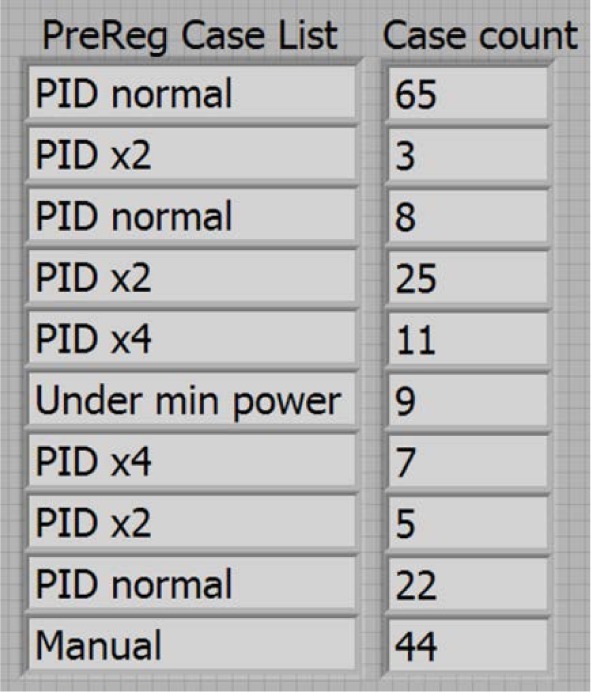
Hypothetical example pre-regulator case list that shows the ten (rows) most recent computer program logic cases (column 1) with an indicator of how many consecutive times each case occurred (column 2).

**Fig. 9:**
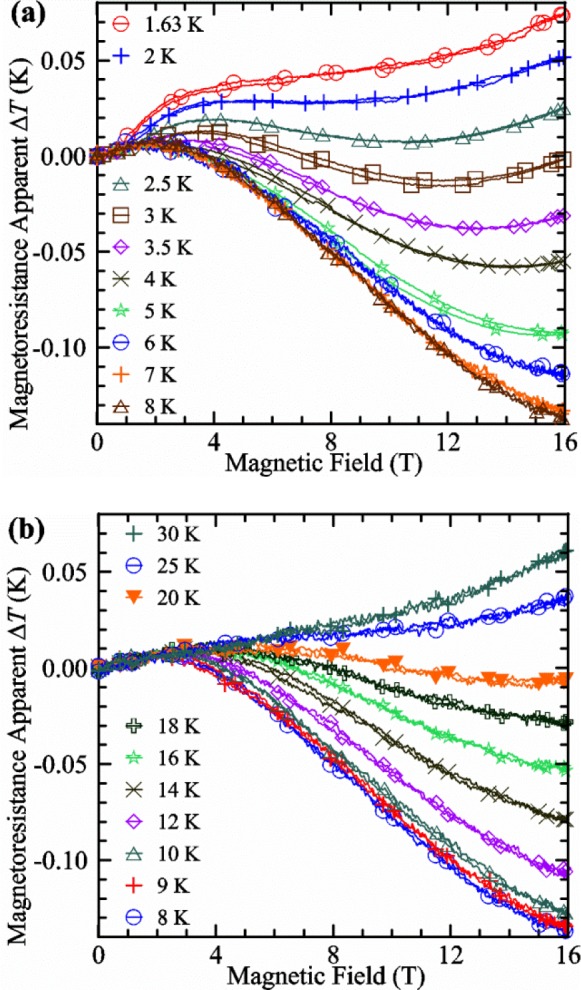
Apparent change in temperature due to magnetoresistance versus magnetic field for various temperatures from (a) 1.63 to 8 K and (b) 8 to 30 K. All of the readings are plotted, but the symbols are plotted only on every 100^th^ point.

**Fig. 10:**
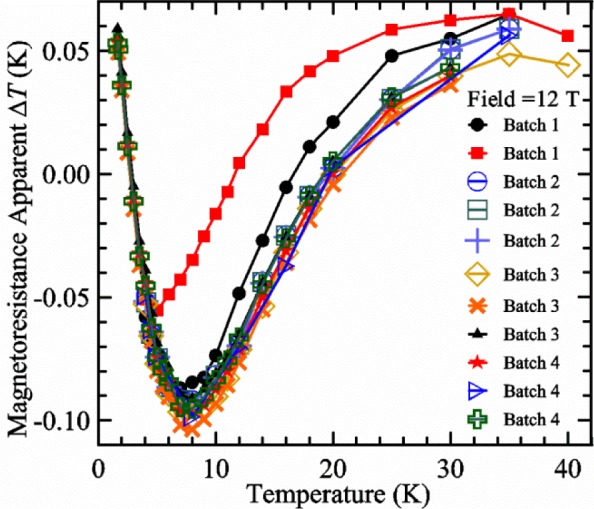
Apparent change in temperature due to magnetoresistance versus temperature at a magnetic field of 12 T for 11 resistive thermometers from four batches.

**Fig. 11:**
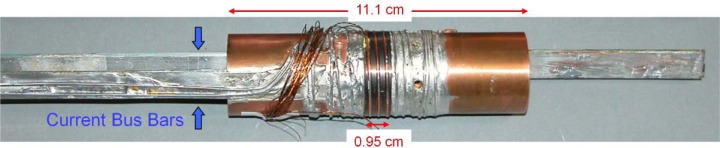
Picture of sample test fixture for variable temperature only (VTO) probe. The active 3 turns of the coil sample are wound on the oxidized Ti-6Al-4V mandrel (center 0.95 cm long) with Cu lugs on each end and current bus bars. In operation, the left direction is up.

**Fig. 12:**
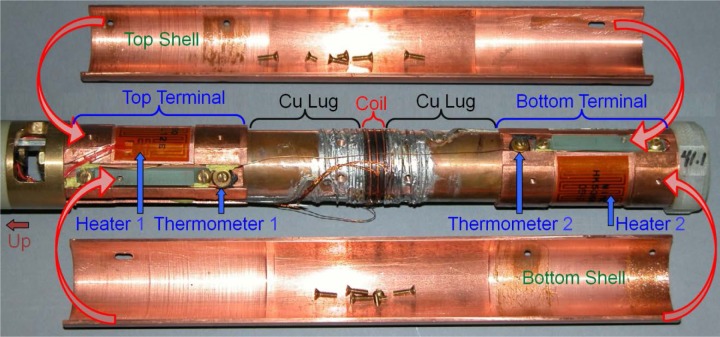
Picture of exploded view of the lower part of the variable temperature only (VTO) probe. The test fixture is bolted between the top and bottom terminals. In operation, the left direction is up. Each terminal has a thermometer and heater. The top and bottom copper shells are two halves of a cylinder. Follow the assembly arrows from the shells to the terminals to see how the two shells fit around the test fixture and terminals.

**Fig. 13:**
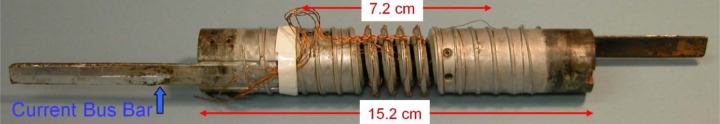
Picture of sample test fixture for variable-temperature and variable-strain (VTS) probe. The Cu-Be Walters spring is 7.2 cm tall and 2.5 cm in diameter with 4 active turns in the center. Cu lugs are screwed, pinned, and soldered to each end of the spring. Each lug has a current bus bar. Each end of the coil sample laps onto each lug and is soldered along its entire length. In operation, the left direction is up.

**Fig. 14:**
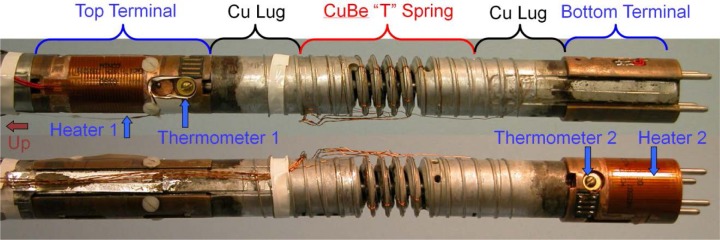
Picture of two views (probe rotated about 180° between views) of the lower part of the variable-temperature and variable-strain (VTS) probe. The test fixture is bolted and pinned between the top and bottom terminals. In operation, the left direction is up. Each terminal has a thermometer and heater. A current lead can be seen in the lower view as it attaches to the top current bus bar in a slot in the top terminal. A Cu cylinder current lead (not shown) slips over the lower part of the probe and solders to the bottom current bus bar in a slot in the bottom terminal (see upper view). The pins that stick out the bottom of the bottom terminal slip into a stainless-steel torque can that slips over the lower part of the probe.

Figure 23 shows another way to view the *liquid* and *gas* comparison at 3 T. *I*_c liq_ at 4.8 and 5 K show the expected effect of temperature, which over this small range of temperature, can be approximated as linear. There are 20 different determinations of *I*_c gas_ at various sample heater powers. We will look at the vertical distance between *I*_c gas_ and the expected line, which we call Δ*I*_c_(A) = *I_c gas_* − *I_c liq_*. We can also define Δ*I*_c_(%) = (*I_c gas_* − *I_c liq_*)/ *I_c liq_*. The higher heater powers give higher *I*_c_ values at 5 K and positive Δ*I*_c_. The lowest heater powers give *I*_c_ values that are below the expected curve and negative Δ*I*_c_. A plot of Δ*I*_c_(A) versus *H2* is shown in Fig. 24 and a plot of Δ*I*_c_(%) versus *H2* is shown in Fig. 25 for various magnetic fields where *I*_c_ varies from 5 A at 9.5 T to 750 A at 1.5 T. For any figure in this paper that has *H2* on the *x*-axis, the values of *H2* are the average values when *I* is near *I*_c_, not *H2* before the curve. Here the magnetic field is just a variable that changes the *I*_c_. At 9.5 T, the *I*_c_ is only 5 A and Δ*I*_c_(%) increases quickly with increasing *H2*, in fact, only a few measurements at the higher fields can be plotted on this scale. The percentage changes illustrate that the VTM for *I*_c_ up to 140 A are much more sensitive to heater power than at the higher currents. In general, for a given superconducting wire, the percentage change of *I*_c_ with temperature is higher at the lower critical currents, as can be seen in Figs. 1 and 2. The only possible exception to this rule is for *I*_c_ below 1 A for these wires, which would be in the tail region of the *I*_c_(*T*) curves. An important result of this work is that the protocol of these measurements is more influential when *I*_c_ is low, below 150 A, than when *I*_c_ is high for the wires measured here. All of the measurements versus *H2* are shown in the plot of Δ*I*_c_(A) in Fig. 24. The Δ*I*_c_(A) is approximately linear with *H2* and the slopes (A/W) increase with increasing *I*_c_ for the lower critical currents, then the slopes are more constant at the higher currents. The Δ*I*_c_(%) for each *I*_c_ is also approximately linear with *H2* and the slopes (%*I*_c_/W) decrease with increasing *I*_c_. When *I*_c_ is 750 A, all of the measurements with *H2* between 0.04 and 0.2 W are within 1 % of the correct value.

We can also look at the horizontal distance in Fig. 23 (see the horizontal line segments in the plot) between *I*_c gas_ and the expected line, which we call the apparent temperature error, Δ*T*. We have postulated that the dependence of Δ*I*_c_ on *H2* power is due to sub-cooling of the sample. If this is true, we should not examine Δ*I*_c_ versus *H2* power, but Δ*T* as a function of *H2* power. The relationship should represent the mechanism that changes *I*_c_ and thus, Δ*T* should be the intrinsic factor that does not depend on the sensitivity of *I*_c_ to temperature at the particular magnetic field, temperature, or strain. A plot of Δ*T* versus *H2* is shown in Fig. 26 for the same measurements as shown in Figs. 24 and 25. The slope of Δ*T* with *H2* is nearly the same (about −0.45 K/W) for *I*_c_ from 23 to 750 A for the VTO probe. Only the lowest *I*_c_ of 5 A has a different slope, which may be due to nonlinear temperature effects as *I*_c_ gets closer to zero. Whatever the cause, the 5 A *I*_c_ should be ignored. The fairly constant slope of Δ*T* with *H2* confirms the sub-cooling postulate and that the value of *H2* is a clear indicator of the sub-cooling of the sample. The four curves with *I*_c_ from 23 to 140 A fall nearly on top of each other and show that the measurement needs to be made with *H2* about 0.01 W in order to get the correct *I*_c_. As *I*_c_ increases, the curves shift to the right and the correct *H2* increases to about 0.1 W at 750 A. For all *I*_c_ values, there is a range of *H2* values where Δ*T* is within ±20 mK. For *I*_c_ values less than 150 A, *H2* needs to be less than 0.05 W for Δ*T* to be within ±20 mK.

We can plot the same Δ*T* versus *H1* as shown in Fig. 27. There are similarities between the plots of Δ*T* versus *H1* and of Δ*T* versus *H2*. The lowest *I*_c_ should be ignored again. The four curves with *I*_c_ from 23 to 140 A fall nearly on top of each other. As *I*_c_ increases, the curves shift to the right. The slope of Δ*T* with *H1* is fairly constant (−0.50 to −0.65 K/W) for *I*_c_ from 23 to 750 A for the VTO probe. In all cases, *H1* is less than *H2*.

#### Liquid/gas comparisons on other samples

These *liquid*/*gas* comparisons were also conducted on the VTO probe with a Nb-Ti sample that was soldered to a stainless-steel mandrel, with a Nb_3_Sn sample that was soldered to a stainless-steel mandrel, and with a Nb_3_Sn sample that was not soldered to a Ti-6-4 mandrel. Comparing these four cases gives information about the effect of sample type, soldering to the mandrel or not, and reproducibility.

A *liquid*/*gas* comparison on the VTO probe with Nb_3_Sn #1 sample that was soldered to a stainless-steel mandrel is shown in the next three figures. A plot of Δ*I*_c_(A) versus *H2* is shown in Fig. 28 and a plot of Δ*I*_c_(%) versus *H2* is shown in Fig. 29 for various magnetic fields where *I*_c_ varies from 79 A at 16 T to 770 A at 5 T. A plot of Δ*T* versus *H2* is shown in Fig. 30 for the same measurements as shown in Figs. 28 and 29. The Δ*T* versus *H2* plots for Nb_3_Sn #1 and the Nb-Ti (Fig. 26) samples are nearly the same. This indicates that this relationship among Δ*T*, *H2*, and *I*_c_ is an important property of the VTM system is independent of sample type. The Δ*I*_c_ plots for these same two samples have scales that are different by about a factor of 3 because the temperature dependence of the Nb_3_Sn sample is much less than that of the Nb-Ti sample at the same *I*_c_. Notice that at the highest field (16 T), the Nb_3_Sn sample still had an *I*_c_ of 79 A. The Δ*I*_c_(%) plot for the Nb_3_Sn sample illustrates that VTM are much more sensitive to heater power at the lower currents than at the higher currents, which is also what was observed on the Nb-Ti sample.

A key value from each curve in Fig. 26 and 30 is the *H2* value where Δ*T* is zero. The value depends on *I*_c_ and, if this comparison is reproducible, shows what *H2* value will give the correct *I*_c_ when measuring in *gas*. The target *H2*(*I*_c_) is an important part of the correct protocol. A plot of *H2* where the Δ*T* is zero versus *I*_c_ for the four cases are shown in Fig. 31. The two Nb_3_Sn #1 samples had the same heat treatment and were measured at magnetic fields from 5 T (soldered) and 5.5 T (not-soldered) to 16 T. The values of *I*_c_ at 16 T and 5 K (soldered 79 A and not-soldered 107 A) were somewhat different for the two Nb_3_Sn #1 samples because they had different strain states due to the different mandrel materials and possibly variations from sample to sample. The results on the two Nb-Ti samples are very close to each other. The curve for the Nb_3_Sn sample that was soldered is also close to that of the Nb-Ti samples. The curve for the Nb_3_Sn sample that was not soldered is shifted (by ~0.03 W) to slightly higher *H2* values. Using the slope of −0.45 K/W, this power shift is equivalent to a *T* shift of about 0.014 K. We have seen slight changes in *I*_c_, of segments of Nb_3_Sn samples that are not soldered to the measurement mandrel, with thermal cycling, which was attributed to changes in the strain state of various segments of the sample and the strain sensitivity of *I*_c_ in Nb_3_Sn. Thus, we think that the slight shift to higher *H2* values is due to changes in the strain state of the sample between the *liquid* and *gas* measurements, which included violent heating (10 to 15 W) to boil away the remaining liquid helium and *T* cycling to regain *T* control back at 5 K. These results demonstrate that reproducibility and appropriate *H2* values are unrelated to sample type and whether or not the sample was soldered to a mandrel.

A plot of the value of *H1* where the Δ*T* is zero versus *I*_c_ for the four cases is shown in Fig. 32. The measurements on the two Nb-Ti samples are very close to each other. The curves for the two Nb_3_Sn #1 samples are also close to those of the Nb-Ti samples. These values of *H1* are lower than the values of *H2* in all cases.

We think that Fig. 31 illustrates a key result of our variable temperature measurements from all of the systems we have studied; at low *I*_c_ (less than 150 A) the correct *gas* measurements were taken with *H2* between 0 W and 0.01 W. This verified the hypothesis of the high-gas flow system with a pre-regulator since one would expect that the thermometers and the sample would be at the same temperature under these conditions. We think that low *I*_c_ here is defined by the amount of self- and contact-heating. With very little self- and contact-heating, the sample needs only a little cooling power to stay at temperature. As *I*_c_ increases, the *H2* value where Δ*T* is zero increases nonlinearly with *I*_c_. These *H2* values indicate the effective cooling power of gas as it flows over *H2*. The effective cooling power of gas as it later flows over the sample should scale with *H2*; it may be dependent on relative surface area and constriction of flow. When the current was near 750 A, the self-heating of the sample was estimated to be about 0.01 W, and the heating of the two current contacts was estimated to be about 0.02 W (top contact) and 0.07 W (bottom contact). At 750 A, the *H2* value where Δ*T* is zero is about 0.1 W, which is about the sum of the self- and contact-heating. Some of the contact heat will flow to the terminals and reduce the heat necessary to hold the terminal temperature, and the remaining power will need to be carried away by the cooling power of the gas. As can be seen in Fig. 21, both *H1* and *H2* decreased by about 0.1 W after the current ramped up to 750 A. These results indicate that determining appropriate *H2* values as a function of *I*_c_ is an important part of the protocol to obtain the correct *gas* measurements. Additionally, the total heater power as a function of *I*_c_ is part of the protocol to keep *H1* and *H2* from going to zero and losing control.

We typically perform a *liquid*/*gas* comparison periodically to verify that the curve of *H2* where the Δ*T* is zero, has not changed significantly due to changes in contact resistance, self-heating, or gas flow paths. If there is a section of the sample along the active length that has a lower *I*_c_, this can lead to significant self-heating and can be a terminal flaw for the sample. We monitor the voltage drop along the entire active length of the sample to detect excess voltage drop and self-heating.

There is some relationship between *H1* and *H2* values because of their proximity, so there will also appear to be some correlation of results with *H1*; however, we tested this and will present those results later in this paper. The relationship between the two heater powers does allow us to operate the pre-regulator control loop with a target for *H1* or for *H2*. One advantage of using *H1* as the target is that it is generally closer to zero power than *H2*; thus the feedback will operate to keep *T1* in control. The pre-regulator control loop heater target becomes the value prior to the *V-I* curve, since more time is spent with the current “off” than “on.” Thus the target also needs to account for the decrease in sample heater powers that occurs when the sample current comes on as illustrated in Fig. 19. For the VTO probe, the value of *H2* when *I* is near *I*_c_ can generally be obtained so that the apparent temperature error Δ*T* is within ±0.02 K of zero. If more precision is required, two *I*_c_ measurements can be made with different heater powers and the correct *I*_c_ can be estimated by interpolation or slight extrapolation to the correct *H2*. This estimation is equivalent to using two points from Fig. 24 to determine what *I*_c_ would be at a certain *H2*. The estimation can be done without knowing the temperature dependence of *I*_c_ that would be needed to use a plot similar to Fig. 26.

#### Measurements at temperatures above 5 K

We cannot do a *liquid*/*gas* comparison at temperatures above 5 K, so this leaves the question somewhat open as to whether the same protocol leads to different results at higher temperatures where gas flow rates, thermal conductivity, and heat capacity are different. However, we can still measure *I*_c gas_ above 5 K versus heater power and see how it varies. We cannot know the correct *I*_c_, but we can measure the approximate *T* dependence at two different temperatures and nearly the same heater power and use an arbitrary *I*_c gas_ at the lowest heater power as the correct value. Then, we can make plots that look very similar to Figs. 26 and 27, except the curves are shifted arbitrarily so that Δ*T* = 0 at the lowest heater power. The slope of Δ*T* versus heater power at any *T* is not arbitrary and gives some information about the effect of heater power at higher temperatures. A plot of the slope of Δ*T*(*H2*) in units of K/W versus *I*_c_ is shown in Fig. 33 for the four cases (two samples, two solder conditions) and various temperatures from 5 K to 12 K. All of the data at 5 K (all black symbols) are based on *liquid*/*gas* comparisons. As before, we ignore the slopes at very low currents. Solid symbols indicate that the sample was soldered to the mandrel. Red symbols indicate that *T* was above 5 K. All of these slopes are within a narrow band of values (range about 0.2 K/W) and are relatively independent of *I*_c_. The average slope is −0.41 K/W at 5 K and −0.39 K/W for *T* greater than 5 K, suggesting that a protocol based on heater power may remove the other effects of temperature. Thus, we expect the protocol to remain similar for a reasonable range of temperatures.

For our type of VTM system, we still expect that the correct low *I*_c_ values will be measured with *H2* values that are less than 0.02 W regardless of the temperature. As we have seen in *liquid* and *gas* comparisons at 5 K, as *I*_c_ increases for a given conductor, the percentage change of *I*_c_ with temperature decreases and thus, if slope of Δ*T*(*H2*) stays constant or decreases with temperature, then the protocol at high critical currents is not as crucial at any temperature.

A plot of the slope of Δ*T*(*H1*) in units of K/W versus *I*_c_ is shown in Fig. 34 for the four cases (two samples, two solder conditions) and various temperatures from 5 K to 12 K. The figure utilizes the same legend as shown in Fig. 33. All of the data at 5 K (all black symbols) are based on *liquid*/*gas* comparisons. As before, we ignore the slopes at very low currents. Solid symbols indicate that the sample was soldered to the mandrel. Red symbols indicate that *T* was above 5 K and tend to be higher than the black symbols. All of the slopes are within a range of about 0.3 K/W and show a tendency to become a more negative value with increasing *I*_c_. The average slope is −0.50 K/W at 5 K and −0.36 K/W for *T* greater than 5 K.

#### Relative influence of H1 and H2 and of T1 and T2; Nb-Ti sample soldered to stainless-steel mandrel in VTO probe

We designed an experiment to study the relative influence of *H1* and *H2* and of *T1* and *T2* to attempt to verify our hypothesis that *T2* and *H2* are more influential than *T1* and *H1*. We introduced a temperature bias, *T* bias, defined as *T1*−*T2*, where *T* bias = 0, 10, 20, and 30 mK. We made measurements in *gas* with *T2* = 5 K, then at the highest *T* bias had *T1* = 5.03 K. Increasing *T* bias has two effects: it attempts to create a temperature gradient over the length of the sample and it changes the relationship between *H1* and *H2* by increasing *H1*. The helium gas flow will attempt to remove the temperature gradient, and the effective temperature of the sample will indicate the extent to which the gradient is reduced.

For this study we used a Nb-Ti wire soldered to a stainless-steel mandrel on the VTO probe using a non-superconducting In-Ag solder. Reference plots of Δ*I*_c_(A), Δ*I*_c_(%), and Δ*T*(K) versus *H2* with *T* bias set to 0 mK are given in Figs. 35–37. The study included measurements to a record highest *I*_c_ *_gas_*, 981 A at 0.8 T, for the VTO probe. These high-current results are consistent with previous results and show that most of the measurements, at tested *H2* values, are within 1 % of the correct value. However, the results with *I*_c_ *_gas_* > 650 A were slightly less reproducible.

We measured the apparent temperature error Δ*T* versus *H2* at 7.5 T and *T2* = 5 K where *I*_c_ *_liq_* was 143 A with *T* bias values of 0, 10, 20, and 30 mK as shown in Fig. 38. Since *T1* and *T2* are different, we must determine an appropriately weighted temperature, *Tw*. For Fig. 38, we used *Tw* = 0.5 *T1* +0.5 *T2*, which is the simple average of the two temperatures. The extreme case would have *Tw* = *T2*, which, with a *T* bias of 30 mK, would only shift *Tw* by 15 mK. Thus this study is focused on a small effect. With equal weighting of the two thermometers, the *H2* value where Δ*T* is zero is 13, 6, −1, and −9 mW for *T* biases of 0, 10, 20, and 30 mK, respectively. Negative values of *H2* where Δ*T* is zero are not realistic, based on the previous findings. Therefore, we need to determine different temperature weights that gives more consistent results. We found, by iteration to minimize the difference in the *H2* value where Δ*T* is zero, that *Tw* = 0.14 *T1* +0.86 *T2* collapses the Fig. 38 data onto essentially one line, as shown in Fig. 39. With this *Tw* equation, the *H2* value where Δ*T* is zero is 9.6, 10.2, 10.3, and 9.4 mW for *T* biases of 0, 10, 20, and 30 mK. A plot of Δ*T* versus *H1* for the same data set and *Tw* equation is shown in Fig. 40. As expected, values of *H1* increase for a given *H2* with increasing *T* bias. This demonstrates that the values of *T1* and *H1* are less influential than values of *T2* and *H2*.

The value of *H2* where Δ*T* is zero for *T* bias = 0 mK changed slightly from 13 mW to 10 mW with *Tw* equation because *T1* and *T2* were not exactly equal when *I*_c_ was measured in *liquid*. The difference between *T1* and *T2* in *liquid* was 4 mK at 7.5 T and varied from 0 to 5 mK for the 22 magnetic fields due to: calibration error, magnetoresistance correction error, noise, and hydrostatic head. We decided to use the same *Tw* equation to calculate the temperature assigned to each *I*_c_ *_liq_* value to effectively create a consistent “thermometer” for the comparison. This was a very minor effect and it is mentioned only for completeness.

Measurements of Δ*T* versus *H2* were also made at a number of magnetic fields/critical currents and *T* biases as summarized in Fig. 41. The figure shows that *H2* where Δ*T* is zero has a similar dependence on *T* bias over the whole range of *I*_c_ values with the simple average *Tw* equation. When *Tw* = 0.14 *T1*+0.86 *T2* was used to determine the correct *I*_c_, as shown in Fig. 42, effectively all of the Fig. 41 data collapsed onto one curve, indicating that the scaling optimized on 143 A also works from 4 to 981 A. Figure 43 shows *H1* where Δ*T* is zero versus *I*_c_ with the same *Tw* equation (*Tw* = 0.14 *T1*+0.86 *T2*), illustrating that the value of *H1* where the correct *I*_c_ is determined can be systematically varied by changing *T* bias. In general, we did not need to operate the VTO probe using a non-zero *T* bias, as can be seen in Fig. 43, because the values of *H1* where Δ*T* = 0 are all positive or very close to zero for all critical currents.

The *T*-bias experiment focused on a small effect. For a *T* bias of 30 mK, changing from *Tw* = 0.5 *T1* +0.5 *T2* to *Tw* = 0.14 *T1* +0.86 *T2* only reduces *Tw* from 5.015 K to 5.0042 K. So, at the largest *T* bias studied here, the shift in *T* is only 0.0108 K. Generally, a non-zero *T* bias is not necessary to operate the VTO probe; however, using a finite *T* bias with the VTS probe was found to be useful and allowed *H2* to be lower without *H1* going to zero.

The *T*-bias experiment could not separate the influence of *T1* and *H1*. However, a *Tw* equation that seems reasonable, considering the gas flow direction, scales the results. Thus *T1* has only a slight influence on the correct protocol, and the value of *H1* may have no influence. This is a consideration because the relationship between *H1* and *H2* sometimes changes with time and with temperature, and one wants to know which heater power to consider in the correct protocol. Slight changes in the relationship are likely due to changes in gas-flow patterns caused by the shifting of the probe in the dewar, blockages that develop or change with time, and relative changes in thermometer calibrations. The findings from the *T*-bias experiment on the VTS probe given below support the postulate that the value of *H1* is not an influential parameter for these measurements.

### 3.3 Verification Measurements with the Variable-Temperature and Variable-Strain (VTS) Probe

#### Liquid/gas comparisons on a Nb-Ti sample mounted and soldered on Cu-Be spring

**Fig. 15:**
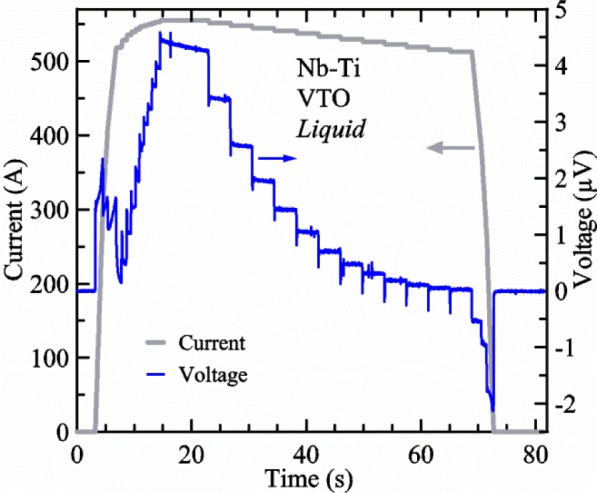
Sample current (left axis) and voltage (right axis) versus time measured on a Nb-Ti sample at 3 T and 5 K in liquid helium, and using the VTO probe. There are about 4,100 readings of each the two signals.

**Fig. 16:**
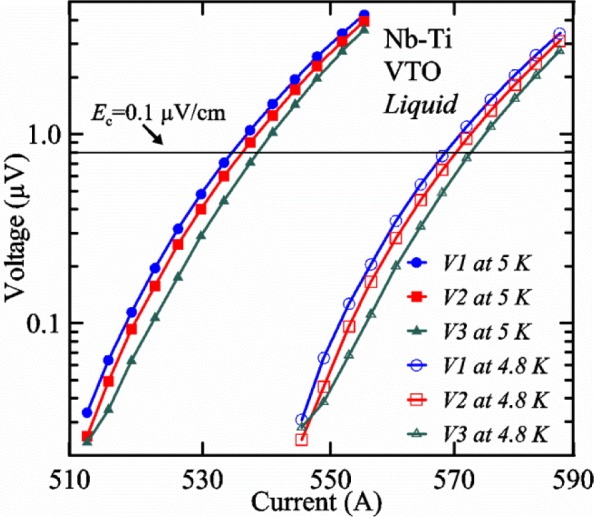
A full-logarithmic scale plot of *V-I* curves for three voltage taps at two temperatures in liquid helium measured on a Nb-Ti sample at 3 T using the VTO probe.

**Fig. 17:**
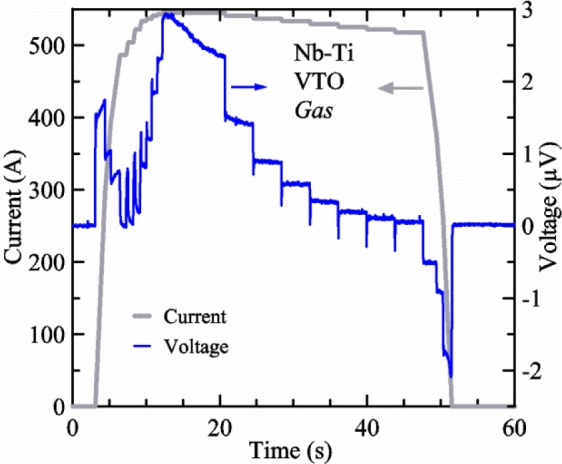
Sample current (left axis) and voltage (right axis) versus time measured on a Nb-Ti sample at 3 T and 5 K, in flowing helium gas, using the VTO probe. There are about 3,000 readings of each the two signals.

**Fig. 18:**
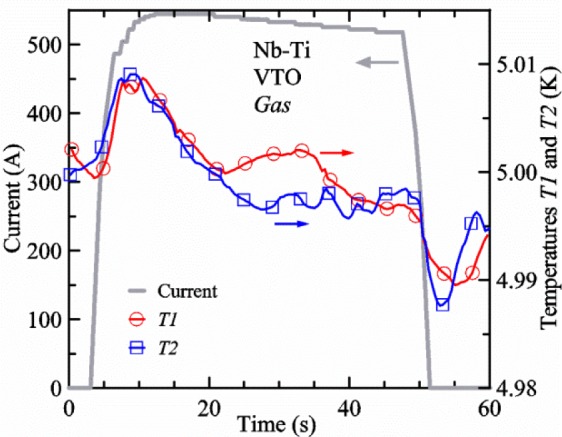
Sample current (left axis) and temperatures *T1* and *T2* (right axis) versus time measured on a Nb-Ti sample at 3 T and 5 K, in flowing helium gas, and using the VTO probe. All of the readings are plotted, but the symbols for the two temperatures are plotted only on every 10^th^ point. These data were taken during the same curve as in Fig. 17.

**Fig. 19:**
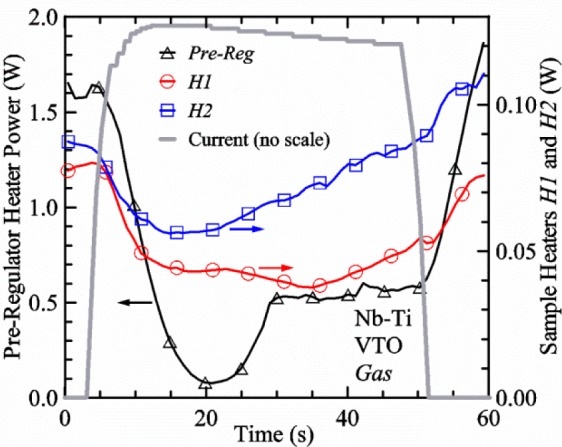
Pre-regulator heater power (left axis), sample heater powers *H1* and *H2* (right axis), and sample current (no scale) versus time measured on Nb-Ti sample at 3 T and 5 K, in flowing helium gas, and using the VTO probe. The symbols for the powers are plotted only on every 5^th^ point. These data were taken during the same curve as in Figs. 17 and 18.

The next 13 figures contain data that were taken on a single tap (*V1*) and Nb-Ti wire, using the VTS probe with the sample mounted and soldered to the Cu-Be spring. The influence of *T* bias is included from the beginning and it was studied with twice the range of *T* biases because the region of interest for *H2* had *H1* values near zero or ideally (not physically possible) slightly negative. The temporal plots using the VTS probe were very similar to those of the VTO probe shown in Figs. 15–19, so we will not show those figures for the VTS probe.

**Fig. 20:**
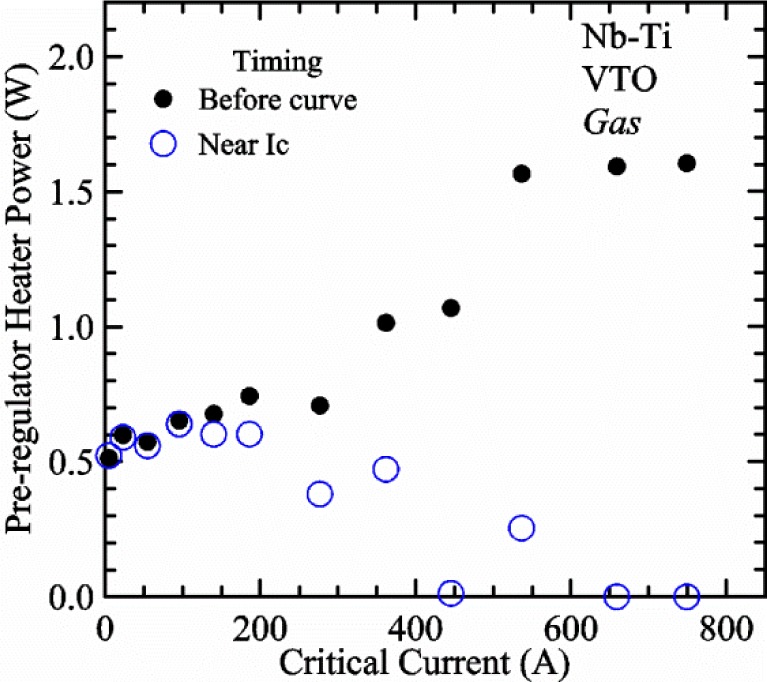
Pre-regulator heater power versus critical current for a set of measurements on a Nb-Ti sample at 5 K, in *gas*, and on the VTO probe. The solid circle symbols indicate the average heater power before each *V-I* curve was taken and the open circle symbols indicate the average heater power during the time that the sample current was near *I*_c_.

**Fig. 21:**
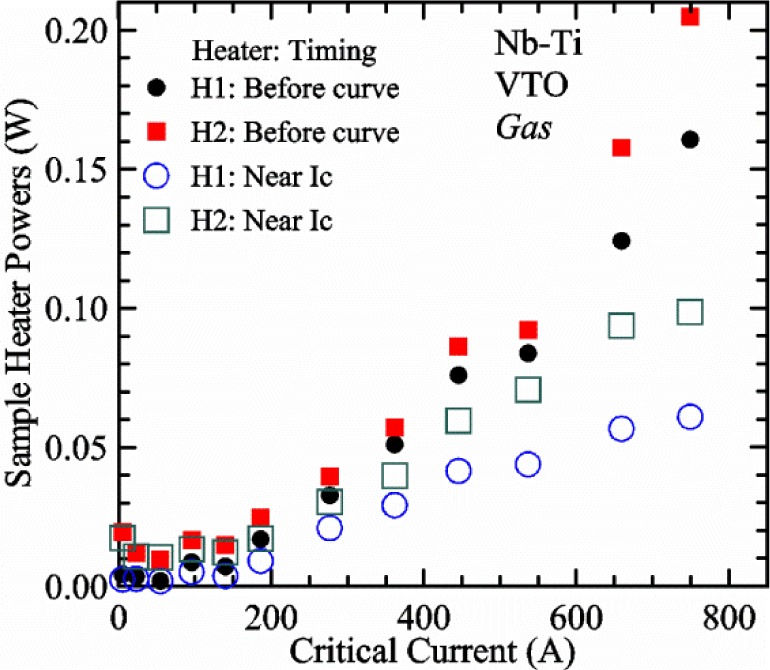
Sample heater powers *H1* and *H2* versus critical current for a set of measurements on a Nb-Ti sample at 5 K in gas, and on the VTO probe. The solid symbols indicate the average heater power before each *V-I* curve was taken and the open symbols indicate the average heater power during the time that the sample current was near *I*_c_.

**Fig. 22:**
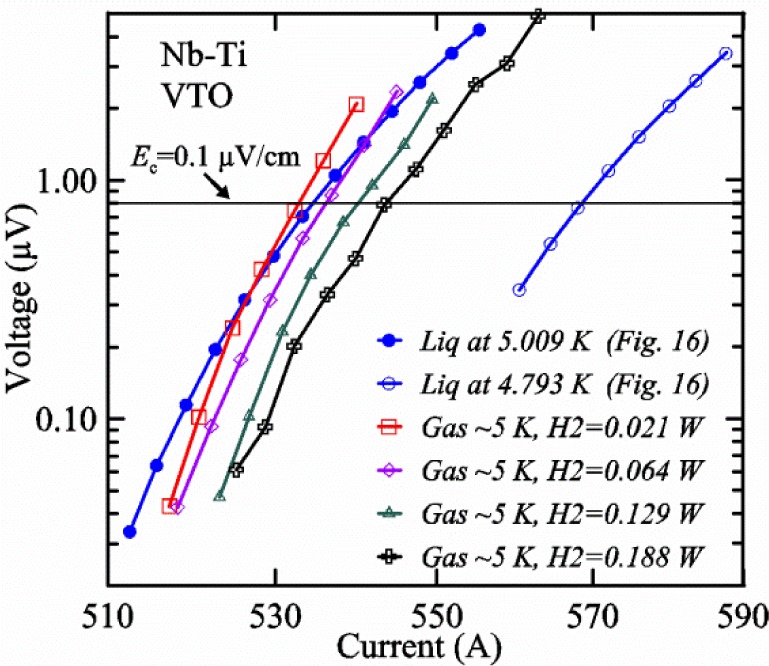
A full-logarithmic scale plot of *V-I* curves measured on a Nb-Ti sample in *liquid* and in *gas* with different *H2* powers (average when *I* near *I*_c_) using the VTO probe. Part of the curve at 4.8 K in *liquid* is covered by the legend.

**Fig. 23:**
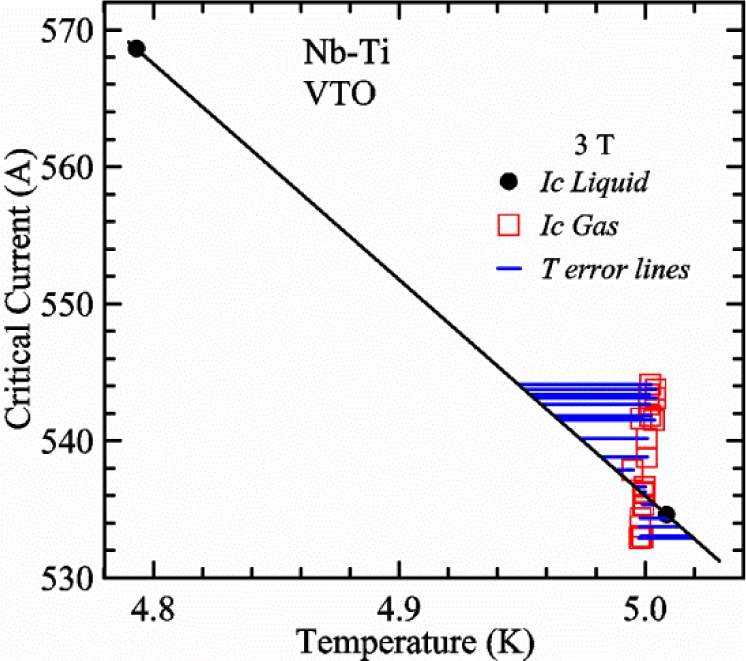
Critical current versus temperature on a Nb-Ti sample measured using the VTO probe in *liquid* helium and in flowing helium *gas* at 3 T. The solid black line indicates the expected linear dependence of *I*_c_(*T*). The horizontal blue lines indicate the *T* error for the measurements in *gas* with different heater power levels.

**Fig. 24:**
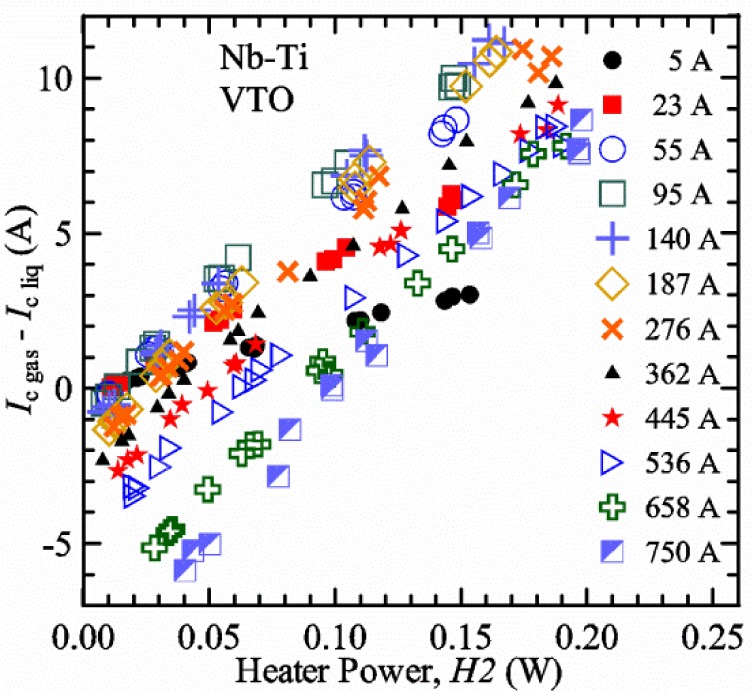
Difference *I_c gas_* − *I_c liq_* versus the average heater power of control loop #2 (*H2*) at various magnetic fields where *I*_c_ varies from 5 A to 750 A at 5 K for a Nb-Ti wire measured on the VTO probe. The measured *I_c gas_* varies systematically with *H2* heater power.

**Fig. 25:**
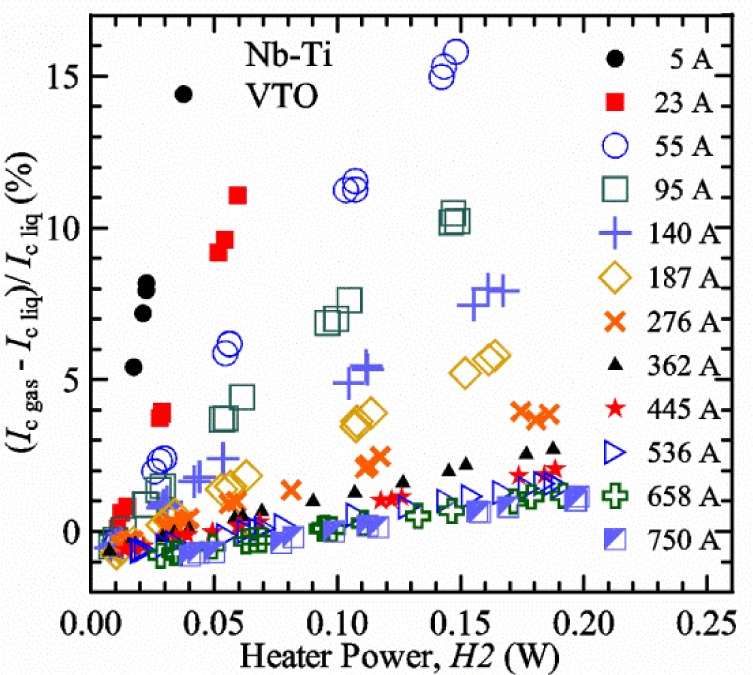
Percentage difference (*I_c gas_* − *I_c liq_*)/*I_c liq_* versus the average heater power of control loop #2 (*H2*) at various magnetic fields where *I*_c_ varies from 5 A to 750 A at 5 K for a Nb-Ti wire measured on the VTO probe. The measured *I_c gas_* varies systematically with *H2* heater power.

**Fig. 26:**
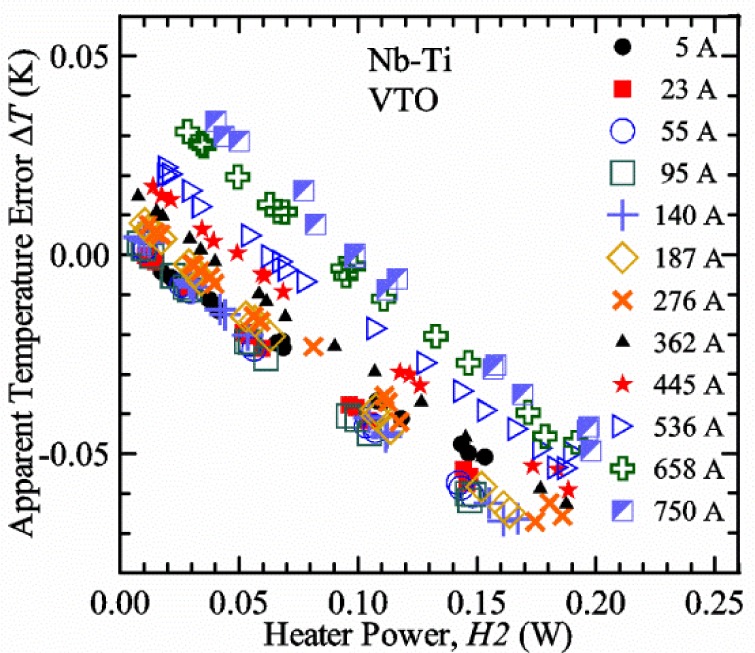
Apparent temperature error Δ*T* versus the average heater power of control loop #2 (*H2*) at various magnetic fields where *I*_c_ varies from 5 A to 750 A at 5 K for a Nb-Ti wire measured on the VTO probe. These data are from the same set shown on Figs. 24 and 25.

Figure 44 shows the average *Pre-Reg* power just prior to the *V-I* curve (solid circles) and the average power during the time when the current is near *I*_c_ (open circles) as a function of *I*_c_ for a Nb-Ti sample at 5 K in *gas*. We varied the applied magnetic field to increase *I*_c_ from about 5 A at 9.5 T to 708 A at 1.75 T. The *T* bias was set to 0 mK for these measurements. Most of the discussion points made on the corresponding Fig. 20 for the VTO probe apply to Fig. 44 for the VTS probe. Since we needed to acquire a matrix of data at different *H2* and *T* bias values at each *I*_c_, some of the *Pre-Reg* power points may not have been in static equilibrium, which likely caused the non-monotonic differences between the two powers with *I*_c_. For this sequence of measurements, the target *Pre-Reg* values were high enough that the *Pre-Reg* power did not go to zero during any of the *V-I* curves.

Figure 45 shows the average *H1* and *H2* powers just prior to the *V-I* curve (solid symbols) and the average powers during the time when the current is near *I*_c_ (open symbols) as a function of *I*_c_ for a Nb-Ti sample at 5 K in *gas*. These measurements were made for the same curves as those shown in Fig. 44. The *T bias* was set to 0 mK for these measurements. The somewhat higher *H2* at the lowest *I*_c_ is likely due to the system not being completely stable when data acquisition started. The target *H1* power is about 0.005 W for low *I*_c_ and is about 0.08 W for *I*_c_ of 708 A. These targets are set based on prior knowledge of how much the power decreases during a curve. Based on the *liquid*/*gas* comparison, *H2* needs to be a little lower than is possible without *H1* going to zero in order to obtain the correct *I*_c_ *_gas_*. Thus, the goal for the VTS probe is to have *H1* be as low as possible when the current is near *I*_c_. For this set of data when the current is near *I*_c_, *H1* was between 0.001 and 0.01 W.

To lower the obtainable *H2*, we need a *T* bias that increases the cooling power for *H1*. Figure 46 shows the average *Pre-Reg* power just prior to the *V-I* curve (solid circles) and the average power during the time when the current is near *I*_c_ (open circles) as a function of *I*_c_. The *T* bias (*T1*−*T2*) was set to 60 mK for these measurements. *T* bias did not have a noticeable change to *Pre-Reg* power. Figure 47 shows the average *H1* and *H2* powers just prior to the *V-I* curve (solid symbols) and the average powers during the time when the current is near *I*_c_ (open symbols) as a function of *I*_c_ with *T* bias = 60 mK. This *T* bias allowed *H1* to be close to or above *H2* just prior to the curve and allowed *H2* to be low without *H1* going to zero when the current was on, as can be seen by comparing *H2* values on Figs 45 and 47. *H1* dropped more when the current was on than *H2* did in both figures, which was due to a higher heat load from the sample current on the top terminal.

A plot of Δ*I*_c_(A) versus *H2* is shown in Fig. 48 and a plot of Δ*I*_c_(%) versus *H2* is shown in Fig. 49 for various magnetic fields where *I*_c_ varies from 5 A at 9.5 T to 708 A at 1.75 T with *T* bias = 0 mK. For any figure in this paper that has *H2* on the *x*-axis, the values of *H2* are the average values when *I* is near *I*_c_, not *H2* just prior to the *V-I* curve. At 9 T, the *I*_c_ is 24 A and Δ*I*_c_(%) increases quickly with increasing *H2*; in fact, only a few measurements at the higher fields can be plotted on this scale. When *I*_c_ *_liq_* = 97 A, an *H2* of about 0.1 W causes *I*_c_ *_gas_* to be more than 20 % higher than the correct value. Comparing Figs. 36 (VTO) and 49 (VTS) shows that the Δ*I*_c_(%) of the VTS probe is about 2.5 times more sensitive to heater power than the VTO probe for the same sample. The percentage changes illustrate that VTM for *I*_c_ below 200 A are much more sensitive to heater power than those at the higher currents. An important result of this work is that the measurement protocol is more influential when *I*_c_ is below 200 A. All of the measurements versus *H2* are shown in a plot of Δ*I*_c_(A) in Fig. 48. The Δ*I*_c_(A) is approximately linear with *H2* and the slopes (A/W) increase with increasing *I*_c_ for the lower critical currents, then the slopes are more constant at the higher currents. The Δ*I*_c_(%) for each *I*_c_, is also approximately linear with *H2* and the slopes (%*I*_c_/W) decrease with increasing *I*_c_.

The apparent temperature error Δ*T* versus *H2* is shown in Fig. 50 for the same measurements as shown in Figs. 48 and 49. The slope of Δ*T* with *H2* is nearly the same (about −1.28 K/W) for *I*_c_ from 5 A to 708 A for the VTS probe. The slope is about 2.8 times the value found for the VTO probe. The fairly constant slope of Δ*T* with *H2* confirms the sub-cooling postulate and that the value of *H2* is a clear indicator of the sub-cooling of the sample. The six curves with *I*_c_ from 5 A to 189 A fall nearly on top of each other and show that the measurement needs to be made with *H2* at most 0.01 W in order to get the correct *I*_c_. As *I*_c_ increases, the curves shift to the right and the correct *H2* increases to about 0.046 W at 708 A. For all *I*_c_ values, there is a range of *H2* values where Δ*T* is within 50 mK, but none of the available *H2* values gives a positive Δ*T*, in contrast to the findings for the VTO probe shown in Fig. 37.

**Fig. 27:**
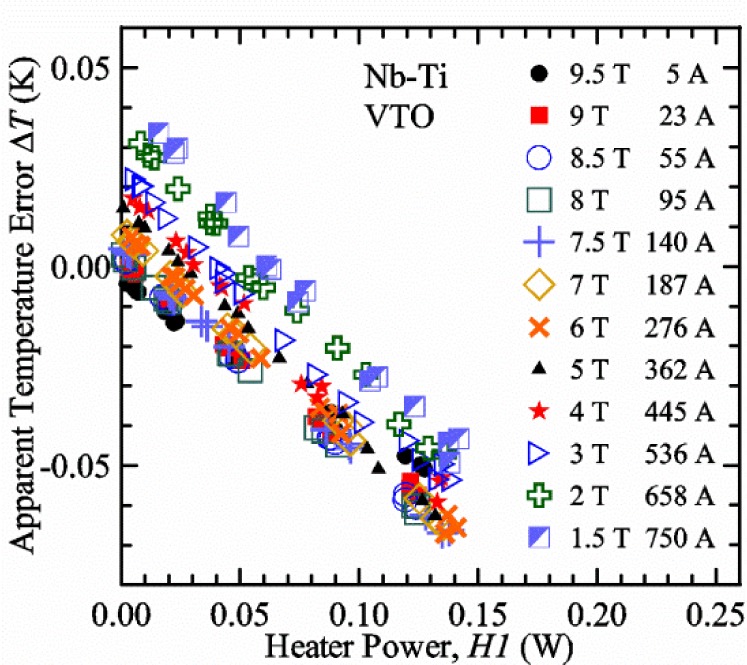
Apparent temperature error Δ*T* versus the average heater power of control loop #1 (*H1*) at various magnetic fields where *I*_c_ varies from 5 A to 750 A at 5 K for a Nb-Ti wire measured on the VTO probe. These data are from the same set shown on Figs. 24–26.

**Fig. 28:**
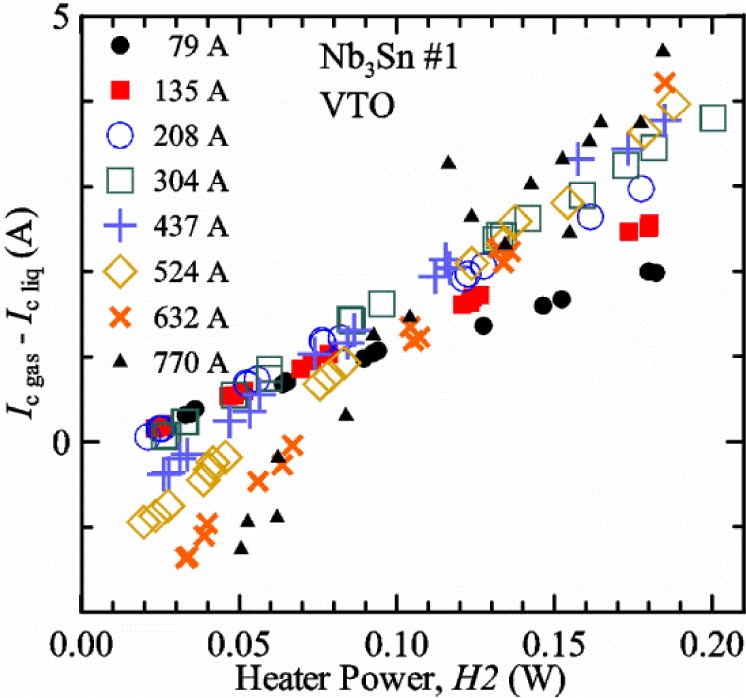
Difference *I_c gas_* − *I_c liq_* versus the average heater power of control loop #2 (*H2*) at various magnetic fields where *I*_c_ varies from 79 A to 770 A at 5 K for Nb_3_Sn #1 soldered to a stainless-steel mandrel and measured on the VTO probe.

**Fig. 29:**
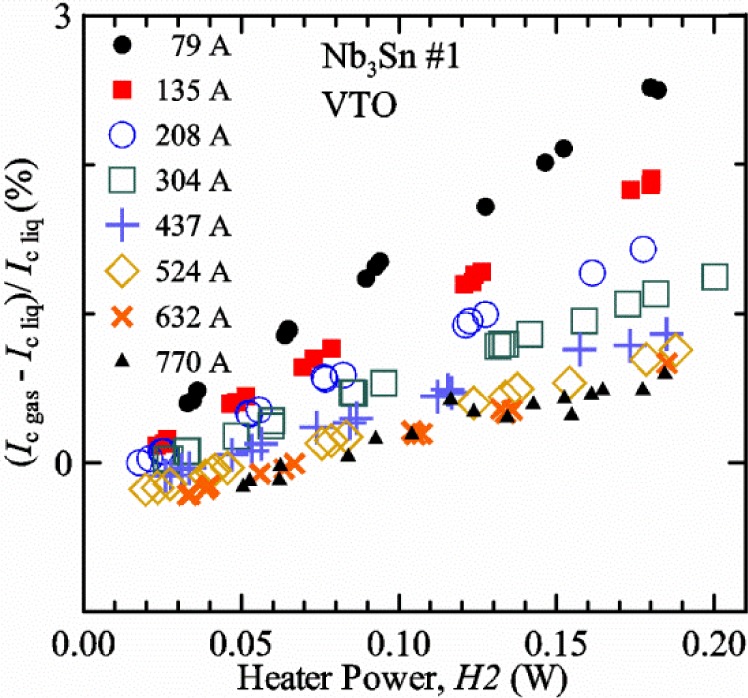
Percentage difference (*I_c gas_* − *I_c liq_*)/*I_c liq_* versus the average heater power of control loop #2 (*H2*) at various magnetic fields where *I*_c_ varies from 79 to 770 A at 5 K for Nb_3_Sn #1 probe soldered to a stainless-steel mandrel and measured on the VTO.

**Fig. 30:**
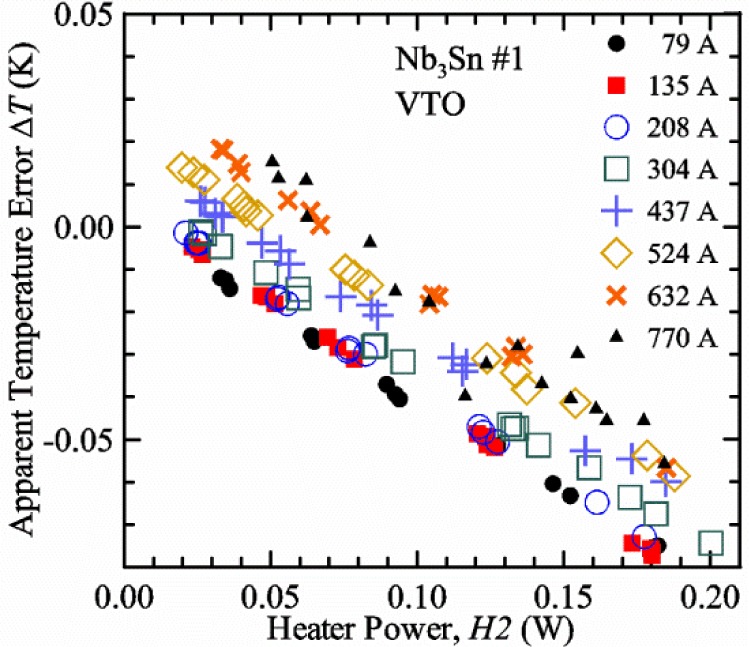
Apparent temperature error Δ*T* versus the average heater power of control loop #2 (*H2*) at various magnetic fields where *I*_c_ varies from 79 to 770 A at 5 K for Nb_3_Sn #1 soldered to a stainless-steel mandrel and measured on the VTO probe. These data are from the same set shown on Figs. 28 and 29.

A plot of Δ*T* versus *H1* is shown in Fig. 51 for the VTS probe, and it is very different from the corresponding Fig. 27 for the VTO probe. All of the curves in Fig. 51 fall nearly on top of each other. The slope of Δ*T* with *H1* is fairly constant (−1.90 to −2.25 K/W) for *I*_c_ from 5 A to 708 A for the VTS probe. All *H1* values where Δ*T* is zero are negative and range from −0.005 W to −0.015 W with *T* bias = 0 mK. This result shows that no extra *H1* heating power is needed as the self- and contact-heating increase with *I*_c_. It also indicates that the attempt to have some of the gas flow bypass *H2* and the sample to directly cool *H1* was largely unsuccessful. However, there is one distinct advantage of this result: all of *H1* values where Δ*T* is zero are about the same, −0.01 W, regardless of *I*_c_. This negative power is not achievable, thus a finite *T* bias is needed.

We measured Δ*T* versus *H2* at 7.5 T and *T2* = 5 K in *gas* where *I*_c_ *_liq_* was 143 A with *T* bias values of 0, 20, 40, and 60 mK and using *Tw* = 0.5 *T1* +0.5 *T2* as shown in Fig. 52. With the equal weighting of the two thermometers, the *H2* value where the Δ*T* is zero, are 5, 0, −5, and −9 mW for *T* biases of 0, 20, 40, and 60 mK, respectively. Negative values of *H2* where Δ*T* is zero are not realistic based on the previous findings. Therefore, we need to determine a new temperature weight that gives more consistent results. We found, by iteration to minimize the discrepancies in the *H2* values where Δ*T* is zero, that *Tw* = 0.18 *T1* +0.82 *T2* collapses the Fig. 52 data onto essentially one line in Fig. 53. With this *Tw* equation, the *H2* value where the Δ*T* is zero is 2.0, 2.3, 1.6, and 2.1 mW for *T* biases of 0, 20, 40, and 60 mK, respectively. As with the VTO probe results, the value of *H2* where Δ*T* is zero for *T* bias = 0 mK changed with the *Tw* equation because *T1* and *T2* were not exactly equal when measured in *liquid* and the same *Tw* equation was used to calculate the temperature assigned to each *I*_c_ *_liq_* value.

Measurements of Δ*T* versus *H2* were also made at a number of magnetic fields/critical currents and *T* biases as summarized in Fig. 54. The figure shows that *H2* where Δ*T* is zero has a similar dependence on *T* bias over the whole range of *I*_c_ values with the simple average *Tw* equation. When *Tw* = 0.14 *T1*+0.86 *T2* was used to determine the correct *I*_c_, as shown in Fig. 55, essentially all of the Fig. 54 data collapsed onto one curve, indicating that the scaling optimized on 143 A also works from 5 A to 708 A. Figure 56 shows *H1* where Δ*T* is zero versus *I*_c_ with *Tw* = 0.14 *T1*+0.86 *T2*. The figure illustrates that the value of *H1* where the correct *I*_c_ is determined can be systematically varied by changing *T* bias. A *T* bias of 20 mK shifts the average value of *H1* for the correct *I*_c_ to about 0 W. A *T* bias of 40 mK or higher allows *H1* to be positive when Δ*T* = 0 for the full range of *I*_c_.

#### Liquid/gas comparisons on other samples

The *liquid*/*gas* comparisons were also conducted on the VTS probe with the two Nb_3_Sn samples and one MgB_2_ sample that were soldered to the T-section spring. We use the Nb-Ti results as a reference. A comparison of results from the four samples gives information about the effect of sample type and reproducibility. A key value from each curve in Fig. 50 is the *H2* value where Δ*T* is zero. This value depends on *I*_c_ and, if the comparison is reproducible, the value indicates the *H2* power that will yield the correct *I*_c_ when measuring in *gas*. A plot of *H2* where Δ*T* is zero versus *I*_c_ for the seven cases are shown in Fig. 57. Each of the two Nb_3_Sn samples was measured with different applied strains, and in some cases, on the very same specimen. One Nb_3_Sn #1 sample was measured at a strain of 0.18 % where *I*_c_ was 97 A at 16 T and at a strain of −0.36 % where *I*_c_ was 19 A at 16 T. An applied strain of −0.36 % was selected so that a low value of *I*_c_ could be achieved at our highest magnetic field of 16 T. The results from the two Nb_3_Sn #1 specimens are close to each other and are only slightly higher than those of the Nb-Ti sample. Nb_3_Sn #2 was measured at three strains where the first two strains in the legend (−0.42 % and −0.39 %) correspond to the same specimen but different bypass flows (flow that we attempt to direct past *H2* to help independently cool *H1*). We cannot quantify the amount of bypass flow, but Nb_3_Sn #2 (−0.42 %) had the least bypass flow and Nb_3_Sn #2 (−0.39 %) had the most bypass flow. The relative amount of bypass flow was determined by the extremes of relative impedance that we could achieve (see Sec. 2.1). The curves for these two cases of Nb_3_Sn #2 are close to each other and only slightly lower than those of the Nb-Ti sample, except for one point at the highest current. A more significant difference was observed, as expected, between these two flow cases in the slope of Δ*T*(*H1*) and Δ*T*(*H2*) as shown below. We typically operate in the configuration of the most bypass flow, but as we have described, the bypass flow is not very effective. The Nb_3_Sn #2 specimen measured at −0.36 % strain was one of the first measurements that we made with this probe, and we think that the self- and contact-heating was larger than for the other measurements, which likely caused the results to be the most different from those of the Nb-Ti sample. The results from the MgB_2_ sample were limited to currents less than 220 A, but they were in good agreement with the Nb-Ti results even though the temperature sensitivity of the MgB_2_ sample was significantly less than that of the Nb-Ti wire. For *I*_c_ less than 200 A, all seven cases have *H2* = 0 to within ±10 mW. Using an approximate Δ*T*/*H2* slope of −1.2 K/W, a 10 mW change in *H2* is equivalent to a *T* shift of about 0.012 K. At about 600 A, the maximum difference from the Nb-Ti sample curve (not counting Nb_3_Sn #2 −0.36 %) is about 20 mW, which is equivalent to a *T* shift of about 0.024 K. For the worst case, the equivalent *T* shift is about 0.041 K. Again, remember that for a given conductor, the percentage change in *I*_c_ for a given change in *T* is less at the higher currents. These results demonstrate reproducibility and that appropriate *H2* values are relatively independent of sample type and bypass flow.

A plot the *H1* where Δ*T* is zero versus *I*_c_ for the seven cases are shown in Fig. 58. These measurements were made with a *T* bias of zero. Thus, as expected, almost all of the *H1* values where Δ*T* is zero are less than zero. For *I*_c_ less than 300 A, all of the cases are between zero and −20 mW. The curves for the two Nb_3_Sn #1 samples are close to each other. Except for the highest current, the curves for the two Nb_3_Sn #2 samples are also close to each other despite the difference in bypass flow.

**Fig. 31:**
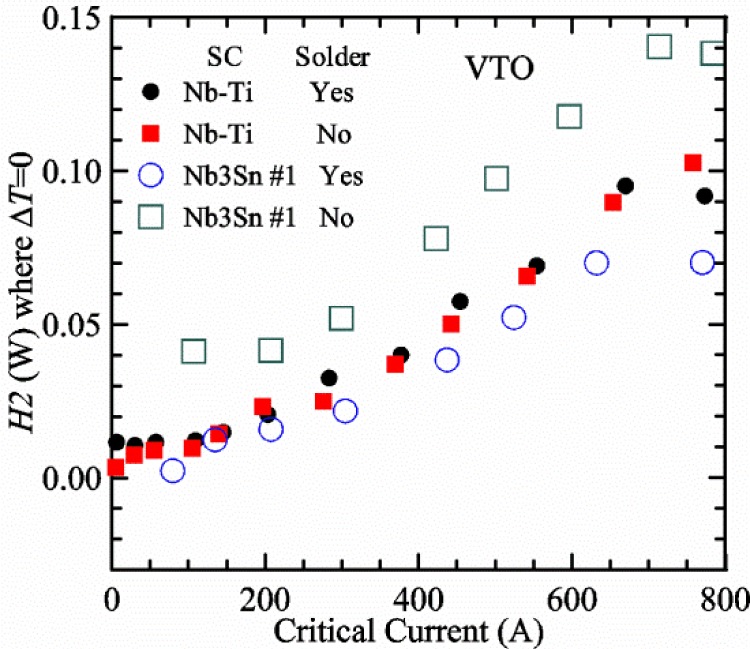
Plot of *H2* where Δ*T* = 0 versus *I*_c_ for Nb-Ti and Nb_3_Sn #1 samples that were soldered and not soldered to the sample mandrel of the VTO probe. Determining the appropriate *H2* values as a function of *I*_c_ is an important part of the protocol to obtain the correct *gas* measurements.

**Fig. 32:**
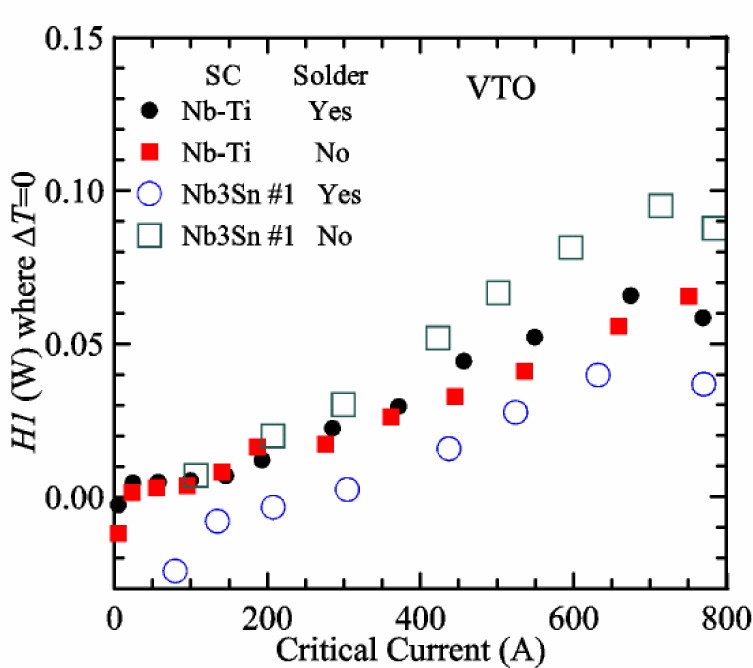
Plot of *H1* where Δ*T* = 0 versus *I*_c_ at 5 K for Nb-Ti and Nb_3_Sn #1 samples that were soldered and not soldered to the sample mandrel of the VTO probe.

We think that Figs. 31 and 57 illustrate a key result of our variable temperature measurements from all of the systems we have studied: at low *I*_c_ (less than 150 A) the correct *gas* measurements were taken with *H2* between −0.01 W and 0.01 W. This verified the hypothesis of the high-gas flow system with a pre-regulator since one would expect that the thermometers and the sample would be at the same temperature under these conditions.

**Fig. 33:**
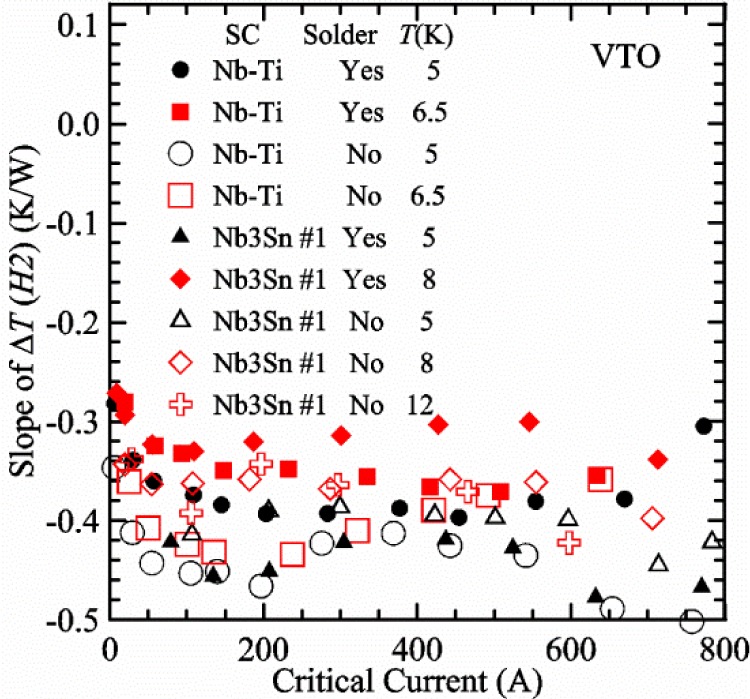
Plot of slope of the Δ*T*(*H2*) versus *I*_c_ for Nb-Ti (*T* = 5 and 6.5 K) and Nb_3_Sn #1 (*T* = 5, 8, and 12 K) samples that were soldered and not soldered to the sample mandrel of the VTO probe.

**Fig. 34:**
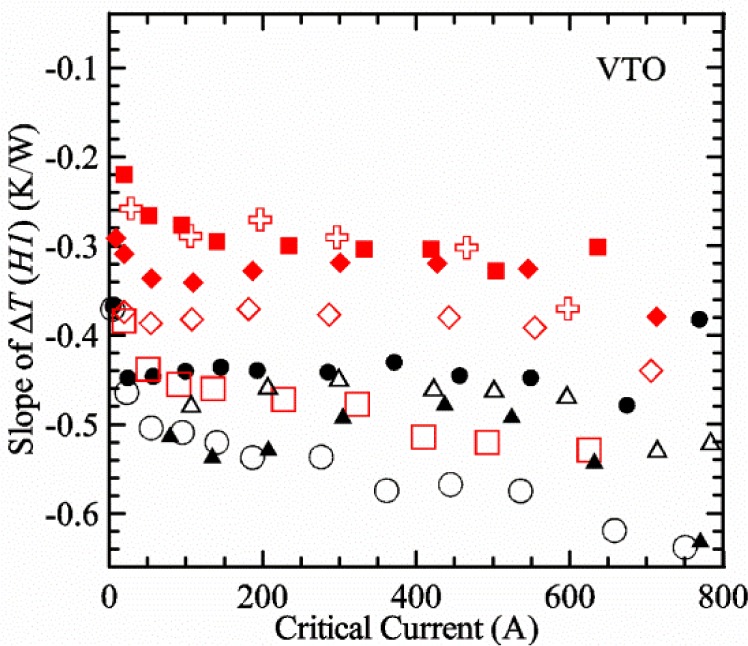
Plot of slope of the Δ*T*(*H1*) versus *I*_c_ for Nb-Ti (*T* = 5 and 6.5 K) and Nb_3_Sn #1 (*T* = 5, 8, and 12 K) samples that were soldered and not soldered to the sample mandrel of the VTO probe. Same legend as Fig. 33.

**Fig. 35:**
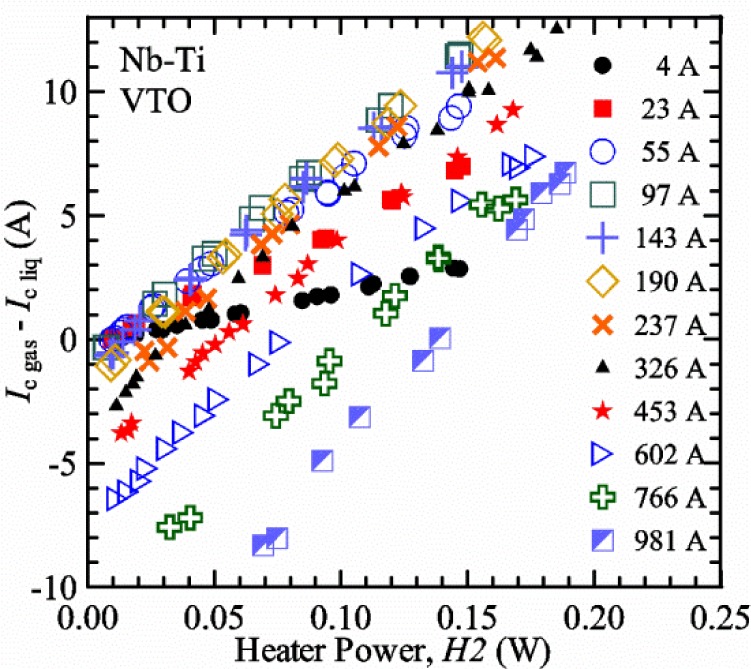
Difference *I_c gas_* − *I_c liq_* versus *H2* at various magnetic fields where *I*_c_ varies from 4 to 981 A at 5 K for a Nb-Ti wire soldered to a stainless-steel mandrel on the VTO probe.

**Fig. 36:**
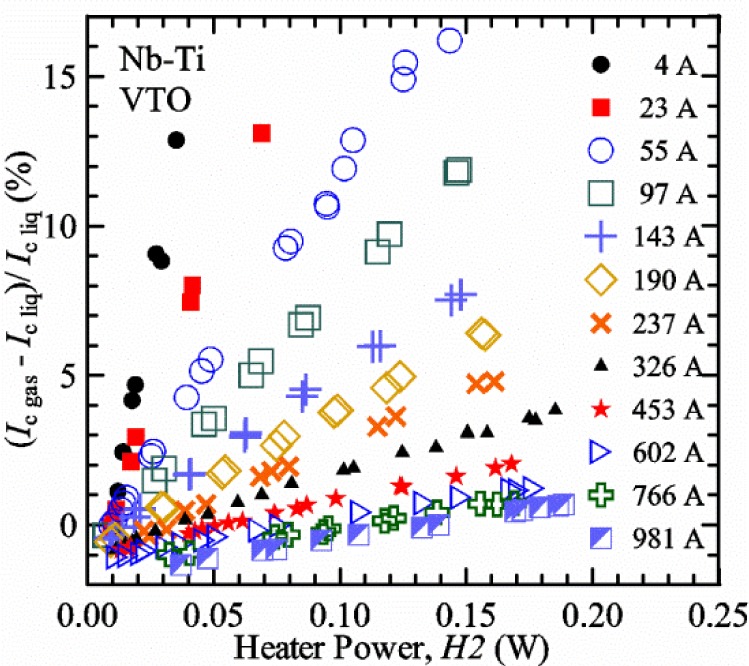
Percentage difference (*I_c gas_* − *I_c liq_*)/ *I_c liq_* versus *H2* at various magnetic fields where *I*_c_ varies from 4 to 981 A at 5 K for a Nb-Ti wire soldered to a stainless-steel mandrel on the VTO probe.

**Fig. 37:**
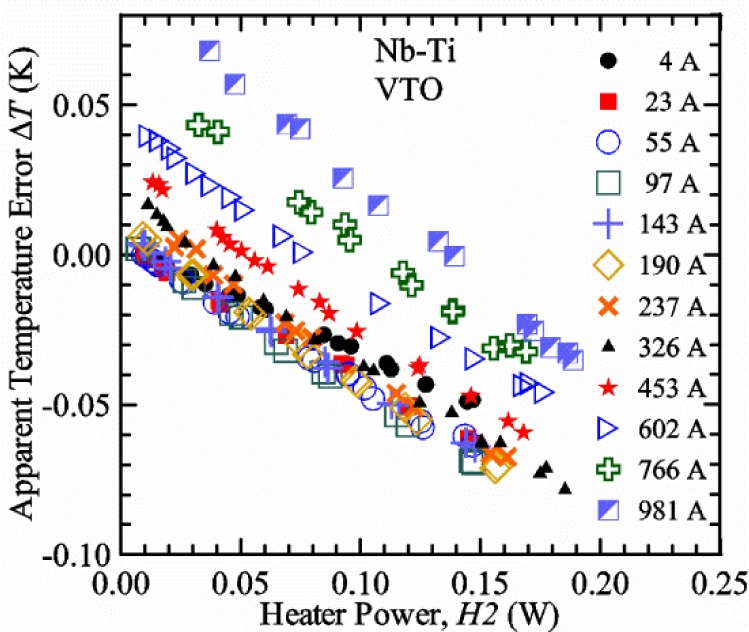
Apparent temperature error Δ*T* versus *H2* at various magnetic fields where *I*_c_ varies from 4 to 981 A at 5 K for a Nb-Ti wire soldered to a stainless-steel mandrel on the VTO probe. These data are from the same set shown on Figs. 35 and 36.

**Fig. 38:**
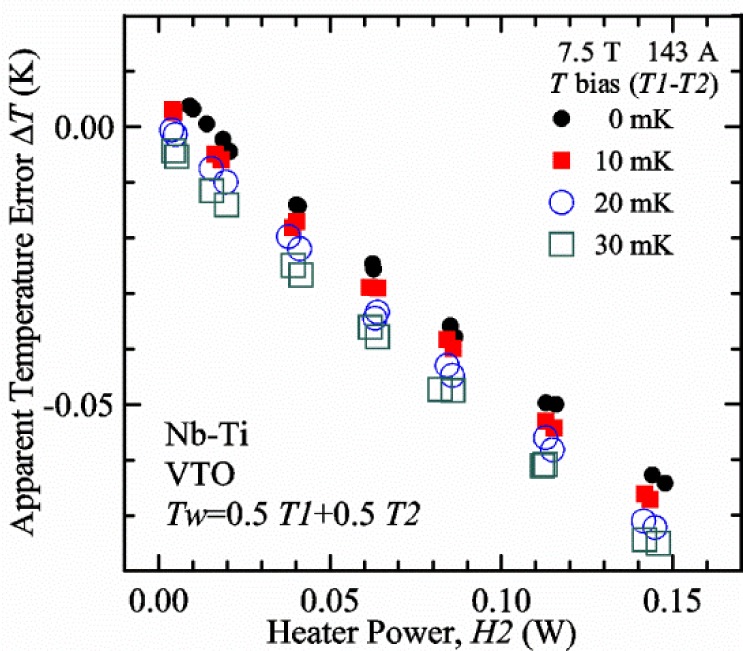
Apparent temperature error Δ*T* versus *H2* at 7.5 T and *T2* = 5 K where *I*_c_ is about 143 A for a Nb-Ti wire soldered to a stainless-steel mandrel on the VTO probe. The *T* bias (*T1−T2*) was set to 0, 10, 20, and 30 mK. The weighted temperature *Tw* = 0.5 *T1*+0.5 *T2* was used to determine the correct *I*_c_.

**Fig. 39:**
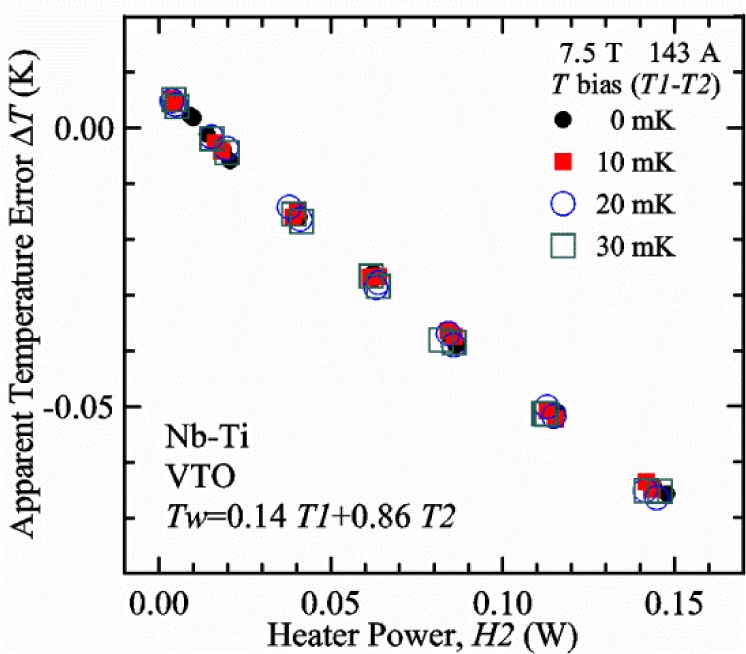
Apparent temperature error Δ*T* versus *H2* at 7.5 T and *T2* = 5 K where *I*_c_ is about 143 A for a Nb-Ti wire soldered to a stainless-steel mandrel on the VTO probe. The *T* bias (*T1−T2*) was set to 0, 10, 20, and 30 mK. The weighted temperature *Tw* = 0.14 *T1*+0.86 *T2* was used to determine the correct *I*_c_, which collapsed the Fig. 38 data onto one line.

**Fig. 40:**
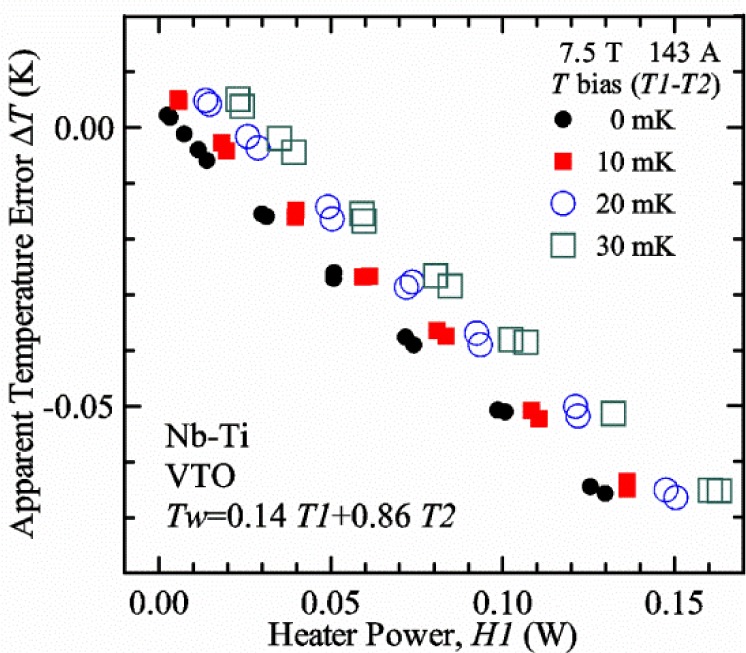
Apparent temperature error Δ*T* versus *H1* at 7.5 T and *T2* = 5 K where *I*_c_ is about 143 A for a Nb-Ti wire soldered to a stainless-steel mandrel on the VTO probe. The *T* bias (*T1*−*T2*) was set to 0, 10, 20, and 30 mK. The weighted temperature *Tw* = 0.14 *T1*+0.86 *T2* was used to determine the correct *I*_c_. This shows that the values of *T1* and *H1* are less influential than *T2* and *H2*.

**Fig. 41:**
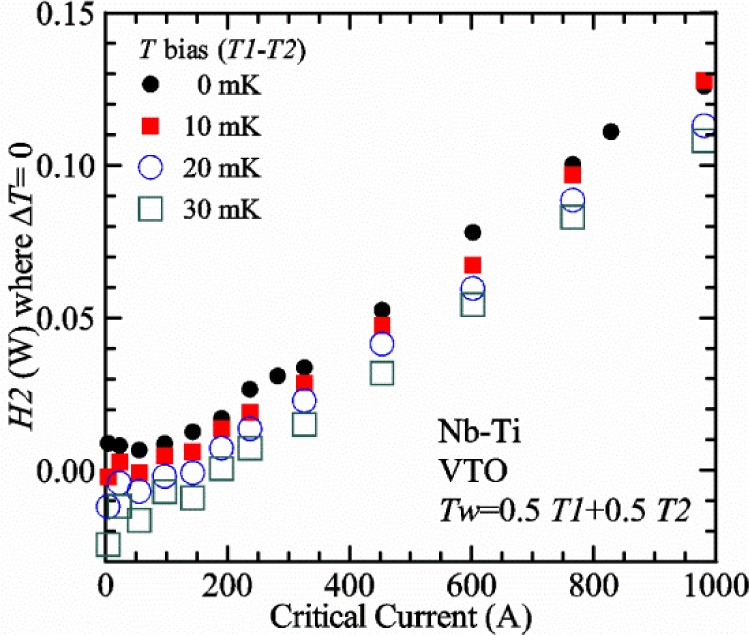
Plot of *H2* where Δ*T* = 0 versus *I*_c_ at 5 K for a Nb-Ti wire soldered to a stainless-steel mandrel on the VTO probe. The *T* bias (*T1−T2*) was set to 0, 10, 20, and 30 mK. The weighted temperature *Tw* = 0.5 *T1*+0.5 *T2* was used to determine the correct *I*_c_.

**Fig. 42:**
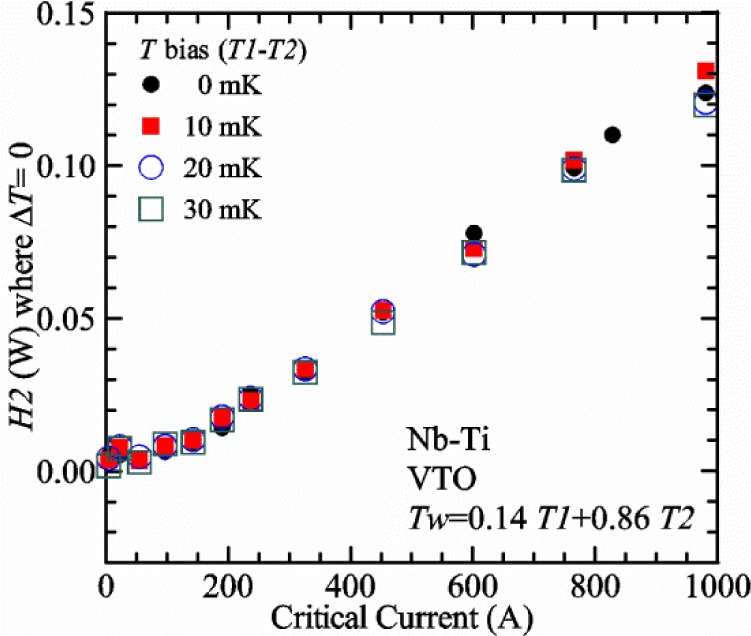
Plot of *H2* where Δ*T* = 0 versus *I*_c_ at 5 K for a Nb-Ti wire soldered to a stainless-steel mandrel on the VTO probe. The *T* bias (*T1−T2*) was set to 0, 10, 20, and 30 mK. The weighted temperature *Tw* = 0.14 *T1*+0.86 *T2* was used to determine the correct *I*_c_, which collapsed the Fig. 41 data onto one curve.

**Fig. 43:**
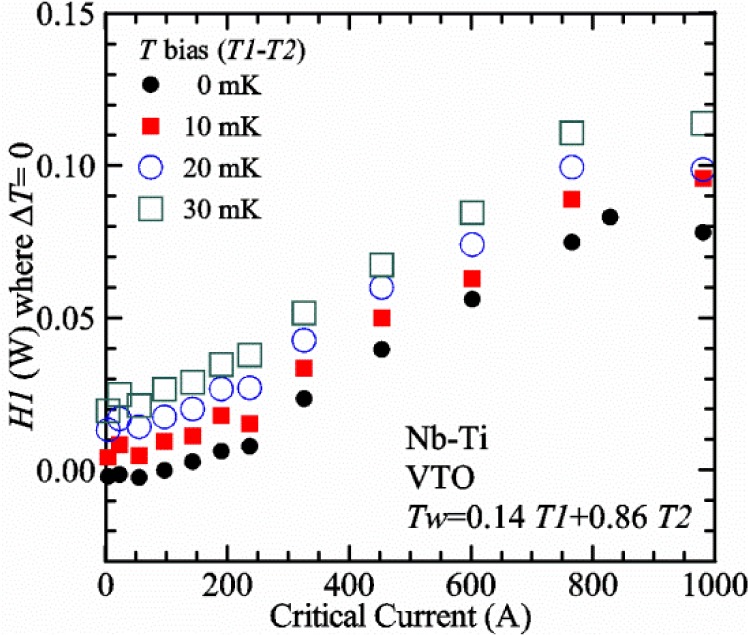
Plot of *H1* where Δ*T* = 0 versus *I*_c_ at 5 K for a Nb-Ti wire soldered to a stainless-steel mandrel on the VTO probe. The *T* bias (*T1−T2*) was set to 0, 10, 20, and 30 mK. The weighted temperature *Tw* = 0.14 *T1*+0.86 *T2* was used to determine the correct *I*_c_.

**Fig. 44:**
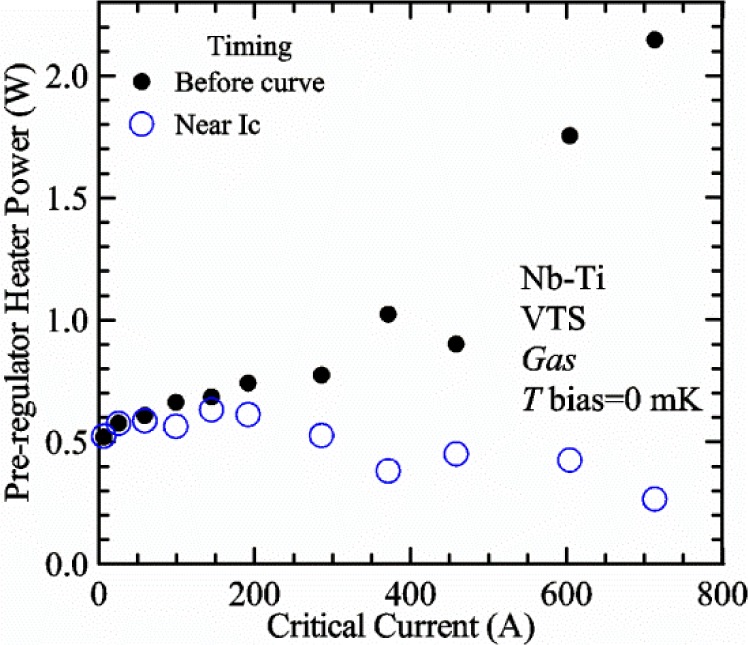
Pre-regulator heater power versus *I*_c_ for a set of measurements on a Nb-Ti sample at 5 K, in *gas*, and on the VTS probe. The solid circle symbols indicate the average heater power before each *V-I* curve was taken and the open circle symbols indicate the average heater power during the time that the sample current was near *I*_c_. *T* bias (*T1−T2*) was set to 0 mK.

**Fig. 45:**
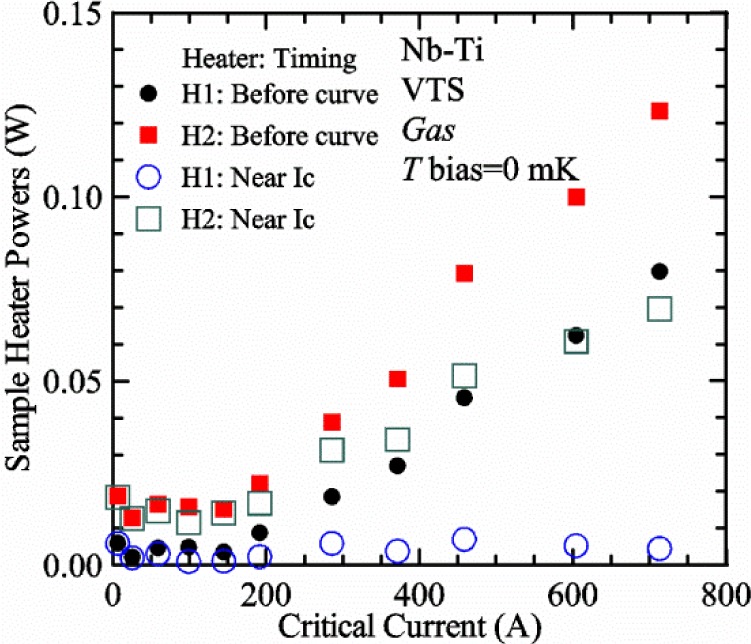
Sample heater powers *H1* and *H2* versus *I*_c_ for a set measurements on a Nb-Ti sample at 5 K, in gas, and on the VTS probe. The solid symbols indicate the average heater power before each *V-I* curve was taken and the open symbols indicate the average heater power during the time that the sample current was near *I*_c_. *T* bias (*T1−T2*) was set to 0 mK.

**Fig. 46:**
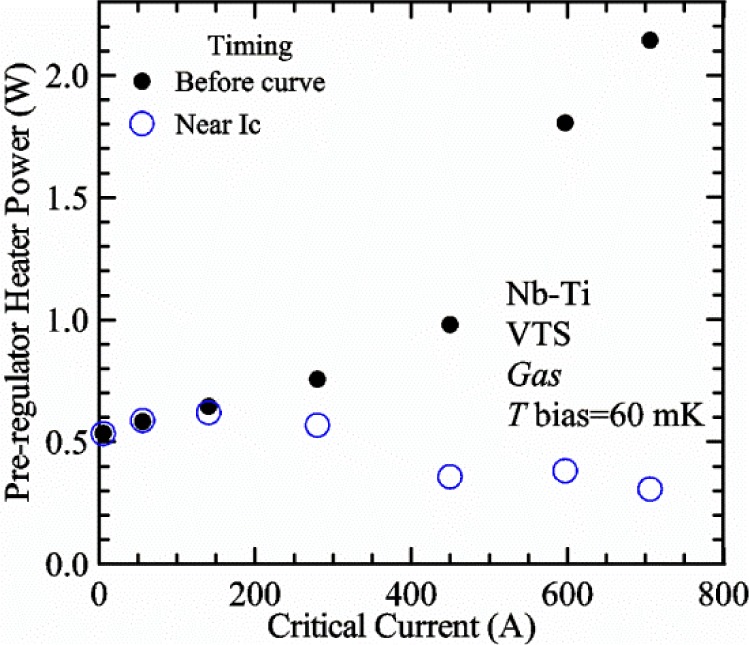
Pre-regulator heater power versus *I*_c_ for a set of measurements on a Nb-Ti sample at 5 K, in *gas*, and on the VTS probe. The solid circle symbols indicate the average heater power before each *V-I* curve was taken and the open circle symbols indicate the average heater power during the time that the sample current was near *I*_c_. *T* bias (*T1−T2*) was set to 60 mK.

**Fig. 47:**
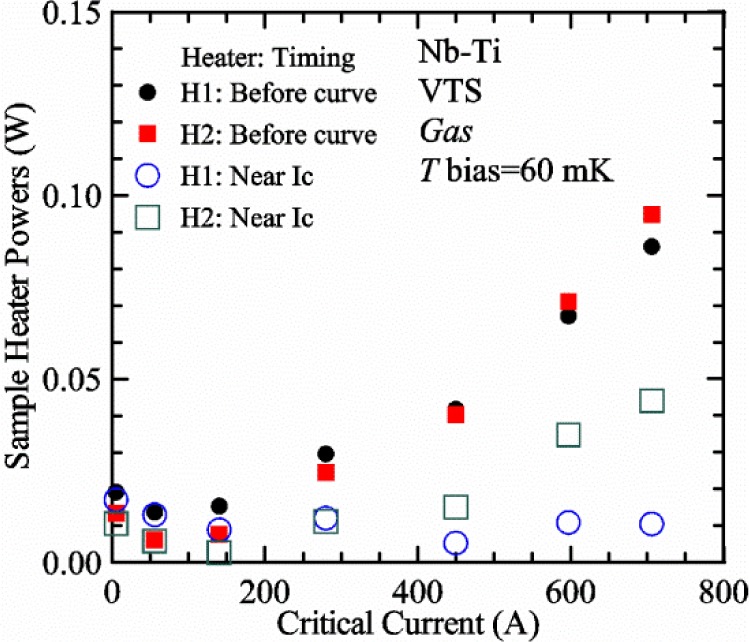
Sample heater powers *H1* and *H2* versus *I*_c_ for a set measurements on a Nb-Ti sample at 5 K, in gas, and on the VTS probe. The solid symbols indicate the average heater power before each *V-I* curve was taken and the open symbols indicate the average heater power during the time that the sample current was near *I*_c_. *T* bias (*T1−T2*) was set to 60 mK, which allowed *H2* to be lower without *H1* going to zero.

**Fig. 48:**
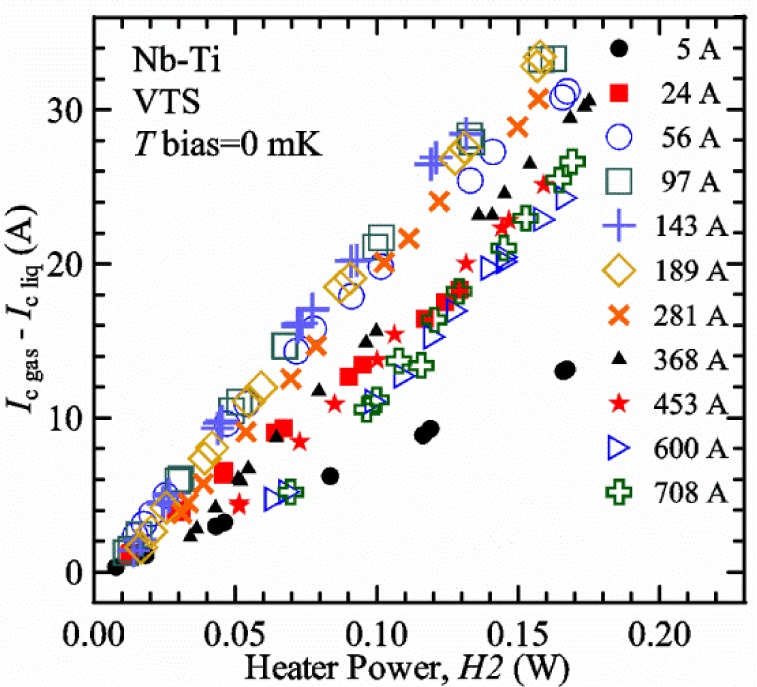
Difference *I_c gas_* − *I_c liq_* versus *H2* at various magnetic fields where *I*_c_ varies from 5 A to 708 A at 5 K for a Nb-Ti wire soldered to a Cu-Be spring on the VTS probe. *T* bias (*T1−T2*) was set to 0 mK.

**Fig. 49:**
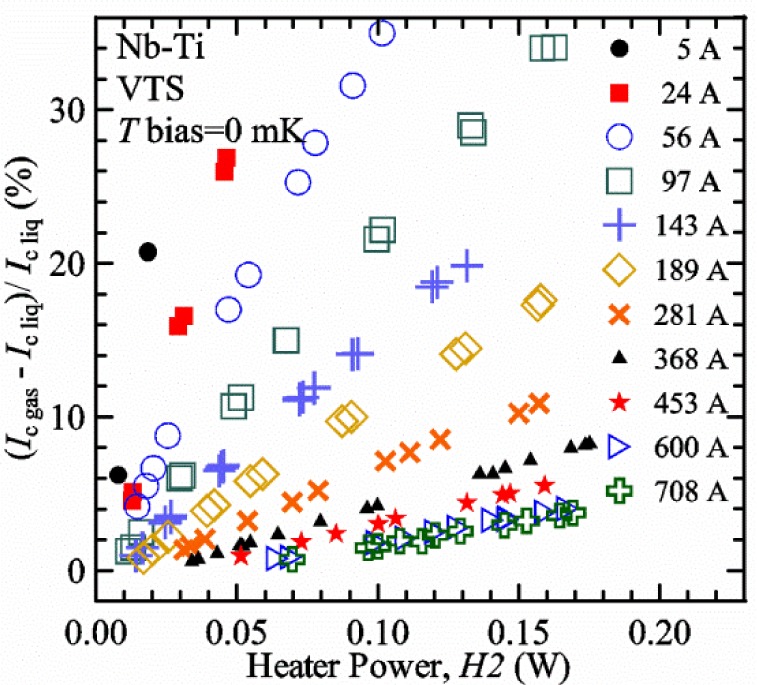
Percentage difference (*I_c gas_* − *I_c liq_*)/ *I_c liq_* versus *H2* at various magnetic fields where *I*_c_ varies from 5 A to 708 A at 5 K for a Nb-Ti wire soldered to a Cu-Be spring on the VTS probe. *T* bias (*T1−T2*) was set to 0 mK.

**Fig. 50:**
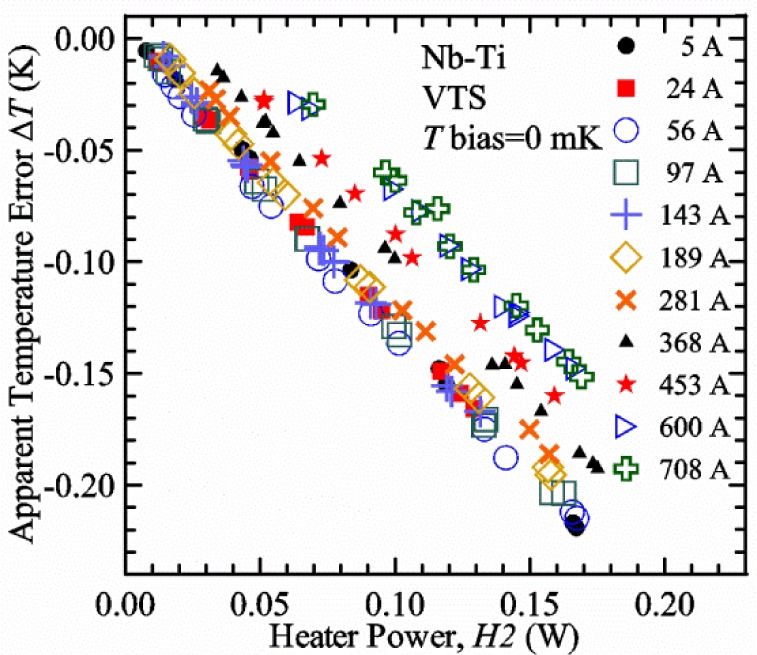
Apparent temperature error Δ*T* versus *H2* at various magnetic fields where *I*_c_ varies from 5 A to 708 A at 5 K for a Nb-Ti wire soldered to a Cu-Be spring on the VTS probe. *T* bias (*T1−T2*) was set to 0 mK. These data are from the same set shown on Figs. 48 and 49.

**Fig. 51:**
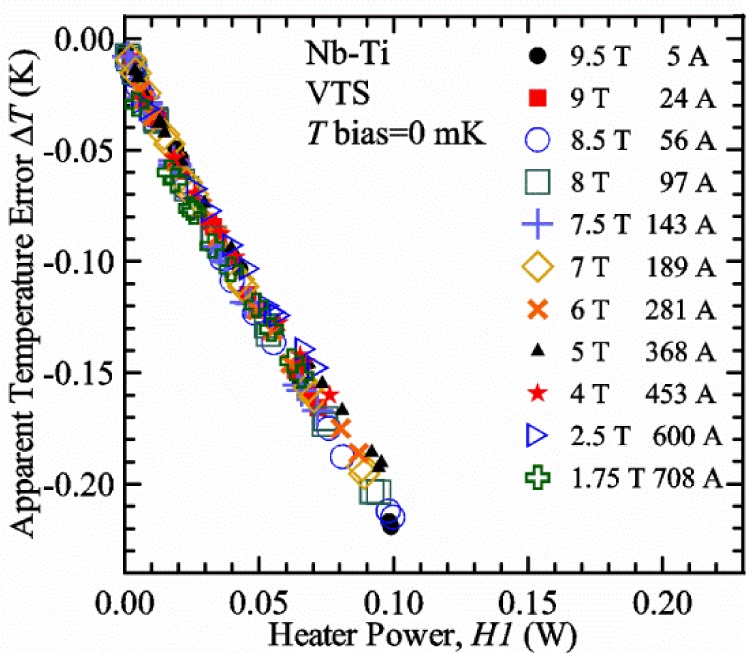
Apparent temperature error Δ*T* versus *H1* at various magnetic fields where *I*_c_ varies from 5 A to 708 A at 5 K for a Nb-Ti wire soldered to a Cu-Be spring on the VTS probe. These data are from the same set shown on Figs. 48–50.

**Fig. 52:**
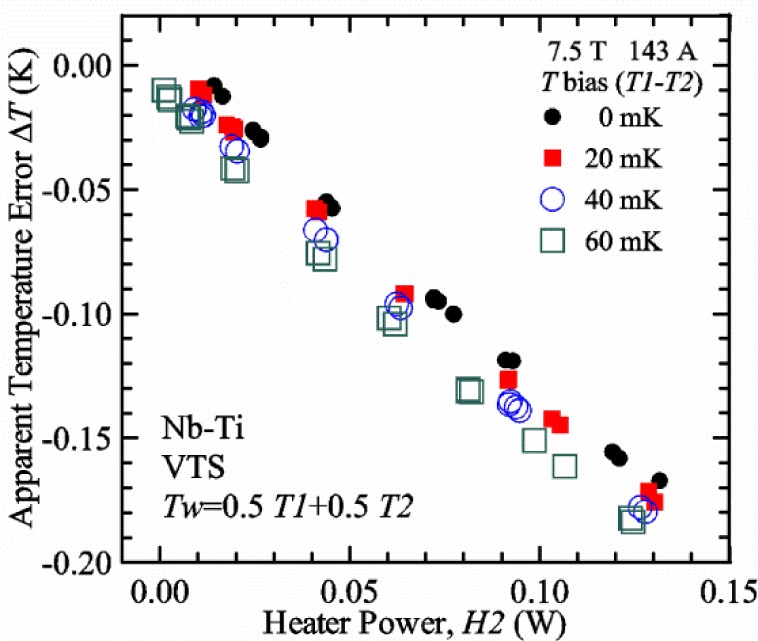
Apparent temperature error Δ*T* versus *H2* at 7.5 T and *T2* = 5 K where *I*_c_ is about 143 A for a Nb-Ti wire soldered to a Cu-Be spring on the VTS probe. The *T* bias (*T1−T2*) was set to 0, 20, 40, and 60 mK. The weighted temperature *Tw* = 0.5 *T1*+0.5 *T2* was used to determine the correct *I*_c_.

**Fig. 53:**
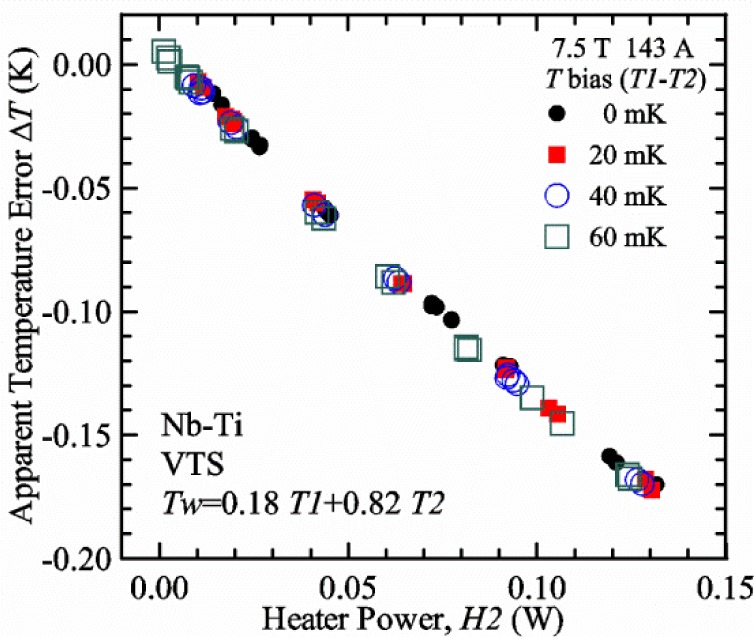
Apparent temperature error Δ*T* versus *H2* at 7.5 T and *T2* = 5 K where *I*_c_ is about 143 A for a Nb-Ti wire soldered to a Cu-Be spring on the VTS probe. The *T* bias (*T1−T2*) was set to 0, 20, 40, and 60 mK. The weighted temperature *Tw* = 0.18 *T1*+0.82 *T2* was used to determine the correct *I*_c_, which collapsed the Fig. 52 data onto one line.

**Fig. 54:**
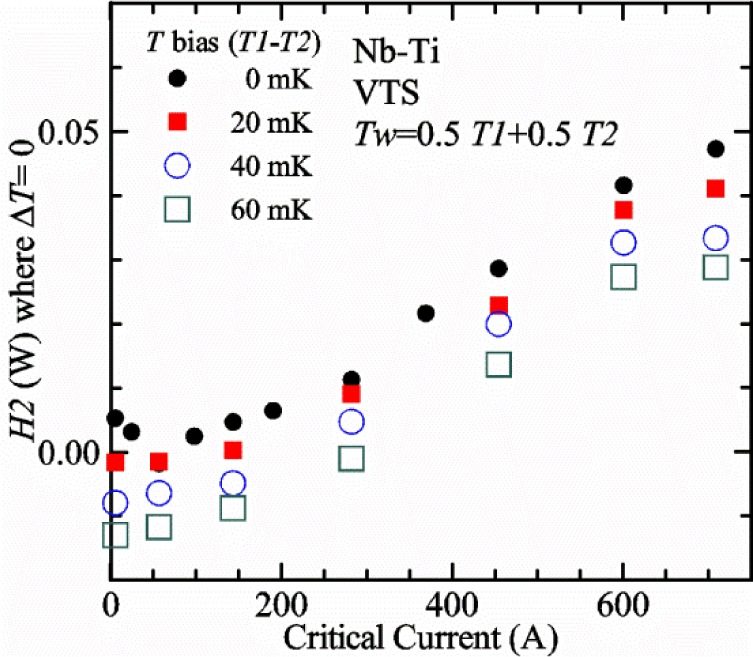
Plot of *H2* where Δ*T* = 0 versus *I*_c_ at 5 K for a Nb-Ti wire soldered to a Cu-Be spring on the VTS probe. The *T* bias (*T1−T2*) was set to 0, 20, 40, and 60 mK. The weighted temperature *Tw* = 0.5 *T1*+0.5 *T2* was used to determine the correct *I*_c_.

**Fig. 55:**
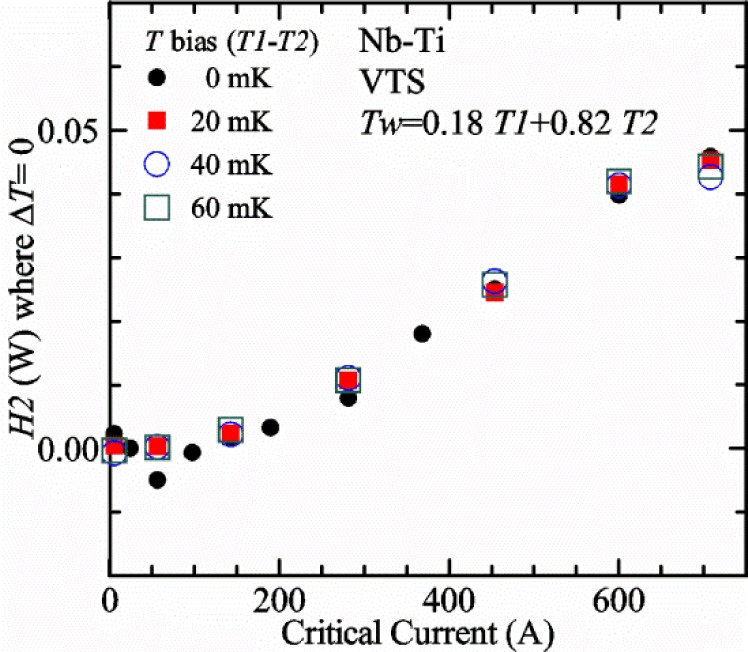
Plot of *H2* where Δ*T* = 0 versus *I*_c_ at 5 K for a Nb-Ti wire soldered to a Cu-Be spring on the VTS probe. The *T* bias (*T1−T2*) was set to 0, 20, 40, and 60 mK. The weighted temperature *Tw* = 0.18 *T1*+0.82 *T2* was used to determine the correct *I*_c_, which collapsed the Fig. 54 data onto one curve.

**Fig. 56:**
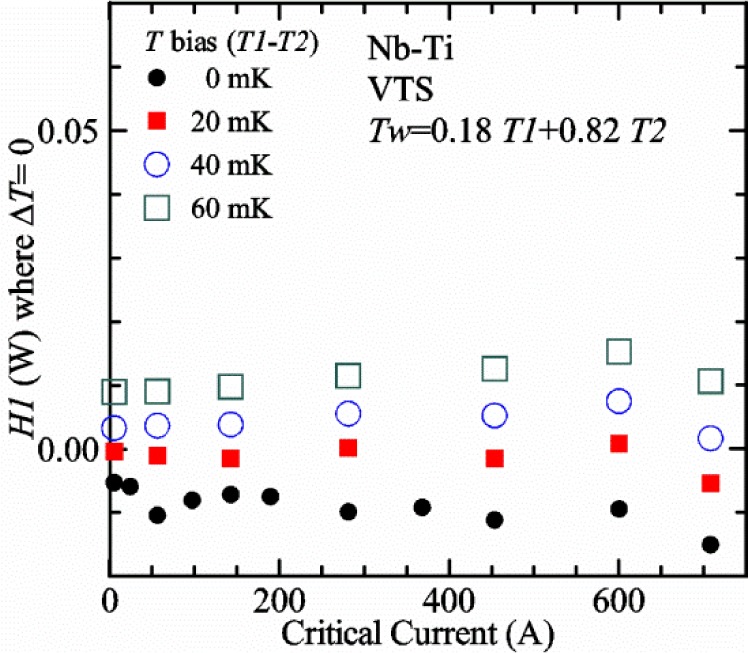
Plot of *H1* where Δ*T* = 0 versus *I*_c_ at 5 K for a Nb-Ti wire soldered to a Cu-Be spring on the VTS probe. The *T* bias (*T1−T2*) was set to 0, 20, 40, and 60 mK. The weighted temperature *Tw* = 0.18 *T1*+0.82 *T2* was used to determine the correct *I*_c_. *T* bias of 40 mK or higher allows *H1* to be positive when Δ*T* = 0.

**Fig. 57:**
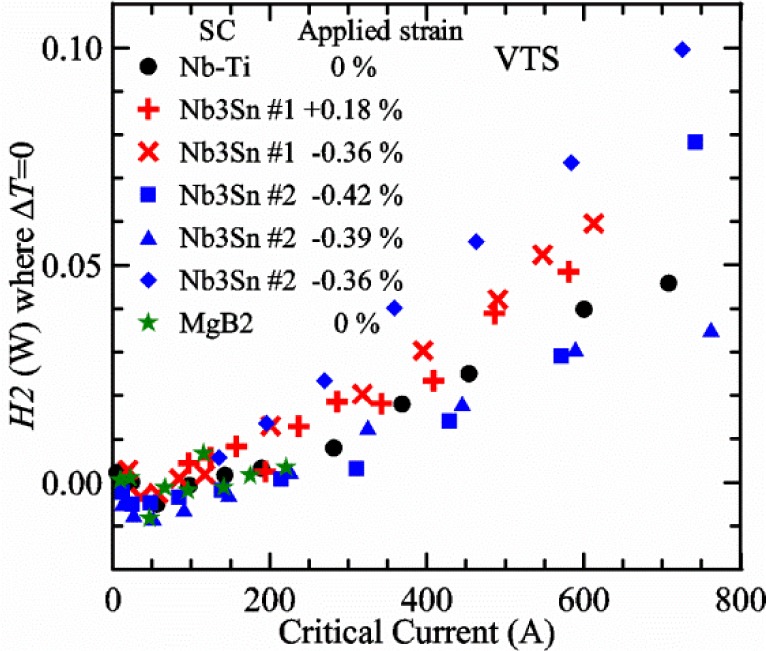
Plot of *H2* where Δ*T* = 0 versus *I*_c_ for four samples that were soldered to the spring of the VTS probe. Some of the samples were measured under different applied strains and conditions. This is part of the correct protocol for VTM.

**Fig. 58:**
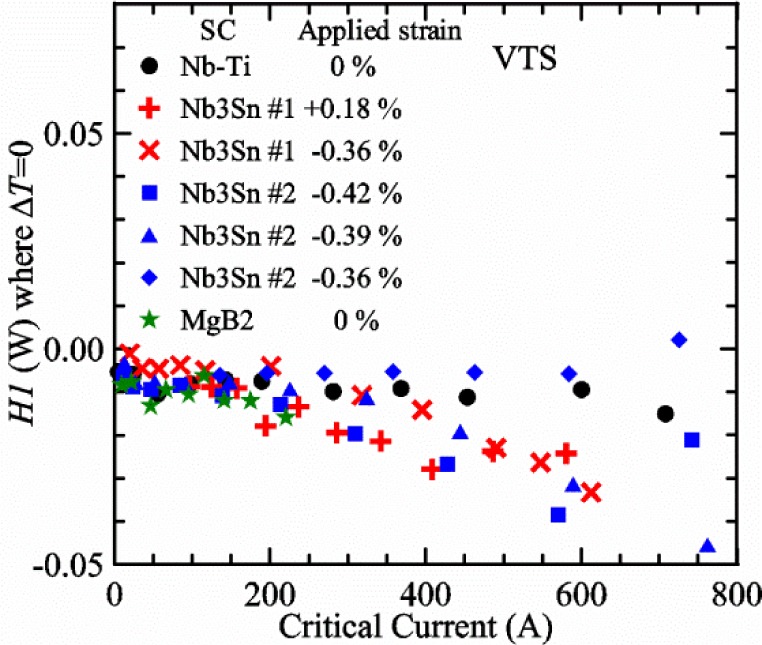
Plot of *H1* where Δ*T* = 0 versus *I*_c_ for four samples that were soldered to the spring of the VTS probe. Some of the samples were measured under different applied strains and conditions.

**Fig. 59:**
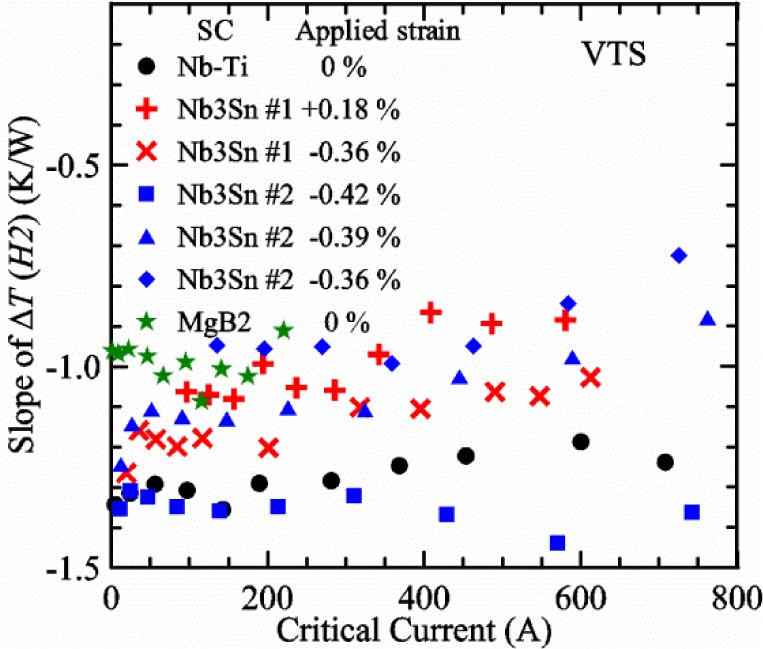
Plot of slope of the Δ*T*(*H2*) versus *I*_c_ for four samples that were soldered to the spring of the VTS probe. Some of the samples were measured under different applied strains and conditions.

A plot of the slope of Δ*T*(*H2*) in units of K/W versus *I*_c_ is shown in Fig. 59 for the seven cases studied on the VTS probe. All of these data are based on *liquid*/*gas* comparisons at 5 K. We have also studied the slope of Δ*T*(*H2*) at temperatures above 5 K for the VTS probe and found that the slope is very similar to that at 5 K, which is just as we observed for the VTO probe as shown in Fig. 33. For the VTS probe, the average slope is −1.11 K/W and most of the values fall within a 0.6 K/W range. The slopes for the case with the least bypass flow (square symbol in Fig. 59) are the most negative, and the slopes with the most bypass flow (triangular symbol) are close to the upper band of values. This difference may or may not be significant.

**Fig. 60:**
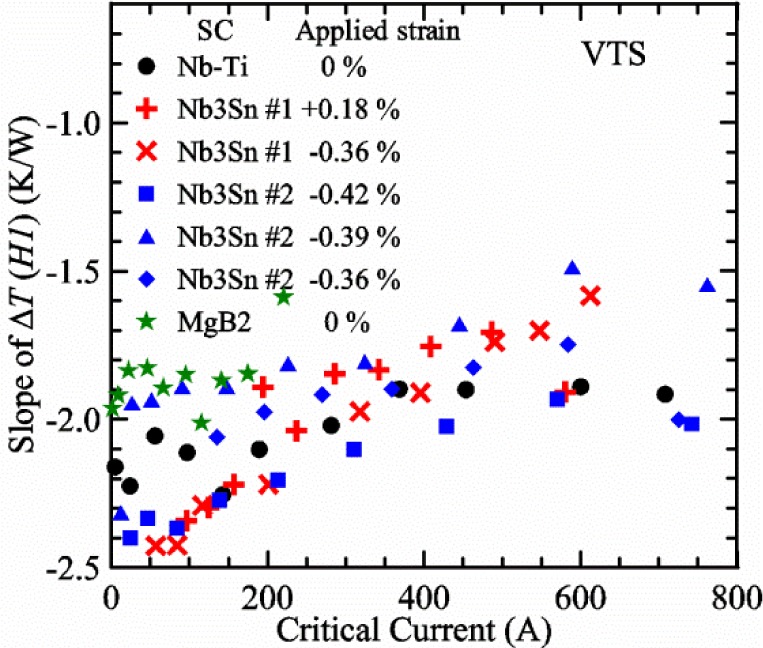
Plot of slope of the Δ*T*(*H1*) versus *I*_c_ for four samples that were soldered to the spring of the VTS probe. Some of the samples were measured under different applied strains and conditions.

A plot of the slope of Δ*T*(*H1*) in units of K/W versus *I*_c_ is shown in Fig. 60 for the seven cases studied on the VTS probe. All of these slopes are within a range of about 0.6 K/W and show a tendency to become less negative with increasing *I*_c_. The average slope is −2.00 K/W.

## 4. Discussion

### 4.1 Commercial Variable Temperature Inserts and Non-Transport *I*_c_ Measurements

A number of commercial variable temperature inserts (VTIs) fit inside a superconducting magnet and use a flow of helium gas to cool the sample space. Typically, the gas flow rate of these systems is designed for a small heat load that operate only with sample currents of 1 A to 10 A maximum. The advantage of a commercial VTI for appropriate low-power measurements makes these systems the clear choice. They can be purchased with a complete set of hardware and fully automated software to do basic measurements such as AC and DC resistivity, Hall effect, heat capacity, DC magnetization, AC susceptibility, vibrating sample magnetometry, thermal conductivity, and critical current measurements. Systems are sold with various superconducting magnets up to 16 T. Commercial VTIs are as close to turn-key as cryogenic experiments can be. In addition, they can be customized platforms to do other low-power measurements. If an experiment could be performed in a commercial VTI, it is likely the most practical and efficient choice. Some newer systems are available cryogen-free, which means that they operate with very little, if any, liquid cryogen. They typically use just a small charge of high-purity helium gas in a closed-cycle cryocooler that cools the superconducting magnet and the sample space. Nonetheless, at this point, we do not know of any commercial VTI that has been converted to measure critical current at currents of 100 A or higher with temperature control on the order of 0.1 K or better.

Non-transport, contactless measurements of *I*_c_ can be made using a commercial VTI by measuring the magnetization of a superconducting sample as a function of magnetic field and applying the critical state model. This can be done at various temperatures to map the surface of *I*_c_(*H*,*T*). Experiments like this were done in 1969 [16] and the variable temperature data that they provided were some key early results. Commercial VTIs were not available in 1969, but these measurements could be easily and efficiently done now in these systems. The sample and holder are very small and there is very little self-heating or heat load. The measurements can be done much faster and more autonomously than the transport measurements. The main difficulty with VTI measurements is estimating the *I*_c_ from the magnetization data, which requires a model and detailed information about the sample geometry and configuration of the superconductor within the composite conductor. There are often a number of simplifying assumptions that need to be made in order to derive the relationship between the magnetization and the *I*_c_. In many cases, derived values are compared to, or scaled with, transport results where possible. Results from magnetization studies are useful; however, the superconductor community still relies on transport *I*_c_ measurements for definitive characterization.

### 4.2 Similarities and Differences Between Two NIST Probes, VTO and VTS

A number of components of the VTM system are the same when using each of the two probes. The same control, data acquisition, and analysis software are used. The same instrumentation, magnet and magnet dewar, re-entrant dewar, and pre-regulator are used with both probes. The same size vapor-cooled leads and type of heaters and thermometers are used.

The main result of the *liquid*/*gas* comparisons at 5 K was the relationship between *H2* where Δ*T* is zero and *I*_c_. For both probes, the *H2* target needs to be near zero power for *I*_c_ < 150 A and the *H2* target increases nonlinearly with current. This general result did not depend on the type of sample, and in the case of the VTO probe, it did not depend on whether the sample was soldered to the mandrel or not. The *H2* target powers at higher current were somewhat different for the two probes, but the difference may depend on just current contact- and self-heating, and how much of current contact heating flows toward the sample versus how much flows to the terminals and heaters. The flow of the current contact heating was too uncertain to perform a quantitative comparison of contact heating between probes and even between different runs on the same probe. The additional variables of heat distribution along the contact and its dispersion reduced these comparisons to qualitative at best, which is why there is very little discussion of contact resistance in this paper other than that less contact resistance is better.

The general shapes of *H1* where Δ*T* is zero versus *I*_c_ were very different for the two probes, mainly because *H1* in the VTS probe has only downstream cooling. Thus, any cooling power that the gas would have as it flows over *H1* would mean that the sample was sub-cooled. To make matters worse, the data suggested that *H1* often needed to be negative for all *I*_c_, which is not possible, and means that *T1* needs to be controlled at a higher temperature than *T2* to keep *H1* positive. This lead to the *T* bias (*T1*–*T2*) experiments and the determination of the appropriate weighted temperature, *Tw* = 0.14 *T1* +0.86 *T2* for VTO and *Tw* = 0.18 *T1* +0.82 *T2* for VTS. The weighting coefficients were very similar for the two probes; however, the *T* bias necessary to change *H1* relative to *H2* was larger for the VTS probe. In general, we do not use a *T* bias for the VTO probe, but it is useful to use a *T* bias of about 30 mK or more for the VTS probe. If one sets *T1* = *X*+0.0246 and *T2* = *X*-0.0054, then *Tw* = *X* using the VTS equation. This example shows that the *T* bias adjustment has a small effect on *T2* and potential changes in these coefficients would not introduce significant temperature effects.

The slopes of Δ*T*(*H2*) and Δ*T*(*H1*) for the two probes were different by a factor of about 2.5 in the case of Δ*T*(*H2*) and a factor of about 4 in the case of Δ*T*(*H1*). For a given probe, these slopes did not vary much with temperature. The significance of the Δ*T*(*H2*) slope is that it indicates how much *H2* power will alter the sample temperature. We have no quantitative understanding of why these slopes are larger for the VTS probe. One measurable factor is how much the temperature set point of the pre-regulator needs to change to cause the total sample heater power (*H1*+*H2*) to change by 0.1 W for each of the systems with the same gas flow rate. For the VTS probe, the change in set point necessary was higher by more than a factor of 2 than that for the VTO probe, indicating that the difference between the two probes is due to the much smaller cooling surface of the VTS probe. For both probes the respective slopes of Δ*T*(*H2*) and Δ*T*(*H1*) did not change with temperature, although we did not show these data for the VTS probe.

The coil sample pitch lengths (3.2 mm for VTO and 6.4 mm for VTS) are different by a factor of 2 for the two probes. The longer pitch length spreads the contact heating over more cooling surface. In addition, the ends of the Cu-Be spring may reduce the amount of contact heating that flows to the active portion of the sample. These two factors may have caused the *H2* where Δ*T* is zero at high currents to be less for the VTS probe than for the VTO probe. Another factor is that the thermometers and heaters are farther apart in the VTS probe, which also gives more cooling surface for the sample and current contacts.

### 4.3 Brief Description of Other Laboratories’ Variable Temperature Systems

In this section we provide a very brief review of VTM systems used by other laboratories to make transport critical-current measurements. We will discuss the perceived advantages and disadvantages of each approach. We will also put these systems into the context of our VTM system and our general results. Our systems use a high gas-flow rate with a pre-regulator to keep most of the heat away from the sample area. The most important result of our study, simply stated, is the higher the heater power near the sample, the higher the temperature gradients. Other systems that use a stagnate gas at one atmosphere of pressure or at very low pressure may have temperature gradients that depend more on heater power than a flowing gas system. If the heater power near the sample needs to be increased with sample temperature, then temperature gradients and potential temperature biases will systematically increase with *T*. The relative placement of the heater(s), thermometer(s), and sample, and details about the heat flow, will be factors in how much the heater power will affect the measured *T* and *I*_c_. Numerous choices need to be made when designing a VTM system, and some of these choices result in trade-offs among complexity, temperature uncertainty, and current level. VTM of HTS samples can typically tolerate higher *T* uncertainties, unless, for example, precise determinations of the strain effect are needed. The cooling power needed for current leads and contact- and self-heating increases quickly with sample current above 100 A. The other VTM systems selected for review were found in a literature search that likely did not find all such systems. No slight was intended to those not cited. In many cases published articles do not go into the same level of detail as this paper, which limits the possible review and comparison.

#### Helium gas flow systems

Two VTM systems that use helium gas flow are at the University of Geneva [17,18] and at Kyoto University [19]. Both of these systems are VTS with a long sample length on a Walters spring device [13] and have thermometers above and below the sample. The University of Geneva has reported results up to about 300 A. Kyoto University sets the temperature of the gas flow 1 K to 2 K lower than the sample temperature. The gas flows from below the sample, indicating that a remote heater pre-regulates the flowing gas temperature. This probe also has sample heaters above and below the strain spring. This system, and perhaps the system at University of Geneva as well, seems to have a design that is the closest to our system with similar advantages and disadvantages. The setting of the gas temperature a fixed amount below the sample temperature would be simpler and less likely to become unstable than our more active control of sample cooling power. Kyoto University’s approach allows the sample heater power to be adjusted independent of the sample temperature and the sample heater power can be increased with current at a given temperature to provide cooling for sample contact- and self-heating. For each of these systems, it would be an interesting experiment to study the dependency of the measured *I*_c_ on sample heater power at a given *T* and *H*, indicating the sensitivity to this important parameter.

#### Inverted, insulating cup

Another design uses an inverted, insulating cup that is placed over the sample that is in a liquid helium bath. The cup is naturally full of helium gas at atmospheric pressure. Sometimes this type of device is called a bathysphere [20]. The lower part of the cup is typically insulated with a solid plug and some of the temperature difference between the sample and the surrounding bath occurs across this plug. The rest of the temperature gradient occurs across the helium gas. The University of Twente has three different systems that use this approach [21–24]. One system [21] has a U-shaped spring to vary the sample strain. A second system [22] has a coil sample on a Ti-6-4 barrel with heaters and thermometers above and below the coil sample. This is a VTO probe. The third system [23,24] has a single turn sample on a planar spring that is called a Pacman because of its shape. Sample heaters are located near the current contacts to provide a quick response to contact heating. The main advantages of the inverted cup design are its simplicity, sample cooling provided by a full atmosphere of helium gas, and cooling for the current leads and mechanical components is easily provided by the surrounding helium bath. The mechanical and electrical feed-throughs to the sample do not need to go through a vacuum seal; however, they typically need to go around the outside of the cup and up from the bottom. *I*_c_ values are reported to greater than 600 A.

As with other systems, it would be an interesting experiment to study the measured *I*_c_ dependency on sample heater power at a given *T* and *H*. It may not be possible to do this experiment in these systems as they are presently configured; however, it could be done by adding a Cu shield with an independent heater and thermometer. The Cu shield can have openings for the mechanical and electrical components; it just needs to be capable of intercepting some of the cooling power and provide a more isothermal environment for the sample. A different approach may be to place heaters on various mechanical and/or electrical couplers in the temperature gradient zone, but away from the sample. These heaters could be used to balance some of the steady-state conductive heat flow from the sample to the bath to establish the temperature gradient away from the sample. Systematically varying the power of these heaters and measuring the resulting *I*_c_ at a given *T* and *H* would indicate the relative importance of the sample heater-power parameter for inverted cup systems.

#### Vacuum can with helium exchange gas

There are too many examples of vacuum-can designs with helium heat-exchange gas to review each in detail [12, 25–31]. Typically in this approach, the current leads enter the magnet dewar helium bath, and then go through a vacuum feed-through into a can that is inside the bore of the magnet. In some cases, the vacuum space extends to a space at room temperature where mechanical and/or electrical feed-throughs are located. Even in the case of an extended vacuum space, the sample current may still go through the magnet dewar to provide the necessary cooling for the high-current leads, which has the advantage that the heat-load and heat generated by the sample current leads do not have a direct impact on the temperature control. The sample is held in the center of the field within the vacuum can. A controlled, small amount of helium is introduced into the vacuum space to act as a heat-exchange gas between the sample and the helium bath that surrounds the vacuum can. The sample current leads also conduct heat from the sample to the bath. Designing for high-current does increase the lead conduction to the bath. One of the typical disadvantages of the vacuum-can approach is creating a low-temperature, vacuum-tight seal for each sample.

A 1979 paper [25] showed impressive results to 1,200 A with a sample in a vacuum can. In general, other vacuum-can systems are limited to 300 A or less. A key feature of the system in [25] was a “hairpin” shaped sample that had only a short portion of the sample in perpendicular magnetic field. The rest of the sample was in parallel field where the temperature margin was higher and most of the contact heating could be handled. The “hairpin” geometry allows for a short sample length while reducing the effects of contact heating and current-transfer voltages. An advantage to having a short sample is that it is easier to control and measure the sample temperature. A capacitance thermometer was used for temperature control and three resistive thermometers were used to measure the temperature at different locations along the sample. There will be some current-transfer voltages along the portion of the sample that is in perpendicular magnetic field even though there is a long length in parallel field [32]. This will limit the sensitivity of the critical-current criterion for some samples, which is a general disadvantage for short sample lengths.

An early comprehensive paper from the University of Durham [12] describes their VTS system that uses a vacuum can. This system uses a Walters spring with a long sample length and a long copper cylinder with a heater around the spring. A thermometer was located on the spring next to the sample. The maximum current was about 25 A. This paper also mentions recent improvements that increased the maximum current to nearly 150 A when the sample is not immersed in liquid helium. These improvements included adding two more thermometers (one on each end of the sample) and three independent heaters for more uniform sample temperature.

An earlier paper describes a different vacuum-can system from the University of Durham [26] states that the heater was placed 15 cm from the sample to reduce temperature gradients along the sample, which is an advantage. However, a 15-cm separation also means that the heater cannot quickly respond to contact heating, which is a disadvantage. The PID parameters of the heater will also need to be kept slow to avoid temperature oscillations.

As with other systems, it would be an interesting experiment to study the measured *I*_c_ dependency on sample heater power at a given *T* and *H*. Adjusting the pressure of the exchange gas may allow different heater powers at the same temperature, but there is a range of pressure over which heat conduction is fairly independent of pressure due to the additional effect of the mean-free path that decreases with increasing pressure. Adding a Cu shield with an independent heater and thermometer could allow one to independently vary the heater power near the sample.

#### Supercritical helium

A few systems use supercritical helium as a coolant [33–36]. When the helium is at a temperature and pressure above the critical point, the helium is in the supercritical fluid state where distinct liquid and gas phases do not exist. In the referenced systems a flow of supercritical helium provides the cooling since no liquid-to-gas phase transition provides cooling. In [33] the operating pressure of the fluid is up to 0.29 MPa (42 psi) and the fluid flows over the current leads near the sample and then out the current leads to room temperature. This provides cooling for each end of the sample by thermal conduction down high-purity copper bus bars while the sample is isolated in a vacuum space. The cooling power is adjusted by a heater in the flowing fluid to achieve temperatures from 5 K to 20 K. *I*_c_ values are reported up to 600 A with temperature accuracy of ± 0.025 K up to 8 K and ± 0.05 K up to 17 K.

The SUpraLeitierTest ANlage (SULTAN) facility [34–36] is used to test large cable-in-conduit conductors (CICC) with forced-flow, supercritical helium cooling through the CICC samples. The CICC samples are cables that contain several hundred superconductors and, in some cases, additional copper wires (strands) in parallel. There is enough void area inside the conduit to allow the fluid to flow through the total length of the conductor. The operating pressure of the fluid is up to 1 MPa (145 psi) with a helium mass-flow rate of 1 g/s to 10 g/s. Sample currents up to 100,000 A can be achieved with a background magnetic field up to 10.85 T. Eighty channels of data are acquired during the sample testing. Comparisons between voltage tap and calorimetric measurements give agreement to within ± 0.1 K. This is clearly one of the largest and state-of-the art variable-temperature measurement systems for superconductors in the world.

## 5. Conclusions

Our approach to high-current, variable-temperature measurements is to cool the sample and current leads with a high-flow rate of helium gas, and to pre-regulate the cooling power of the gas with a heater that is located away from the superconducting sample. We use additional thermometers and heaters on each current contact, at end of the sample, for finer temperature control with low power levels near the sample. This approach reduces the temperature gradients between the sample thermometers and the relatively long sample. In our case, the samples are wires, 0.5 mm to 0.85 mm in diameter, with active lengths of more than 30 cm. In addition, the pre-regulator is located such that it will intercept a large fraction of the steady-state heat load flowing down the sample current leads and the heat load that is created when the sample current is on. Thus, most of the sample-current heat load simply replaces the pre-regulator heater power, which buffers the sample temperature control from the changing heat load conditions.

Direct comparisons of critical current measurements at 5 K with the sample immersed in pressurized liquid helium to those with the sample in flowing helium gas at 5 K allowed us determine the effect of, and to optimize, the measurement protocol. We demonstrated critical-current measurement results that are very repeatable from 4 A to 981 A with our variable-temperature only probe and from 5 A to 780 A with our variable-temperature, variable-strain probe.

**Key conclusions for our variable temperature only (VTO) and variable-temperature, variable-strain (VTS) systems are:**

- The results of the comparison of measurements in *liquid* and in *gas* at the same temperature were independent of type of superconductor, although the Nb-Ti sample was more sensitive to temperature than the Nb_3_Sn and MgB_2_ samples. In the case of our VTO probe, the results were the same regardless of whether or not the sample was soldered to a mandrel.
- Our primary sample heater power (*H2*, bottom contact) is an indicator of the cooling power of the flowing helium gas, and excess cooling power will sub-cool the sample relative to the sample thermometers. The apparent temperature errors increased systematically with sample heater power.
- The *liquid*/*gas* comparison indicated that the correct protocol for the variable-temperature measurements is essentially to target a *H2* sample heater power that is a function of critical current. This also requires a dynamically changing *Pre-reg* power.
- For both of our systems, when the critical current is below 150 A, the “correct” measurements are obtained when the target *H2* power is near zero, <0.01 W. The target heater power increases nonlinearly with critical current.
- If the *H2* sample heater power is too high by 0.1 W, this will cause a temperature error of about 0.04 K for the VTO and 0.12 K for the VTS systems.
- The largest percentage of critical-current errors occur at lower currents (< 150 A) because of the high sensitivity of critical current to temperature.
- Our VTS system is about 2.5 times more sensitive to sample heater power than our VTO system. This may be due to less cooling surface in the VTS probe compared to the VTO probe.
- The cooling of the two-current contacts in our VTO system is more symmetric than for the VTS system because one of the current leads has to be rotated as strain is applied to the sample, so more space is occupied by mechanical components.
- The cooling of the top contact in our VTS system is mainly downstream from the bottom contact and thus the heating of the top contact needs to be less than 0.01 W to obtain the “correct” measured critical current, regardless of the current level. However, we can use a temperature set point for the top contact that is slightly higher than the bottom contact to maintain better temperature control.

**Key conclusions for other systems are:**

- Even small heater powers, less than 0.1 W, near the sample may cause temperature gradients of 0.04 K to 0.12 K depending on the details of the sample cooling. For a given conductor, this can have a bigger effect where the critical currents are low because of the higher sensitivity to temperature in those regions.
- If possible, it would be useful to measure how much the critical current varies with heater power near the sample at constant temperature to determine how sensitive the system is to heater power levels.
- If higher heater powers near the sample are necessary to achieve higher temperature, this could cause a systematically increasing bias with temperature. This could be reduced with a heated radiation shield between the sample and the cooling source.
- Interlaboratory comparisons of variable-temperature measurements would be useful to verify the performance of the different types of variable-temperature systems.

## Figures and Tables

**Fig. 3:**
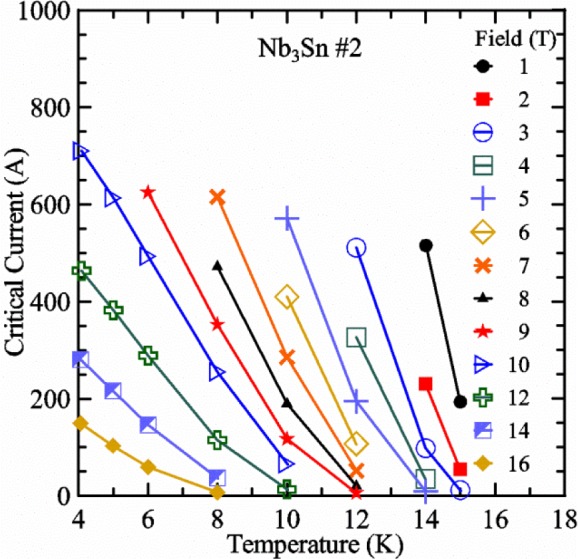
Critical current versus temperature at various magnetic fields for 0.70 mm diameter Nb_3_Sn sample #2.

**Fig. 4:**
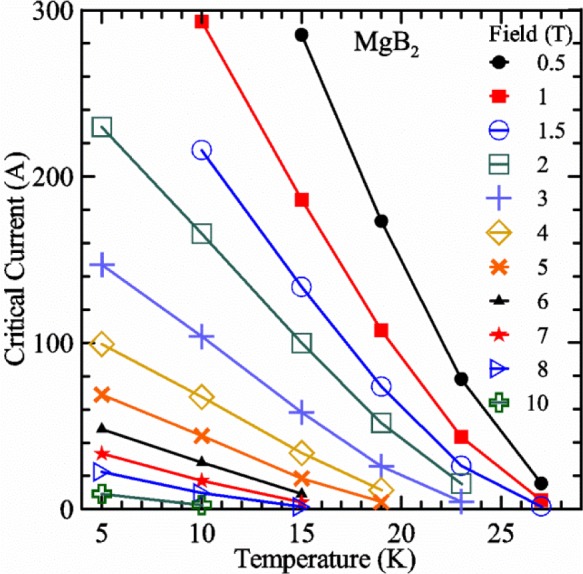
Critical current versus temperature at various magnetic fields for a 0.83 mm diameter MgB_2_ sample.

**Table 1:** Sample identification and wire parameters

Sample	Wire diameter (mm)	Fraction non-Cu	*I*_c_(A) at 4 K and 5 T	*I*_c_(A) at 4 K and 12 T	Applied strain in VTS
Nb-Ti	0.76	0.435	510	–	–
Nb_3_Sn #1	0.82	0.476	915	279	0.18%
Nb_3_Sn #2	0.70	0.538	*–*	464	0%
MgB_2_	0.83	0.16*	70	–	0%

* For MgB_2_ fill factor is shown instead of fraction non-Cu

## References

[1] Ekin JW (2010). Supercond Sci Technol.

[2] Goodrich LF, Fickett FR (1982). Cryogenics.

[3] Goodrich LF, Bray SL (1990). Cryogenics.

[4] Ekin JW (1978). J Appl Phys.

[5] Tachikawa K, Itoh K, Wada H, Gould D, Jones H, Walters CR, Goodrich LF, Ekin JW, Bray SL (1989). IEEE Trans Magn.

[6] Wada H, Walters CR, Goodrich LF, Tachikawa K (1994). Cryogenics.

[7] Wada H, Goodrich LF, Kate HJJ (1998). Adv in Superconductivity.

[8] Goodrich LF, Stauffer TC (2004). Adv Cryog Eng Mater.

[9] Goodrich LF, Cheggour N, Ekin JW, Stauffer TC (2007). IEEE Trans Appl Supercond.

[10] Goodrich LF, Cheggour N, Lu XF, Splett JD, Stauffer TC, Filla BJ (2011). Supercond Sci Technol.

[11] Brandt BL, Liu DW, Rubin LG (1999). Rev Sci Instrum.

[12] Cheggour N, Hampshire DP (2000). Rev Sci Instrum.

[13] Walters CR, Davidson IM, Tuck GE (1986). Cryogenics.

[14] Goodrich LF, Stauffer TC (2002). Adv Cryog Eng Mater.

[15] Goodrich LF, Ekin JW, Fickett FR (1982). Adv Cryog Eng Mater.

[16] Fietz WA, Webb WW (1969). Phys Rev.

[17] Seeber B, Uglietti D, Abacherli V, Bovier PA, Eckert D, Kubler G, Lezza P, Pollini A, Flukiger R (2005). Rev Sci Instrum.

[18] Uglietti D, Seeber B, Abacherli V, Carter WL, Flukiger R (2006). Supercond SciTech.

[19] Sugano M, Itoh K, Kiyoshi T (2006). IEEE Trans Appl Supercond.

[20] Moreland J, Li Y, Folsom R, Capobianco TE (1988). Rev Sci Instrum.

[21] Haken B, Godeke A, Kate HHJ (1993). IEEE Trans Appl Supercond.

[22] Haken B, Godeke A, Kate HHJ (1999). J Appl Phys.

[23] Godeke A, Dhallé M, Morelli A, Stobbelaar L, van Weeren H, van Eck HJN, Abbas W, Nijhuis A, den Ouden A, Haken B (2004). Rev Sci Instrum.

[24] Ilyin Y, Nijhuis A, Krooshoop E (2007). Supercond SciTech.

[25] Spencer CR, Sanger PA, Young M (1979). IEEE Trans Magn.

[26] Friend CM, Hampshire DP (1995). Meas Sci Technol.

[27] Hudson PA, Yin FC, Jones H (1981). IEEE Trans Magn.

[28] Nunoya Y, Isono T, Koizumi N, Hamada K, Nabara Y, Okuno K (2007). IEEE Trans Appl Supercond.

[29] Oh S, Lee C, Choi H, Moon K, Kim K, Kim J, Park PY (2008). IEEE Trans Appl Supercond.

[30] Schauer W, Zimmermann F (1980). Adv Cryo Eng.

[31] Finnemore DK, Ostenson JE, Gibson ED, Verhoeven JD, Doyle TB (1983). J Appl Phys.

[32] Goodrich LF (1983). IEEE Trans Magn.

[33] Martínez A, Duchateau JL (1997). Cryogenics.

[34] Bruzzone P, Anghel A, Fuchs A, Pasztor G, Stepanov B, Vogel M, Vecsey G (2002). IEEE Trans Appl Supercond.

[35] Bruzzone P, Stepanov B, Wesche R, Salpietro E, Vostner A, Okuno K, Isono T, Takahashi Y, Kim HC, Kim K, Shikov AK, Sytnikov VE (2008). IEEE Trans Appl Supercond.

[36] Bruzzone P, Stepanov B, Wesche R, Ilyin Y, Herzog R, Calvi M, Bagnasco M, Cau F (2009). IEEE Trans Appl Supercond.

